# Cyclization of a 1,8‐Diphenylanthracene by Scholl Reactions to Form Five‐ and Seven‐Membered Ring Embedded Polycyclic Aromatic Hydrocarbons

**DOI:** 10.1002/chem.71449

**Published:** 2026-07-20

**Authors:** Tatsuhiro Sasaki, Eiji Tsurumaki, Shinji Toyota

**Affiliations:** ^1^ Department of Chemistry School of Science Institute of Science Tokyo Tokyo Japan

**Keywords:** arenes, aromaticity, cyclodehydrogenation, molecular structure, Scholl reaction

## Abstract

Scholl reactions of 10‐mesityl‐1,8‐diphenylanthracene with DDQ (or chloranil) in TfOH/CH_2_Cl_2_ yielded three cyclized products depending on the equivalent of the oxidant. The singly cyclized product was a benzo[*a*]fluoranthene derivative, which further cyclized to the doubly cyclized product by formation of a seven‐membered ring. Reactions with a large amount of DDQ yielded a deep red product, which was identified as an unexpected triply cyclized product with a rubicene framework formed via the migration of a methyl group in the mesityl group. The molecular structures and aromaticity of these five‐ and seven‐membered ring products were investigated by X‐ray analysis and DFT calculations. The optical and electrochemical properties depend on the HOMO–LUMO gap, which decreases as the cyclization proceeds. The rubicene formation is proposed to proceed via an arenium ion intermediate and a 1,2‐methyl shift step.

## Graphical abstract

Reactions of 10‐mesityl‐1,8‐diphenylanthracene with DDQ/TfOH produced three products via sequential formation of a five‐ or seven‐membered ring. Unexpectedly, the triply cyclized product with a rubicene substructure was formed through cyclization involving 1,2‐methyl shift. The structures and properties of these new aromatic compounds were evaluated using spectroscopic and theoretical data.

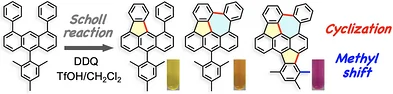

## 1 Introduction

Scholl reactions are a convenient method for the synthesis of polycyclic aromatic hydrocarbons through oxidative cyclodehydrogenation [1, 2, 3, 4, 5]. For example, triphenylene, perylene, and hexa‐*peri*‐benzocoronene (HBC) derivatives were synthesized through the reactions to create novel aromatic compounds [6, 7, 8, 9, 10, 11]. Because the Scholl reaction is useful for the formation of five‐membered rings, a large number of *peri*‐benzo fused arenes **B**, such as benzo[*a*]fluoranthene [12], were synthesized from phenyl‐substituted arenes **A** (Figure 1a) [13, 14, 15, 16]. This reaction has advantages over the cross coupling and Friedel–Crafts reactions so far applied, which require a functional group (X or Y) at the cyclized position in starting materials [12, 17, 18, 19, 20]. We reported that the Scholl reaction of 9,10‐diphenylanthracene (**1**) with 2,3‐dichloro‐4,5‐dicyano‐1,4‐benzoquinone (DDQ) in CH_2_Cl_2_ in the presence of trifluoromethanesulfonic acid (TfOH) readily yields rubicene (**2**), which is a promising aromatic core for functional organic materials [21, 22, 23, 24, 25], by formation of two five‐membered rings (Figure 1b) [26]. This reaction was further applied to the synthesis of extended rubicene‐based π‐conjugated compounds [27]. These studies inspired us to use 1,8‐diphenylanthracene (**3**) as a starting material for the Scholl reaction, because one phenyl group can undergo cyclization, followed by additional cyclization to another unreacted phenyl group [28]. One possible reaction is the formation of a seven‐membered ring across the phenyl groups to form a fused five‐ and seven‐membered (5/7) ring system in **4** (Figure 1c). A large number of derivatives with one or more 5/7 ring systems, namely azulene‐embedded aromatic compounds, have been studied as nonbenzenoid nanographenes [29, 30, 31, 32, 33, 34, 35], which are regarded as azulene‐defective graphenes [36, 37]. The expected structure **4** constitutes a substructure of circumazulene (**5**) [38, 39, 40, 41, 42], an azulene version of circumarenes that are defined as PHAs with a central aromatic core completely surrounded by one outer ring of fused benzenoid rings [43, 44, 45]. Although circumazulene is a novel nonbenzenoid aromatic compound, its parent compound remains unknown and only a few derivatives have been confirmed by on‐surface synthesis [38, 39, 40, 41, 42]. Therefore, the construction of framework **4** can be a key compound in a synthetic approach to circumazulene. We carried out the Scholl reaction of 10‐mesityl‐1,8‐diphenylanthracene (**6**) (mesityl: 2,4,6‐trimethylphenyl) in this study (Figure 1d). One of the cyclized products was an unexpected rubicene derivative formed by triple cyclization involving migration of a methyl group. Such methyl migration was observed for a few mesityl‐substituted compounds through electrophilic substitution [46, 47, 48, 49, 50]. Although Scholl reactions occasionally accompany some types of rearrangements such as aryl migration [51, 52, 53, 54, 55, 56], examples of methyl migration are quite limited [57]. In this study, we report the Scholl reactions of 1,8‐diphenylanthracene **6**, and the structures and properties of three cyclized products with five‐ and seven‐membered rings.

**FIGURE 1:**
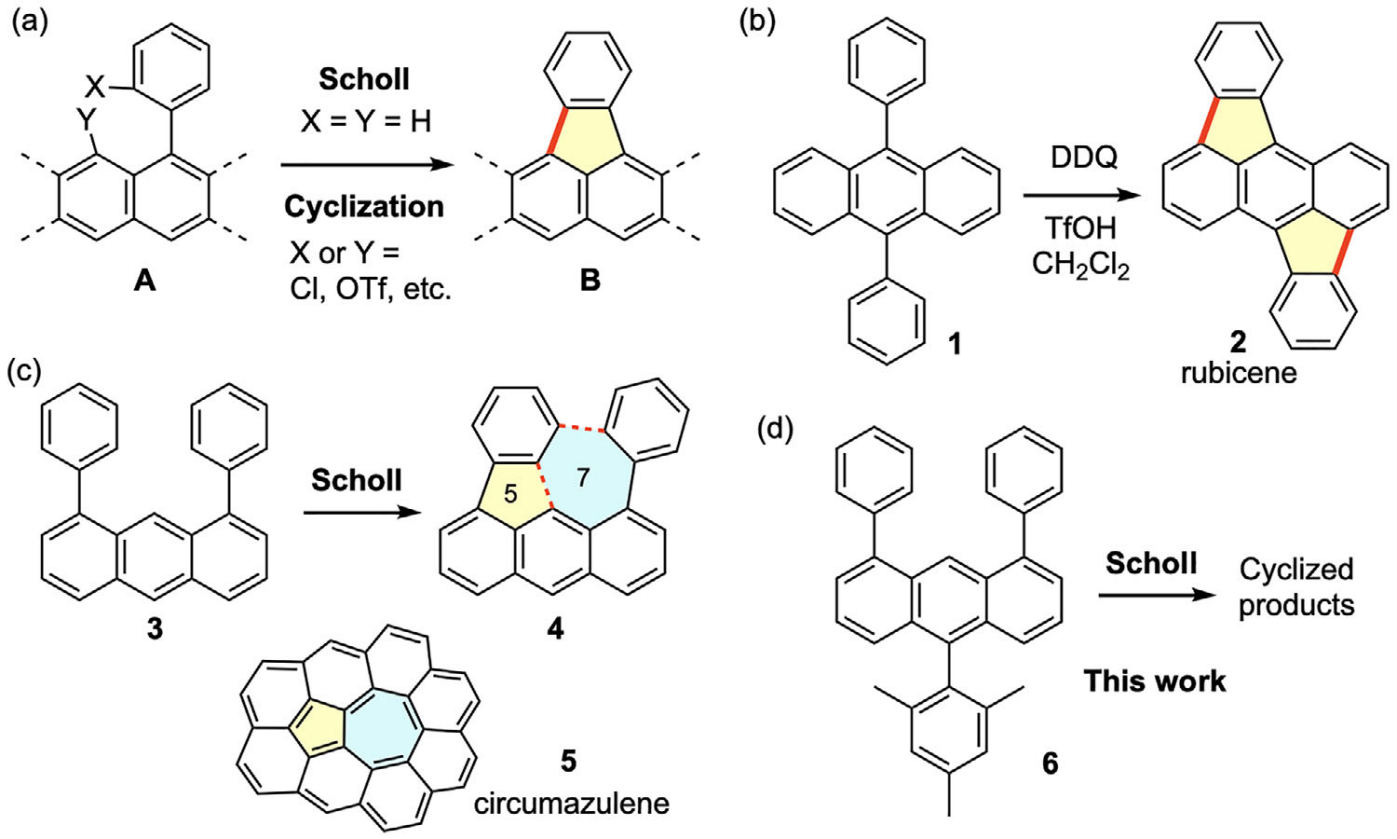
(a) Formation of a five‐membered ring by cyclization of phenyl‐substituted arenes, (b) synthesis of rubicene by Scholl reaction, (c) expected cyclization of 1,8‐diphenylanthracene, and (d) Scholl reaction in this work.

## 2 Results and Discussion

### 2.1 Scholl Reactions of Compound **6**

We selected compound **6** as a 1,8‐diphenylanthracene precursor, in which a mesityl group was introduced to maintain solubility and to block the reactive 10‐position [58]. This compound was prepared by the Suzuki–Miyaura coupling of 1,8‐dichloro‐10‐mesitylanthracene and phenylboronic acid [59]. We performed the Scholl reaction of **6** with 2,3‐dichloro‐4,5‐dicyano‐1,4‐benzoquinone (DDQ) in the presence of TfOH in CH_2_Cl_2_ (Figure 2), which is a typical condition for similar reactions [26, 27]. The results of the reactions with various equivalents of DDQ are listed in Table 1. When compound **6** was reacted with 2.0 equiv. of DDQ at 0°C for 2 h, we obtained singly cyclized product **7** as a yellow solid in 75% isolated yield (entry 1). This product is formed by the formation of a five‐membered ring between one phenyl group and the anthracene group.

**FIGURE 2:**
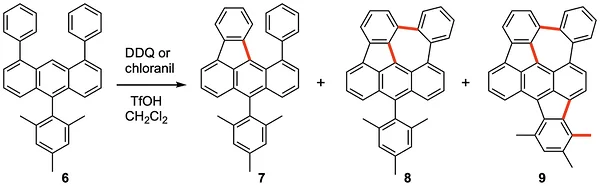
Scholl reactions of 1,8‐diphenylanthracene **6** with DDQ in TfOH/CH_2_Cl_2_. The newly formed C─C bonds are highlighted in red.

**TABLE 1:** Scholl reactions of **6** with DDQ or chloranil in the presence of TfOH in CH_2_Cl_2_.^a^

Entries	Oxidants	Equiv.	Product NMR Yield/% (isolated yield/%)^b^		
			7	8	9
1	DDQ	2.0	85 (75)	trace	9
2	DDQ	3.0	52	9	37
3	DDQ	3.5	18	22	42
4	DDQ	4.0	0	21 (19)	66 (60)
5	chloranil	2.0	70	8	0
6	chloranil	3.0	40	13	trace
7	chloranil	3.5	17	17	35
8	chloranil	4.0	trace	25	53

^a^ Reactions were carried out using **6** (10 mg) in CH_2_Cl_2_/TfOH 20:1 at 0°C for 2 h for analytical purposes.

^b^ NMR yields were determined by reading signal intensities relative to that of CH_3_NO_2_ as an internal reference.

The reaction of **6** with 4.0 equiv. of DDQ (entry 4) did not afford **7** but two other products, a minor product as an orange solid and a major product as a red solid. The minor product was identified as doubly cyclized product **8** having an extra seven‐membered ring via C─C bond formation across the phenyl groups. The structural determination of the major product was not straightforward from spectroscopic data. The structure was finally determined by the X‐ray analysis to be triply cyclized product **9** formed via migration of one of the *o*‐Me groups in the mesityl group. Reactions with a large amount of DDQ tend to produce highly cyclized products **8** and **9** (entries 1–4). These reactions also occurred with chloranil, which was an oxidant weaker than DDQ, in the presence of TfOH in CH_2_Cl_2_ (entries 5–8). The effects of the oxidant on the product distributions were rather small. In contrast, compound **6** did not react at all with DDQ only, TfOH only, or DDQ/MsOH (methanesulfonic acid). Therefore, both the oxidant and the very strong acid are essential for the cyclization reactions to proceed. We also carried out the reaction starting from **7**. When **7** was treated with DDQ (3.0 equiv.)/TfOH, compounds **8** and **9** were formed in 21% and 64% yields, respectively.

The mass and ^1^H NMR spectra suggested that the number of H atoms decreases by two each in the order of **6** (*m*/*z* 448) [59], **7** (446), **8** (444), and **9** (442). Namely, dehydrogenative cyclization occurs in each step. Compound **7** showed an aromatic signal due to the H1 atom at 6.08 ppm, which was strongly shielded by the ring current effect of the unreacted phenyl group. This signal disappeared in the ^1^H NMR spectra of **8** and **9**, where the H atom was lost by formation of a seven‐membered ring. Notably, compound **9** showed three Me signals of the same intensity in the aliphatic region. This is consistent with the molecular structure determined by the X‐ray analysis, in which one of the Me groups migrated upon formation of the five‐membered ring. The ^1^H NMR signals of **9** were fully assigned using COSY and NOE measurements. For example, significant signal enhancements by NOE were observed between Me11–H10 (19%), Me13–Me14 (8%), and Me14–H15 (13%) (Figure S16). The deshielding of the H10 atom (*δ* 8.83) is attributed to the steric compression with the adjacent Me11 group.

### 2.2 X‐Ray Analysis of Cyclized Products

X‐ray crystallographic analyses of **7**–**9** were performed with single crystals prepared by recrystallization from organic solvents. The ORTEP drawings are shown in Figure 3. Compound **7** has a benzo[*a*]fluoranthene framework, which was slightly twisted due to the presence of the phenyl group in the crowded region. The H1 atom is located in the shielding region of the phenyl group leading to the upfield shift of its ^1^H NMR signal. The mesityl group is nearly perpendicular to the attaching aromatic plane. In the structure of **8**, the five‐membered ring is nearly planar, whereas the seven‐membered ring is nonplanar, as suggested by the torsion angles around the ring. This nonplanar structure is attributed to the steric interaction between the peripheral H atoms (H4–H5 and H1–H14) in the bay regions. Accordingly, the fused benzo moiety bent away from the average plane of the benzo[*a*]fluoranthene framework. The C─C bond alternation around the seven‐membered ring is significant, where the two nonfused C─C bonds are longer than the others (Figure S1). The structure of the 5/7 ring system in **9** is similar to that in **8**. As shown in the side view, the rubicene moiety is slightly twisted.

**FIGURE 3:**
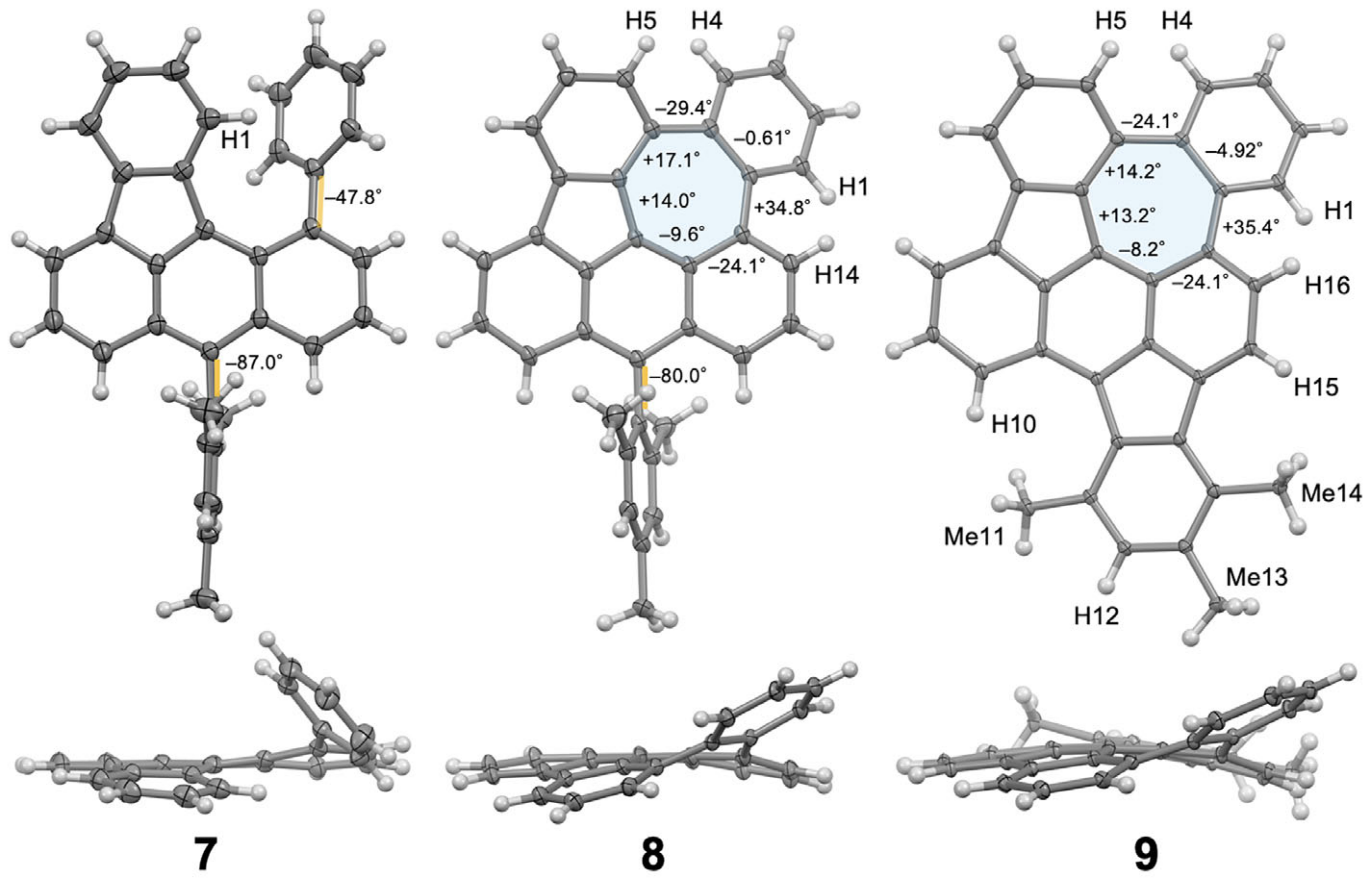
Two views of ORTEP drawings of X‐ray structures of **7–9** with thermal ellipsoids at 50% probability. One of the two independent molecules is shown for **7**. In the side views of **7** and **8**, the mesityl group is omitted. Torsion angles of the phenyl and mesityl groups and those within seven‐membered rings in **8** and **9** are shown.

### 2.3 Optical Properties

UV–vis and fluorescence spectra of compounds **6**–**9** were measured in CHCl_3_ (Figure 4 and Table S1). In the UV–vis spectra, the absorption maxima at the longest wavelength were considerably red‐shifted in the order of **6**, **7**, **8**, and **9** from 405 to 572 nm. The absorption edge of **9** reaches 600 nm, which is further red‐shifted relative to those of 2,4,6,9,11,13‐hexa‐*t*‐butylrubicene (**10**, 533 nm) [27], dibenzo[*a*,*m*]rubicene (**11**, ca. 510 nm) [60], and a fused dibenzo[*a*,*m*]rubicene derivative (**12**, 570 nm) [61] (see Table S1 for the structures of **10**–**12**). This significant shift is attributed to the additional benzo fusion to the rubicene core via the seven‐membered ring. A similar trend was observed in the fluorescence spectra as well. The emission bands were observed at 522, 546, and 602 nm for compounds **7**–**9**, respectively. In particular, the emission edge of **9** extends to 800 nm showing a characteristic pink color. The Stokes shifts of **7**–**9** (870–1180 cm^−1^) are comparable to that of **6** (990 cm^−1^). The fluorescence quantum yields of compounds **7**–**9** (*Φ*_f_ 0.13–0.21) are smaller than that of **6** (0.38).

**FIGURE 4:**
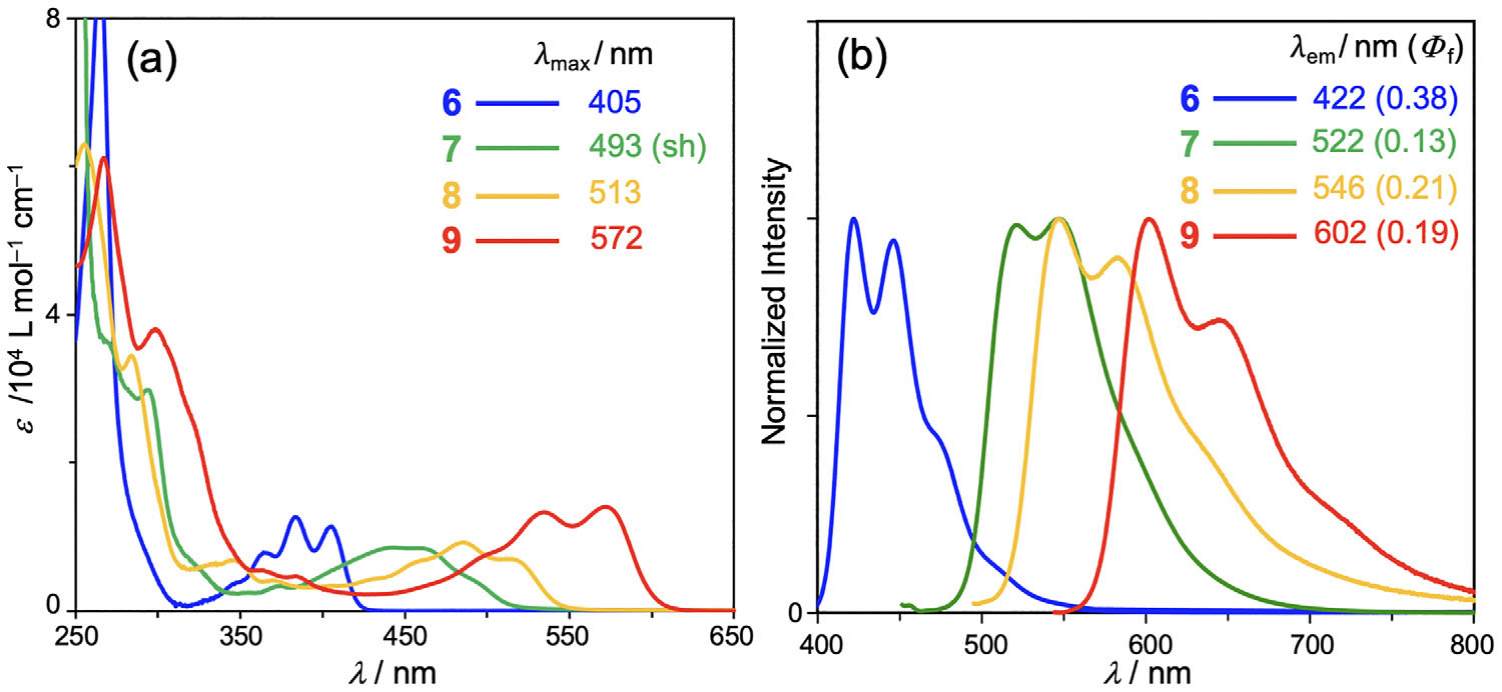
(a) UV–vis and (b) fluorescence spectra of compounds **6**–**9** in CHCl_3_ at room temperature. Concentration: UV–vis at 1.0 × 10^−5^ mol L^−1^, FL at 1.0 × 10^−6^ mol L^−1^.

### 2.4 Electrochemical Properties

The electrochemical behavior of compounds **6–9** was investigated by cyclic voltammetry (Figure 5 and Table 2). Compound **6** showed an oxidation wave at +0.68 V (*E*_1/2_ vs. Fc/Fc^+^) and no reduction wave was observed within the observed window. Compound **7** showed one reversible oxidation wave at +0.77 V and one reversible reduction wave at −1.95 V. Upon cyclization from **7** to **8**, the oxidation potential shifted to a less positive value of +0.58 V, while the reduction potential was not affected so much (−1.91 V). Two reduction waves were observed at −1.62 and −2.07 V for **9**, which has two five‐membered rings. The presence of two reduction waves in this region is common to other rubicene derivatives [27, 62, 63]. The first reduction potential (−1.62 V) of **9** became less negative compared to that of **8**. These data suggest that a seven‐membered ring stabilizes cationic species, while a five‐membered ring stabilizes anionic species. These tendencies are consistent with the general behavior of nonbenzenoid aromatic compounds. Consequently, the electrochemical band gaps decrease in the following order: **7** (2.72 eV), **8** (2.49 eV), and **9** (2.24 eV). The gap of **9** is even smaller than that of hexa‐*t*‐butylrubicene **10** (2.47 eV) [27], and comparable to that of fused dibenzo[*a*,*m*]rubicene **12** (2.18 eV) [61].

**FIGURE 5:**
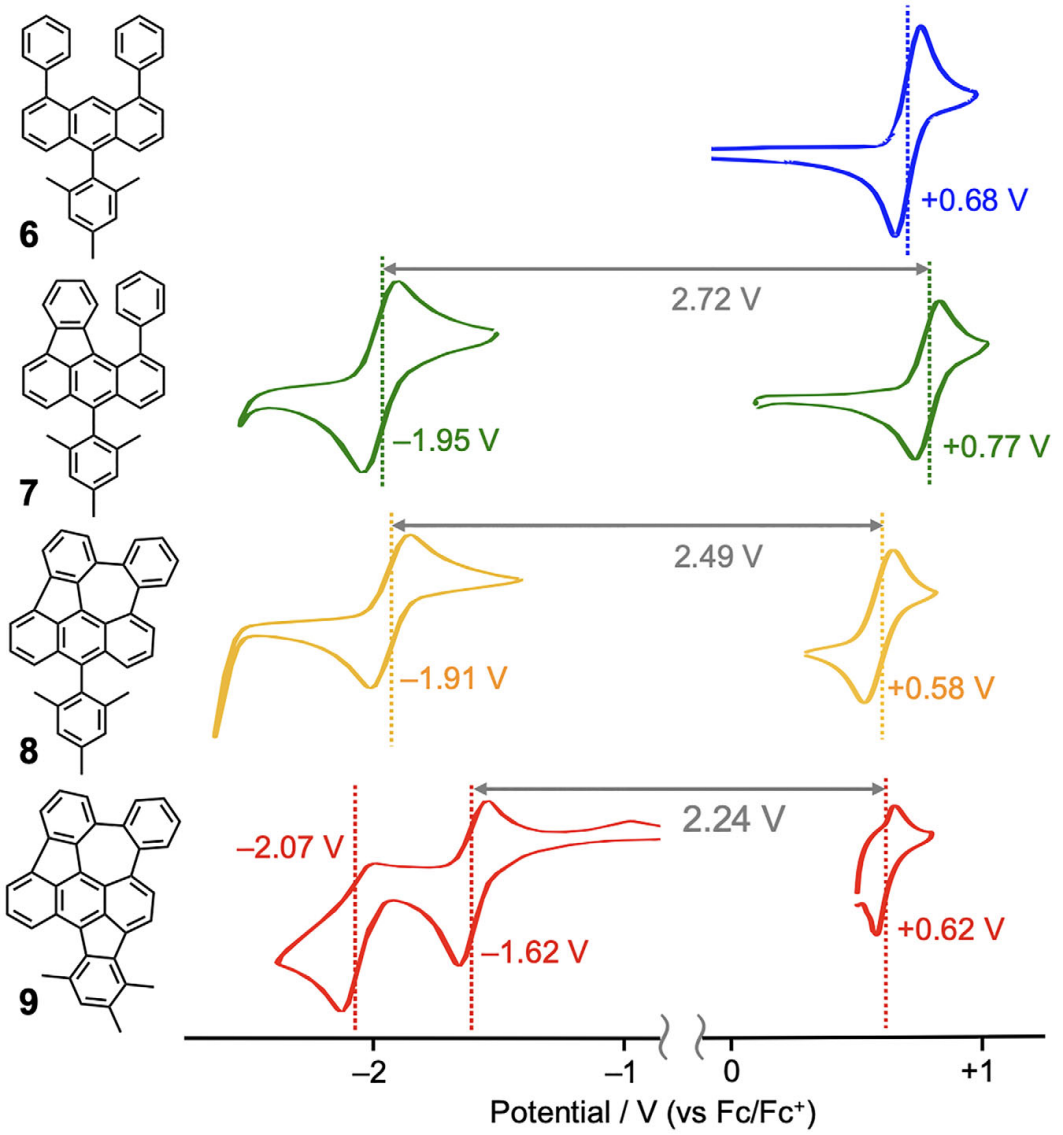
Cyclic voltammograms of **6**–**9** at room temperature. Half‐wave potentials and electrochemical gap are given. Conditions: glassy carbon working electrode, Pt counter electrode, Ag/AgNO_3_ reference electrode, and a 0.10 mol L^−1^ solution of Bu_4_NPF_6_ in CH_2_Cl_2_. Scan rate 100 mV s^−1^.

**TABLE 2:** Electrochemical, photophysical, and DFT calculation data of **6**–**9** and rubicene derivative **10**.

	CV				DFT	UV–vis	
Compounds	*E*_1/2_^ox1^/V ^a^	*E*_1/2_^red1^/V ^a^	*E*_1/2_^red2^/V ^a^	Δ*E*_g_^CV^/eV ^b^	Δ*E*_g_^DFT^/eV ^c^	Δ*E*_g_^opt1^/eV ^d^	Δ*E*_g_^opt2^/eV ^e^
**6**	+0.68	—	—	—	3.38	3.06	2.92
**7**	+0.77	−1.95	—	2.72	2.99	2.51	2.38
**8**	+0.58	−1.91	—	2.49	2.83	2.42	2.26
**9**	+0.62	−1.62	−2.07	2.24	2.54	2.17	2.09
**10** ^f^	+0.68	−1.79	−2.22	2.47	2.66	2.33	2.19

^a^ Half‐wave oxidation and reduction potentials. Measured conditions are provided in Figure 5.

^b^ Electrochemical band gap calculated from the first oxidation potential and the first reduction potential in the cyclic voltammograms.

^c^ Calculated HOMO–LUMO gap at the B3LYP‐D3/6‐31G(d,p) level.

^d^ Optical band gap calculated from the wavelength of the absorption maximum (shoulder for **7**).

^e^ Optical band gap calculated from the absorption onset at *ε* = 500.

^f^ Data of 2,4,6,9,11,13‐hexa‐*t*‐butylrubicene (**10**) [27].

### 2.5 DFT Calculations and Evaluation of Aromaticity

To obtain further structural and electronic information, we performed DFT calculations of **6**–**9**. The structures were optimized at the B3LYP‐D3/6‐31G(d,p) level (Figure S4). The calculated structures are similar to those of the X‐ray structures. Figure 6 shows the molecular orbital diagrams of the four compounds. Formation of the five‐membered ring remarkably lowers the LUMO level (**6**: −1.67 to **7**: −2.13 eV), and formation of the seven‐membered ring slightly elevates the HOMO level (**7**: −5.12 to **8**: −4.96 eV). The formation of an additional five‐membered ring further lowers the LUMO level of **9** to −2.39 eV. Consequently, the HOMO–LUMO gap narrows significantly from **6** to **9**, with a gap only 2.54 eV for **9** (cf. rubicene **2**: 2.74 eV and **10**: 2.66 eV) [23, 27, 64]. For **9**, the orbitals spread over the aromatic moieties at the HOMO level, while over the rubicene moiety at the LUMO level. Time‐dependent (TD)‐DFT calculations reasonably reproduced the observed UV–vis spectra (Figure S6). The calculated results for the HOMO–LUMO (S_0_→S_1_) excitations are shown in Figure 6. The calculated excitation wavelengths range from 408 nm for **6** to 559 nm for **9**, and their oscillator strengths *f* are very large.

**FIGURE 6:**
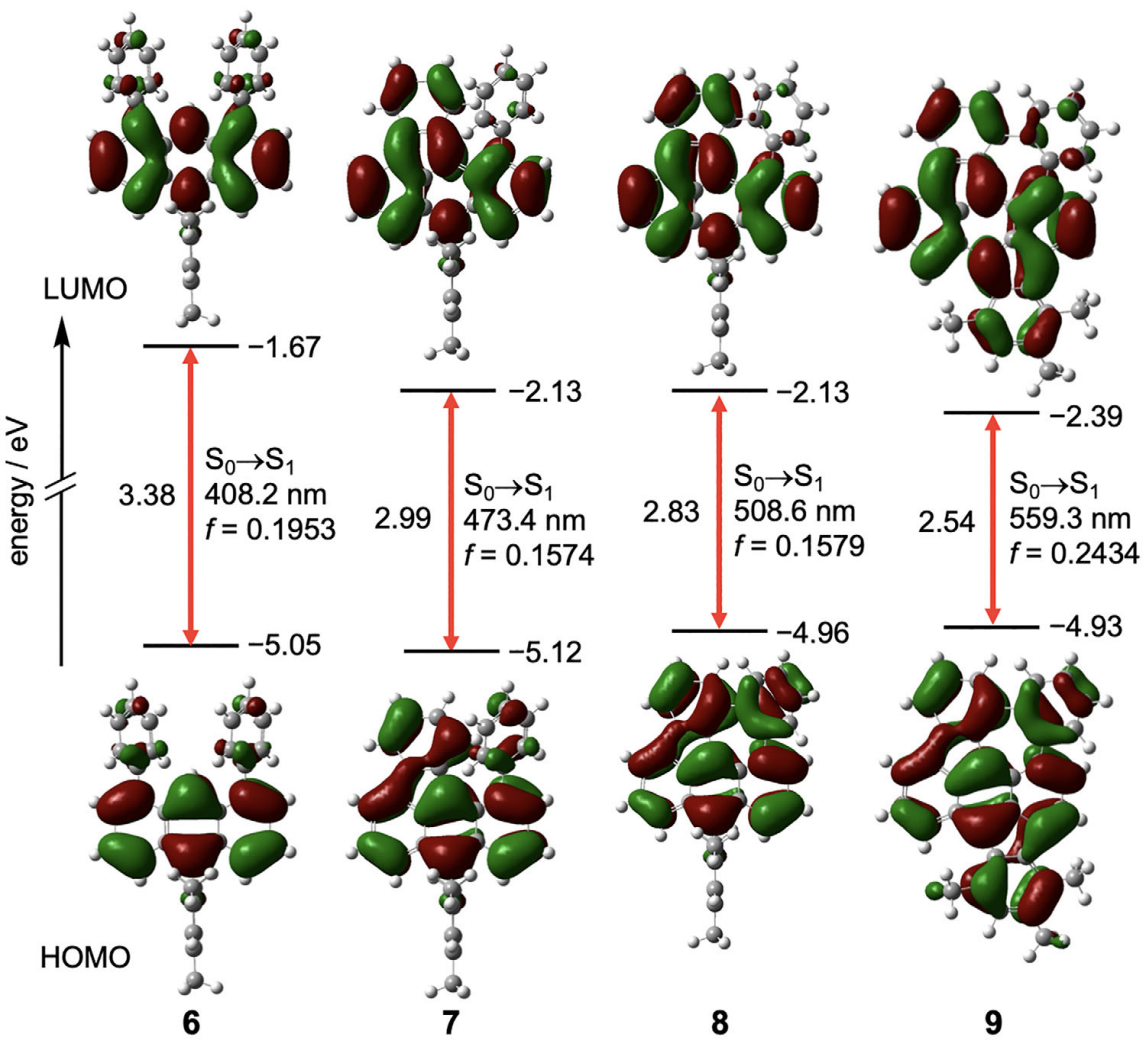
Molecular orbital diagrams and energies of HOMO and LUMO levels and selected TD‐DFT calculation data of compounds **6**–**9** at the B3LYP‐D3/6‐31G(d,p) level. Further information is provided in Figure S5.

To evaluate the aromaticity of the cyclized compounds, we calculated the nucleus‐independent chemical shift (NICS) value of each ring in the global minimum structures [65]. Because the aromatic frameworks of **7**–**9** are nonplanar, we adopted the NICS(1)_av_ value, an average of NICS(1) and NICS(−1), as shown in Figure 7a and Table S3 [66, 67]. The calculated values suggest that the six‐membered rings are aromatic and the five‐ and seven‐membered rings are nearly nonaromatic (nearly zero) in all compounds. These results are consistent with the harmonic oscillator model of aromaticity (HOMA) analysis of the X‐ray structure, as shown in Figure S2 [68, 69]. We also visualized the ring current effect of **7**–**9** using the anisotropy of current‐induced density (ACID) plot (Figure 7b) [70]. The isosurfaces of the π‐orbital contribution show that the clockwise arrows indicating the diatropic ring current are located on the anthracene moiety and the benzo groups almost independently. These results suggest that π‐conjugation does not extend across the five‐ and seven‐membered rings according to the magnetic indicator.

**FIGURE 7:**
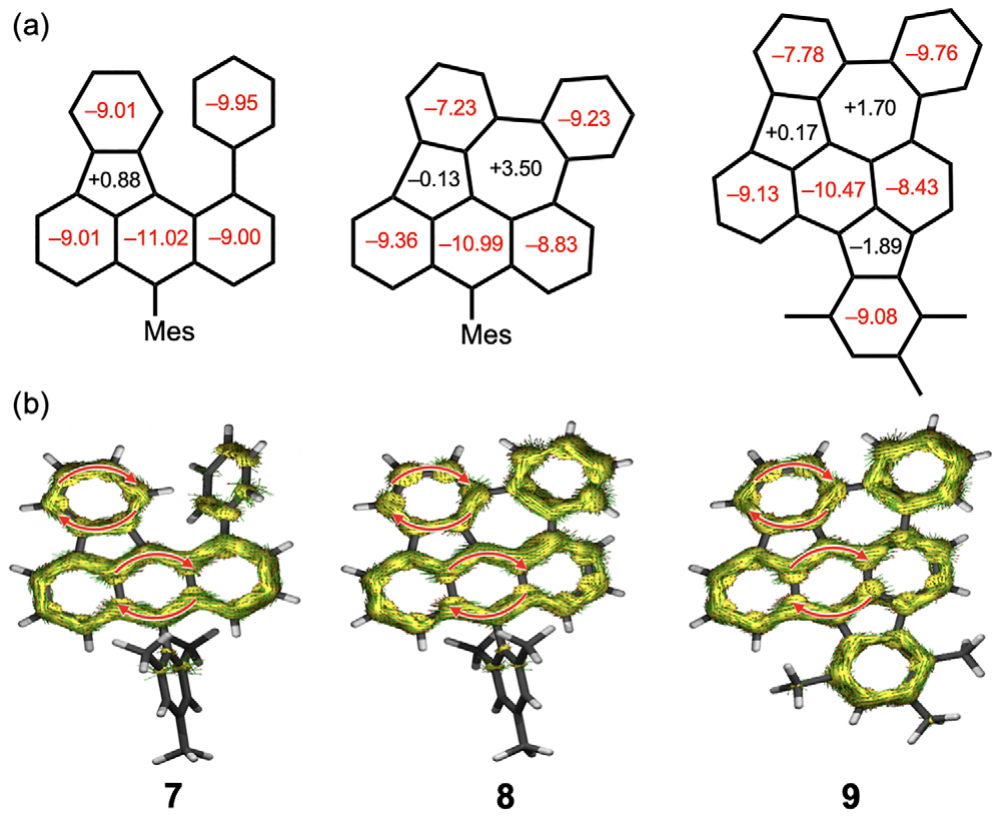
(a) NICS(1)_av_ values calculated at the GIAO‐B3LYP‐D3/6‐31G(d,p)//B3LYP‐D3/6‐31G(d,p) level and (b) ACID plots of contributions from π orbitals calculated at the CSGT‐B3LYP‐D3BJ/6–31G(d,p)// B3LYP‐D3BJ/6‐31G(d,p) level (isovalue: 0.050) of compounds **7**–**9**.

### 2.6 Reaction Mechanism

For the Scholl reactions, mechanisms involving radical cation or arenium ion intermediates were generally accepted based on the experimental and theoretical data [1, 2, 3, 4, 5, 6, 7, 8, 9, 10, 11, 52, 53, 54, 55, 56, 71]. As an alternative mechanism, the contribution of dication intermediates was also proposed for some reactions [13, 54]. In this case, the dication mechanism is unlikely because the reactions proceeded with chloranil (*E* ^red^ = –0.48 V vs Fc/Fc^+^), the reduction potential of which is insufficient to oxidize the reactant to a dication intermediate (cf. DDQ +0.14 V) [72]. To consider the possibility of the radical cation mechanism, we calculated radical cations of **6**–**8** at the UB3LYP‐D3/6‐31G(d,p) level (Figure 8a). The blue isosurfaces representing the Mulliken spin densities are primarily located on the anthracene moieties and no densities are found on the phenyl or mesityl moieties to be cyclized. For the reaction to proceed via the radical cation mechanism, it is preferable for both carbon atoms to possess some degree of spin density [13]. In addition, compounds **6** and **8** did not react with tris(4‐bromophenyl)ammoniumyl hexachloroantimonate (magic blue, *E* ^red^ = +0.70 V), which is a strong oxidant [72]. If their radical cations were involved as reactive intermediates, the reaction would occur to some extent under the conditions. These findings are against the radical cation mechanism. The use of strongly acidic TfOH plays a vital role for the reaction. Therefore, it is likely that the cyclization occurs via the arenium ion mechanism, as reported for the cyclization of several aromatic compounds [71, 73].

**FIGURE 8:**
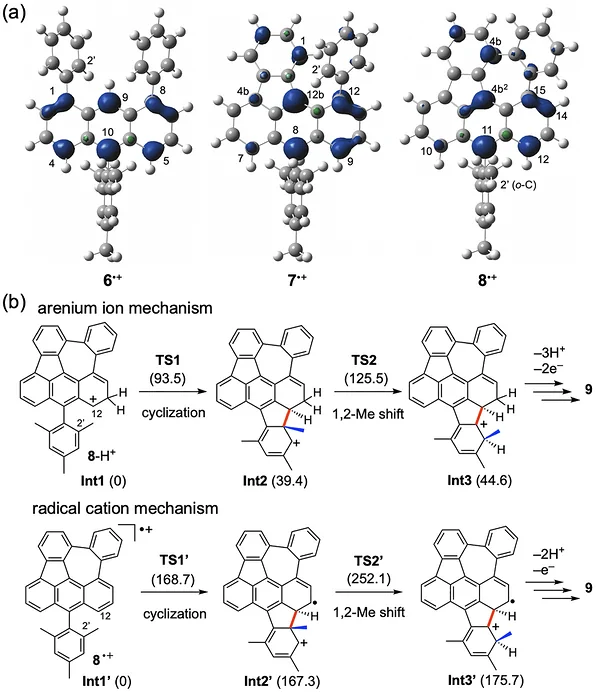
(a) Mulliken spin density diagrams of **6** ^•+^–**8** ^•+^ calculated at the UB3LYP‐D3/6‐31G(d,p) level (isovalue: 0.0040). (b) Plausible mechanisms of formation of **9** from arenium ion or radical cation of **8**. Relative free energies at 298 K to the initial intermediate calculated at the (U)B3LYP‐D3/6‐31G(d,p) level are given in parentheses in kJ mol^−1^.

The Me migration process is discussed further using additional computational and experimental data. Figure 8b shows plausible mechanisms for the formation of **9** from the arenium ion or radical cation of **8**. We assume that the cyclization occurs across the C12 atom and the C2’ atom in the mesityl group, and the Me group at the C2’ atom undergoes cationic 1,2‐shift to form another intermediate. The calculated free energies indicate that the radical cation mechanism is improbable because the intermediates and transition states are highly unstable. For the arenium ion mechanism, the two intermediates (**Int2** and **Int3**) are less stable by ca. 40 kJ mol^−1^ than the initial intermediate and the barriers are estimated to be 100 kJ mol^−1^. After the 1,2‐shift, some deprotonation and oxidation steps should result in the aromatization of the intermediate to form final rubicene product **9**. This type of reaction was not observed for the Scholl reaction of 9,10‐dimesitylanthracene under similar conditions (Supporting Information). Therefore, the Me migration is dependent on the substrate structure of 9‐mesitylanthracene derivatives.

## 3 Conclusions

The Scholl reactions of 10‐mesityl‐1,8‐diphenylanthracene produced the three cyclization products **7**–**9** via the formation of five‐ and seven‐membered rings. The aromatic frameworks of **8** and **9**, having a 5/7 ring system, are nonplanar due to steric hindrance between the peripheral atoms. As the cyclization proceeds, the *π*‐conjugation is extended as revealed by the optical and electrochemical data and the DFT calculations. The ACID and HOMA analyses revealed that the five‐ and seven‐membered rings in **7**–**9** are nearly nonaromatic. The rubicene formation from **8** to **9** can be explained by a mechanism involving the cyclization of an arenium ion intermediate followed by the 1,2‐methyl shift. These cyclization reactions provide a convenient method for constructing a 5/7 ring system from 1,8‐diphenylanthracene derivatives that lack functional groups. This type of ring structure is useful for designing highly fused aromatic hydrocarbons, such as fused azulenes and ring‐defective nanographenes. Further studies on the synthesis of fused azulenes, including those similar to circumazulene, as well as Scholl reactions of other phenyl‐substituted arenes such as 1,4,5,8‐tetraphenylanthracene and 1,8,10‐triphenylanthracene are underway.

## 4 Experimental Section

A typical procedure of the Scholl reaction and the physical and spectroscopic data of the cyclized products are described here. Other materials are provided in Supporting Information.

### 4.1 Scholl Reaction of Compound 6

A mixture of **6** [59] (50.0 mg, 0.111 mmol) and DDQ (50.6 mg, 0.223 mmol, 2.0 equiv.) in anhydrous CH_2_Cl_2_ (50 mL) was stirred at 0°C for 10 min under N_2_ atmosphere. After TfOH (2.50 mL, 28.2 mmol) was added to the solution, the mixture was stirred at 0°C for 2 h. The reaction mixture was poured into aq. NaHCO_3_ (50 mL). The organic materials were extracted with CH_2_Cl_2_ (25 mL × 3). The combined organic layer was dried over Na_2_SO_4_ and evaporated. The crude product was purified by chromatography on silica gel (hexane/CH_2_Cl_2_ 20:1) to give **7** (37.5 mg, 75%). A reaction of **6** (20.0 mg, 44.6 µmol) and DDQ (40.5 mg, 0.178 mmol, 4.0 equiv.) was similarly carried out. The crude product was purified by chromatography on silica gel (hexane/CH_2_Cl_2_ 20:1) to give **8** (3.7 mg, 19%) and **9** (11.8 mg, 60%) as less polar and polar compounds, respectively.

Compound **7**: Yellow solid; mp 127–130°C; *R*_f_ = 0.37 (hexane/CH_2_Cl_2_ 5:1); ^1^H NMR (500 MHz, CDCl_3_, 25°C): *δ* = 1.80 (s, 6H), 2.48 (s, 3H), 6.09 (d, *J* = 7.5 Hz, 1H), 6.77 (t, *J* = 7.5 Hz, 1H), 7.12 (s, 2H), 7.13 (t, *J* = 7.5 Hz, 1H), 7.39–7.45 (m, 5H), 7.52 (dd, *J* = 7.4, 6.5 Hz, 1H), 7.60 (dd, *J* = 7.4, 2.0 Hz, 2H), 7.64 (dd, *J* = 6.6, 1.1 Hz, 1H), 7.68 (dd, *J* = 8.6, 1.1 Hz, 1H), 7.86 (d, *J* = 7.5 Hz, 1H), 7.97 (d, *J* = 6.5 Hz, 1H); ^13^C NMR (125 MHz, CDCl_3_, 25°C): *δ* = 20.36, 21.27, 119.50, 120.19, 124.49, 125.43, 125.60, 126.69, 126.89, 127.07, 127.19, 127.24, 127.28, 127.91, 128.16, 128.84, 129.81, 130.97, 132.08, 132.44, 133.68, 134.36, 137.39, 137.65, 137.68, 138.42, 138.97, 139.57, 140.19, 144.50; HRMS (MALDI‐TOF): *m*/*z* calc. for C_35_H_26_: 446.2029 [*M*]^+^; found: 446.2017; UV–vis (CHCl_3_, 1.0 × 10^−5^ mol L^−1^, 25°C): *λ*_max_ (*ε*) = 271 (32500), 293 (29000), 355 (2410), 372 (3300), 442 (8490), 458 (8420), 493 nm (3930 L mol^−1^ cm^−1^); FL (CHCl_3_, 1.0 × 10^−6^ mol L^−1^, 25°C): *λ*_em_ = 522, 547 nm, *λ*_ex_ = 442 nm (*Φ*_f_ = 0.13 at 1.0 × 10^−5^ mol L^−1^).

Compound **8**: Orange solid; mp 202–205°C; *R*_f_ = 0.37 (hexane/CH_2_Cl_2_ 5:1); ^1^H NMR (500 MHz, CDCl_3_, 25°C): *δ* = 1.78 (s, 6H), 2.45 (s, 3H), 7.09 (s, 2H), 7.30 (dd, *J* = 8.8, 7.2 Hz, 1H), 7.36 (t, *J* = 7.8 Hz, 1H), 7.40 (d, *J* = 8.5 Hz, 1H), 7.41 (d, *J* = 8.8 Hz, 1H), 7.43–7.47 (m, 2H), 7.50 (dd, *J* = 8.5, 6.7 Hz, 1H), 7.67 (m, 1H), 7.69 (d, *J* = 7.2 Hz, 1H), 7.81 (d, *J* = 7.8 Hz, 1H), 7.87 (d, *J* = 7.8 Hz, 1H), 7.93 (m, 1H), 7.94 (d, *J* = 6.7 Hz, 1H); ^13^C NMR (125 MHz, CDCl_3_, 25°C): *δ* = 20.24, 21.24, 120.65, 121.40, 125.67, 125.86, 126.19, 126.81, 127.16, 127.49, 127.69, 128.16, 128.25, 128.91, 129.14, 129.46, 130.26, 132.66, 133.28, 133.48, 133.90, 135.53, 136.02, 136.45, 136.94, 137.31, 137.66, 138.23, 138.43, 140.19, 140.22, 140.91; HRMS (MALDI‐TOF): *m/z* calc. for C_35_H_24_: 444.1873 [*M*]^+^; found: 444.1865; UV–vis (CHCl_3_, 1.0 × 10^−5^ mol L^−1^, 25°C): *λ*_max_ (*ε*) = 255 (63000), 284 (34500), 335 (6520), 346 (6880), 369 (4120), 404 (3450), 485 (9260), 513 nm (7020 L mol^−1^ cm^−1^); FL (CHCl_3_, 1.0 × 10^−6^ mol L^−1^, 25°C): *λ*_em_ = 546, 583 nm, *λ*_ex_ = 485 nm (*Φ*_f_ = 0.21 at 1.0 × 10^−5^ mol L^−1^).

Compound **9**: Dark red solid; mp 236–240°C; *R*_f_ = 0.14 (hexane/CH_2_Cl_2_ 5:1); ^1^H NMR (500 MHz, CDCl_3_, 25°C): *δ* = 2.52 (s, 3H), 2.80 (s, 3H), 3.13 (s, 3H), 7.21 (s, 1H), 7.45 (t, *J* = 7.7 Hz, 1H), 7.53 (m, 2H), 7.73 (dd, *J* = 8.9, 6.8 Hz, 1H), 7.91 (d, *J* = 7.7 Hz, 1H), 8.00 (d, *J* = 7.7 Hz, 1H), 8.03 (d, *J* = 6.8 Hz, 1H), 8.07 (m, 1H), 8.10 (d, *J* = 7.4 Hz, 1H), 8.19 (m, 1H), 8.22 (d, *J* = 7.4 Hz, 1H), 8.83 (d, *J* = 8.9 Hz, 1H); ^13^C NMR (125 MHz, CDCl_3_, 25°C): *δ* = 16.39, 20.08, 25.30, 120.42, 121.29, 124.44, 125.72, 126.61, 127.23, 127.65, 128.00, 128.04, 128.12, 128.82, 129.11, 130.03, 130.58, 130.84, 131.70, 133.00, 133.89, 134.00, 134.10, 134.80, 134.84, 135.75, 136.51, 136.83, 137.11, 137.98, 138.08, 138.46, 138.64 (two aromatic signals were overlapped); HRMS (MALDI‐TOF): *m/z* calc. for C_35_H_22_: 442.1716 [*M*]^+^; found: 442.1703; UV–vis (CHCl_3_, 1.0 × 10^−5^ mol L^−1^, 25°C): *λ*_max_ (*ε*) = 267 (61100), 298 (38100), 364 (5520), 384 (4680), 534 (13300), 572 nm (14000 L mol^−1^ cm^−1^); FL (CHCl_3_, 1.0 × 10^−6^ mol L^−1^, 25°C): *λ*_em_ = 602, 644 nm, *λ*_ex_ = 534 nm (*Φ*_f_ = 0.19 at 1.0 × 10^−5^ mol L^−1^).

### 4.2 X‐Ray Crystallography

Deposition Numbers: 2492053 (for **7**), 2492054 (for **8**), and 2492055 (for **9**) contain the supplementary crystallographic data for this paper. These data are provided free of charge by the joint Cambridge Crystallographic Data Centre and Fachinformationszentrum Karlsruhe Access Structures service.

## Conflicts of Interest

The authors declare no conflicts of interest.

## Supporting information



Experimental details, X‐ray crystallographic analysis, UV–vis and fluorescence data, computational methods, NMR charts, and coordinates of the calculated structures. The authors have cited additional references within the Supporting Information [74, 75, 76, 77, 78, 79, 80, 81, 82].
**Supporting file 1**: chem71449‐sup‐0001‐SuppMat.pdf.

**Supporting file 2**: chem71449‐sup‐0002‐DataFile.zip.

## Data Availability

The data that supports the findings of this study are available in the Supporting Information of this article.
