# Supplementary material for: Cyclization of a 1,8‐Diphenylanthracene by Scholl Reactions to Form Five‐ and Seven‐Membered Ring Embedded Polycyclic Aromatic Hydrocarbons
Source: Chemistry. 2026 Jul 20;32(39):e71449. doi: 10.1002/chem.71449 (PMC13715047; doi:10.1002/chem.71449)
Supplement: Supplementary file 1 [file CHEM-32-e71449-s001.pdf]

Supporting Information

Table of contents

1. Experimental details **S1**
2. X-ray crystallographic analysis **S4**
3. UV/vis and fluorescence data **S7**
4. Computational methods **S8**
5. References **S17**
6. NMR charts **S19**
7. Coordinates of calculated structures **S25**

1. Experimental details

General

Melting points were measured with a Yanako Technical Science MP-500P and are uncorrected. NMR spectra were measured on a JEOL JNM-ECS400 spectrometer (^{1}H: 400 MHz, ^{13}C: 100 MHz) or a JNM-ECX500 spectrometer (^{1}H: 500 MHz, ^{13}C: 125 MHz). High-resolution MALDI-TOF mass spectra were measured on a Bruker UltrafleXtreme mass spectrometer using DCTB (*trans*-2-[3-(4-*t*-butylphenyl)-2-methyl-2-propenylidene]malononitrile) as a matrix and PEG-400, PEG-1000, or PEG-2000 (polyethylene glycol) as a calibrant. UV/Vis spectra were measured on a JASCO V-730 spectrometer with a 10 mm cell. Fluorescence spectra were measured on a JASCO FP-8550 spectrofluorometer with a 10 mm cell. Absolute fluorescence quantum yields were recorded on the JASCO FP-8550 spectrofluorometer with a JASCO ILF-135 120 mm integrating sphere with a 10 mm cell with the sample degassed by N_2 gas immediately before measurements. Cyclic voltammograms were recorded using an EC-Frontier ECstat-302. The data were collected in CH_2Cl_2 solution at room temperature with 0.10 mol L^{-1} of Bu_4NPF_6 as electrolyte, employing a grassy carbon rod as working electrode, a Pt wire as counter electrode against an $Ag/AgNO_3$ in a Luggin capillary. The scan rate was 100 mV s^{-1}. All potentials were referenced to Fc/Fc^+ under identical conditions.

10-Mesityl-1,8-diphenylanthracene (6)[S1]

A solution of 1,8-dichloro-10-mesitylanthracene (100 mg, 0.274 mmol),[S2] $PhB(OH)_2$ (100 mg, 0.821 mmol), K_2CO_3 (151 mg, 1,09 mmol), $Pd_2(dba)_3$ (2.51 mg, 2.74 μmol), and XPhos (5.22 mg, 10.9 μmol) in a degassed mixture of toluene (1.0 mL), 1,4-dioxane (0.50 mL), and H_2O (0.50 mL) was stirred at 100 °C for 24 h under N_2 atmosphere. After it was cooled to room temperature, H_2O (15 mL) was added. The organic materials were extracted with CH_2Cl_2 (15 mL × 3). The combined organic layer was dried over Na_2SO_4 and evaporated. The crude product was purified by chromatography on silica gel with CH_2Cl_2 to give the desired product as a yellow solid. Yield 113 mg (92%); R_f = 0.37 (hexane/CH_2Cl_2 5:1); ^{1}H NMR (500 MHz, $CDCl_3$): δ = 8.65 (s, 1H), 7.52 (d, J = 7.2 Hz, 4H), 7.48 (d, J = 7.3 Hz, 2H), 7.43–7.35 (m, 10H), 7.12 (s, 2H), 2.48 (s, 3H), 1.79 (s, 6H); UV/Vis ($CHCl_3$, 1.0 × 10^{-5} mol L^{-1}, 25 °C): λ_{max} (ε) = 265 (88100), 364 (7900), 384 (12600), 405 nm (11400 L mol^{-1} cm^{-1}); FL ($CHCl_3$, 1.0 × 10^{-6} mol L^{-1}, 25 °C): λ_{em} = 422, 446 nm, λ_{ex} = 384 nm (Φ_f = 0.38 at 1.0 × 10^{-5} mol L^{-1}).

Scholl reaction of 6 (DDQ 2 equiv.)

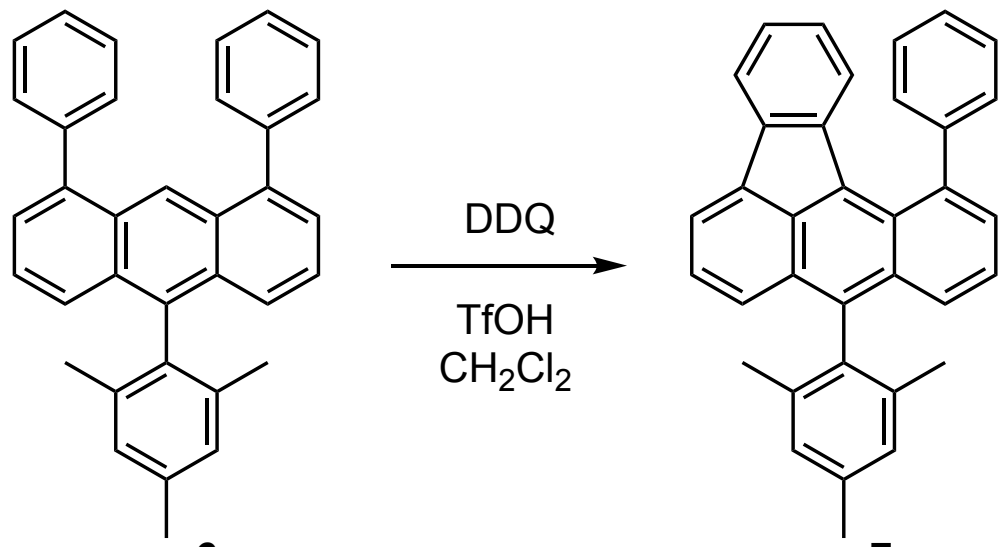

6 7

The experimental procedure and the physical and spectroscopic data of **7** are provided in Experimental Section.

Scholl reaction of 6 (DDQ 4 equiv.)

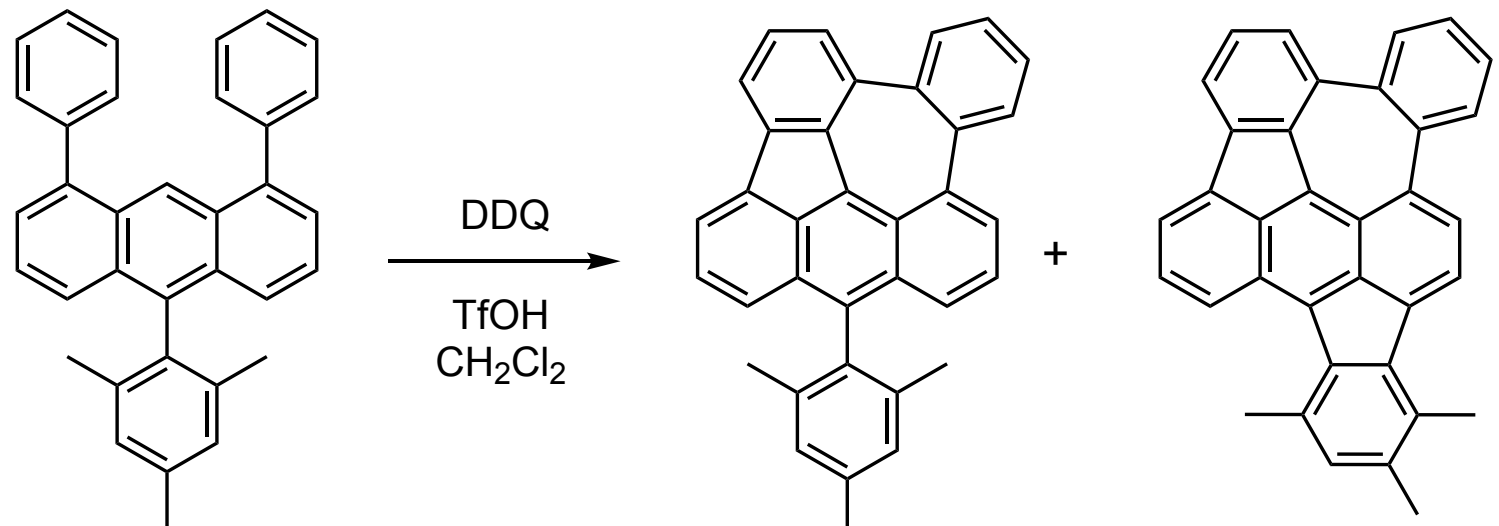

6 8 9

The experimental procedure and the physical and spectroscopic data of **8** and **9** are provided in Experimental Section.

Scholl reaction of 6 (other conditions)

The reactions of **6** with variable amounts of DDQ or chloranil were carried out in a similar manner. The NMR yields were determined by measuring signal intensities relative to that of CH_3NO_2 as an internal reference. The results are listed in Table 1. Compound **6** were also treated under the following conditions: DDQ (4.0 equiv.)/MsOH (methanesulfonic acid), DDQ (4.0 equiv.) in the absence of TfOH, TfOH only, or tris(4-bromophenyl)ammoniumyl hexachloroantimonate (magic blue, 2.0 equiv.). In every case, the starting material was recovered without reaction.

Scholl reaction of 7

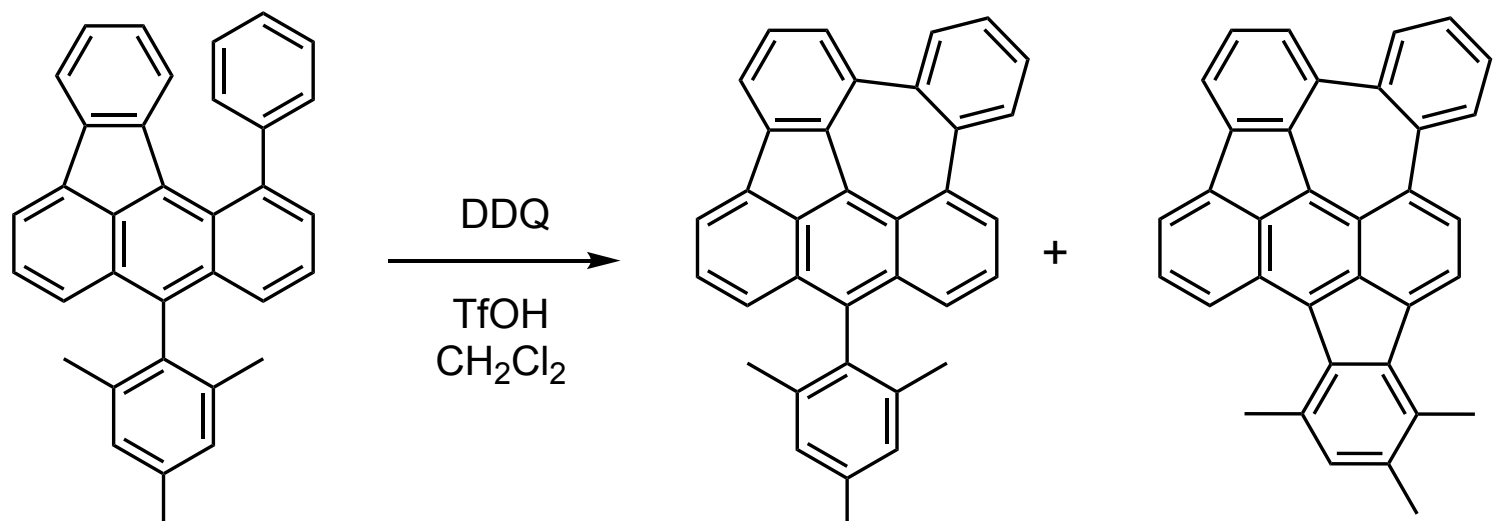

7 8 9

A mixture of **7** (20.0 mg, 44.8 μmol) and DDQ (30.5 mg, 0.134 mmol, 3.0 equiv.) in anhydrous CH_2Cl_2 (20 mL) was stirred at 0 °C for 10 min under N_2 atmosphere. After TfOH (1.0 mL, 11 mmol) was added to the solution, the mixture was stirred at 0 °C for 2 h. The reaction mixture was poured into aq. $NaHCO_3$ (20 mL). The organic materials were extracted with CH_2Cl_2 (20 mL × 3). The combined organic layer

was dried over Na_2SO_4 and evaporated. The crude product was purified by chromatography on silica gel with hexane/CH_2Cl_2 (20:1) as eluent to give the following products. Compound **8**: NMR yield 21%. Isolated yield 4.0 mg (20%). Compound **9**: NMR yield 64%. Isolated yield 6.9 mg (35%).

Scholl reactions of other substituted anthracenes

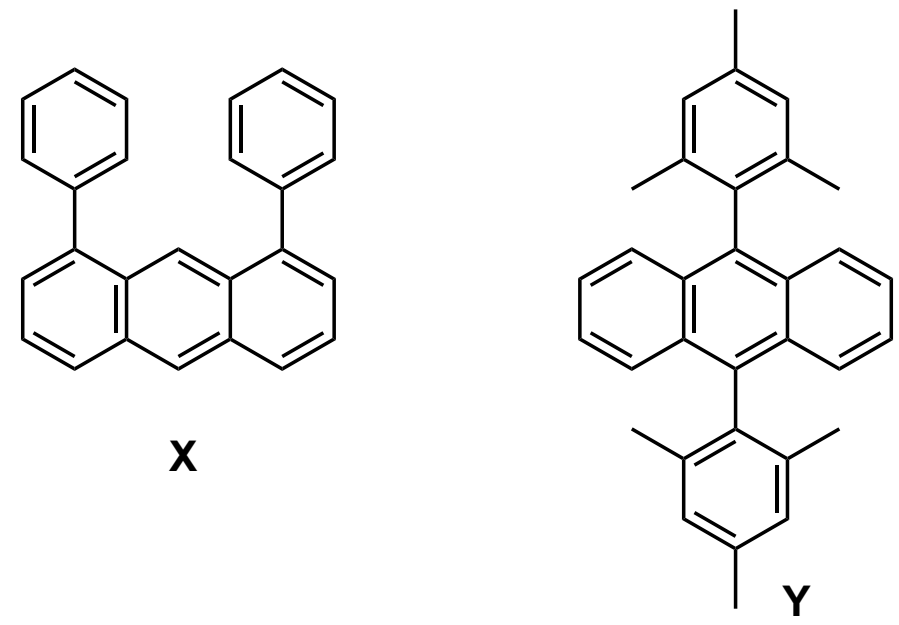

1,8-Diphenylanthracene (X): A mixture of **X**[S3] (10.0 mg, 30.3 µmol) and DDQ (20.6 mg, 90.8 µmol, 3.0 equiv.) in anhydrous CH_2Cl_2 (10 mL) was stirred at 0 °C for 10 min under N_2 atmosphere. After TfOH (0.5 mL, 5.6 mmol) was added to the solution, the mixture was stirred at 0 °C for 10 min. The reaction mixture was poured into aq. $NaHCO_3$ (10 mL). The organic materials were extracted with CH_2Cl_2 (10 mL × 3). The combined organic layer was dried over Na_2SO_4 and evaporated to give an insoluble solid as product. The product analysis by the ^{1}H NMR spectroscopy was unsuccessful.
9,10-Dimesitylanthracene (Y): A mixture of **Y**[S4] (10.0 mg, 24.1 µmol) and DDQ (16.4 mg, 72.4 µmol, 3.0 equiv.) in anhydrous CH_2Cl_2 (10 mL) was stirred at 0 °C for 10 min under N_2 atmosphere. After TfOH (0.5 mL, 5.6 mmol) was added to the solution, the mixture was stirred at 0 °C for 2 h. The reaction mixture was poured into aq. $NaHCO_3$ (10 mL). The organic materials were extracted with CH_2Cl_2 (10 mL × 3). The combined organic layer was dried over Na_2SO_4 and evaporated. The starting material was recovered without reaction.

2. X-ray crystallographic analysis

Diffraction data of compounds **7**, **8**, and **9** were collected at 93 K on a Pilatus S6M diffractometer with synchrotron radiation (λ = 0.750 Å) equipped in a beamline BL-5A at the High Energy Accelerator Research Organization (KEK), Japan, and processed with XDS. Structures were solved by the intrinsic phasing method (SHELXT 2018)[S5] and refined by non-linear least square method (SHELXL 2018)[S6-S8] using Olex2.1.5 (2023)[S9] as a graphical user interface. Non-hydrogen atoms were refined anisotropically. Hydrogen atoms were refined with the riding model.

Compound **7**:

A yellow single crystal was prepared by crystallization from toluene and MeOH. Crystallographic data: Formula $C_{35}H_{26}\cdot 0.25(C_7H_8)$, M = 469.59, monoclinic, $P2_1/n$, a = 20.8780(11), b = 9.9460(19), c = 25.1690(19) Å, β = 103.376(5)°, V = 5084.6(11) Å^3, Z = 8, D_c = 1.227 g cm^{-3}, θ_{max} = 71.106°, μ(synchrotron) = 0.077 mm^{-1}, number of unique reflections 18675, R_1 = 0.0545 [$I > 2.0\sigma(I)$], wR_2 = 0.1541 (all data), GOF = 1.085, CCDC 2492053.

Compound **8**:

An orange single crystal was prepared by crystallization from CH_2Cl_2 and MeOH. Crystallographic data: Formula $C_{70}H_{48}$, M = 889.08, orthorhombic, $Pbca$, a = 7.119(7), b = 24.046(6), c = 26.053(6) Å, V = 4460(5) Å^3, Z = 4, D_c = 1.324 g cm^{-3}, θ_{max} = 71.112°, μ(synchrotron) = 0.083 mm^{-1}, number of unique reflections 7179, R_1 = 0.0560 [$I > 2.0\sigma(I)$], wR_2 = 0.1555 (all data), GOF = 1.030, CCDC 2492054.

Compound **9**:

A dark red single crystal was prepared by crystallization from $CHCl_3$ and MeOH. Crystallographic data: Formula $C_{35}H_{22}$, M = 442.52, monoclinic, $C2/c$, a = 42.690(8), b = 3.818(2), c = 27.105(4) Å, β = 112.484(2)°, V = 4082(3) Å^3, Z = 8, D_c = 1.440 g cm^{-3}, θ_{max} = 71.068°, μ(synchrotron) = 0.091 mm^{-1}, number of unique reflections 6868, R_1 = 0.0469 [$I > 2.0\sigma(I)$], wR_2 = 0.1298 (all data), GOF = 1.044, CCDC 2492055.

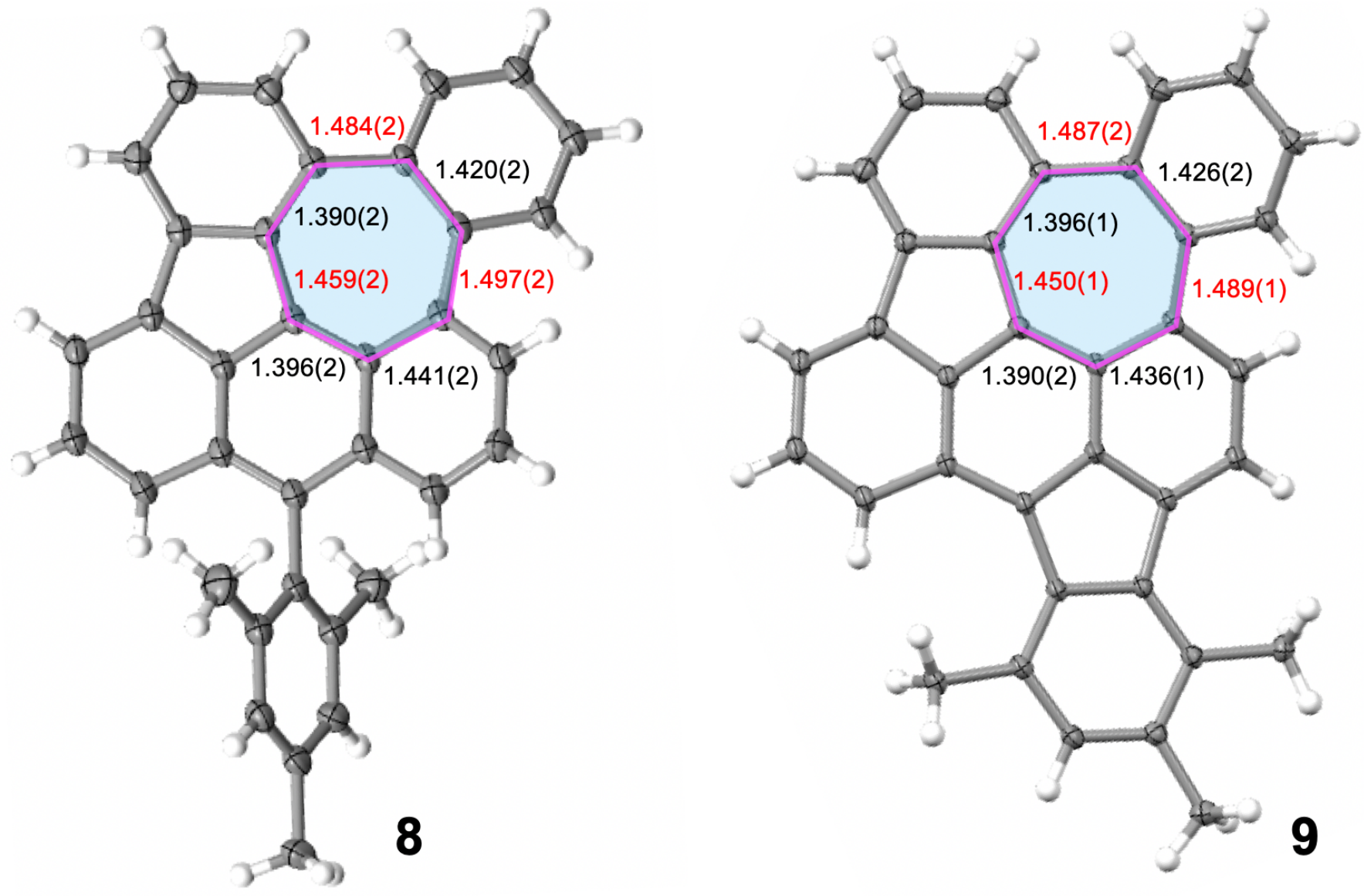

Figure S1. Bond distances (Å) of C–C bonds around the seven-membered ring in **8** and **9** with standard deviations. Distances of long bonds are highlighted in red.

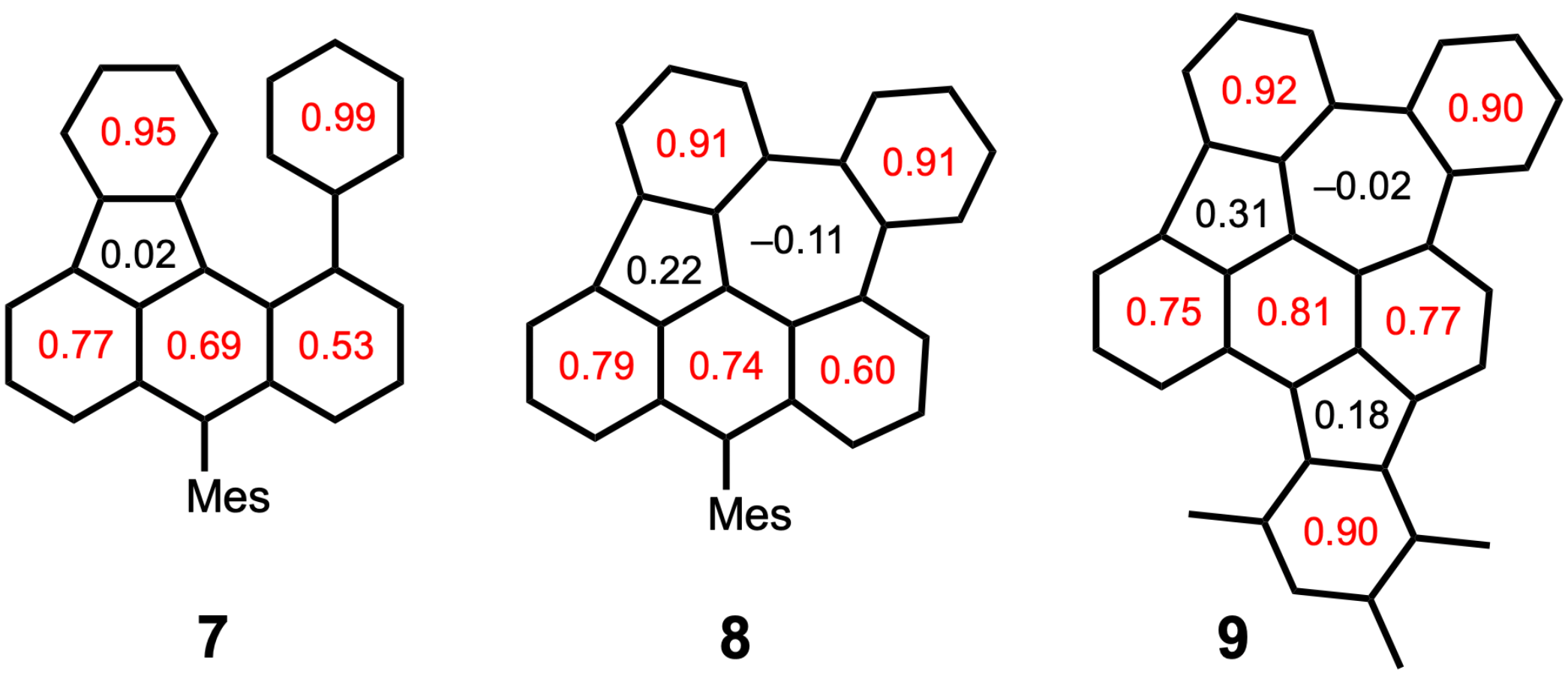

Figure S2. Harmonic oscillator model of aromaticity (HOMA) analysis[S10,S11] of the X-ray structures of **7**–**9**. HOMA values are defined by the following equation: $\mathrm{HOMA} = 1 - (\alpha/n)\sum_{i=1}^{n}(R_{\mathrm{opt}} - R_i)^2$, where α = 257.7 for C–C bond and R_{opt} = 1.388 Å were used, where n is the number of bonds in each ring and R_i the bond length of the i-th bond. Values larger than 0.5 are highlighted in red.

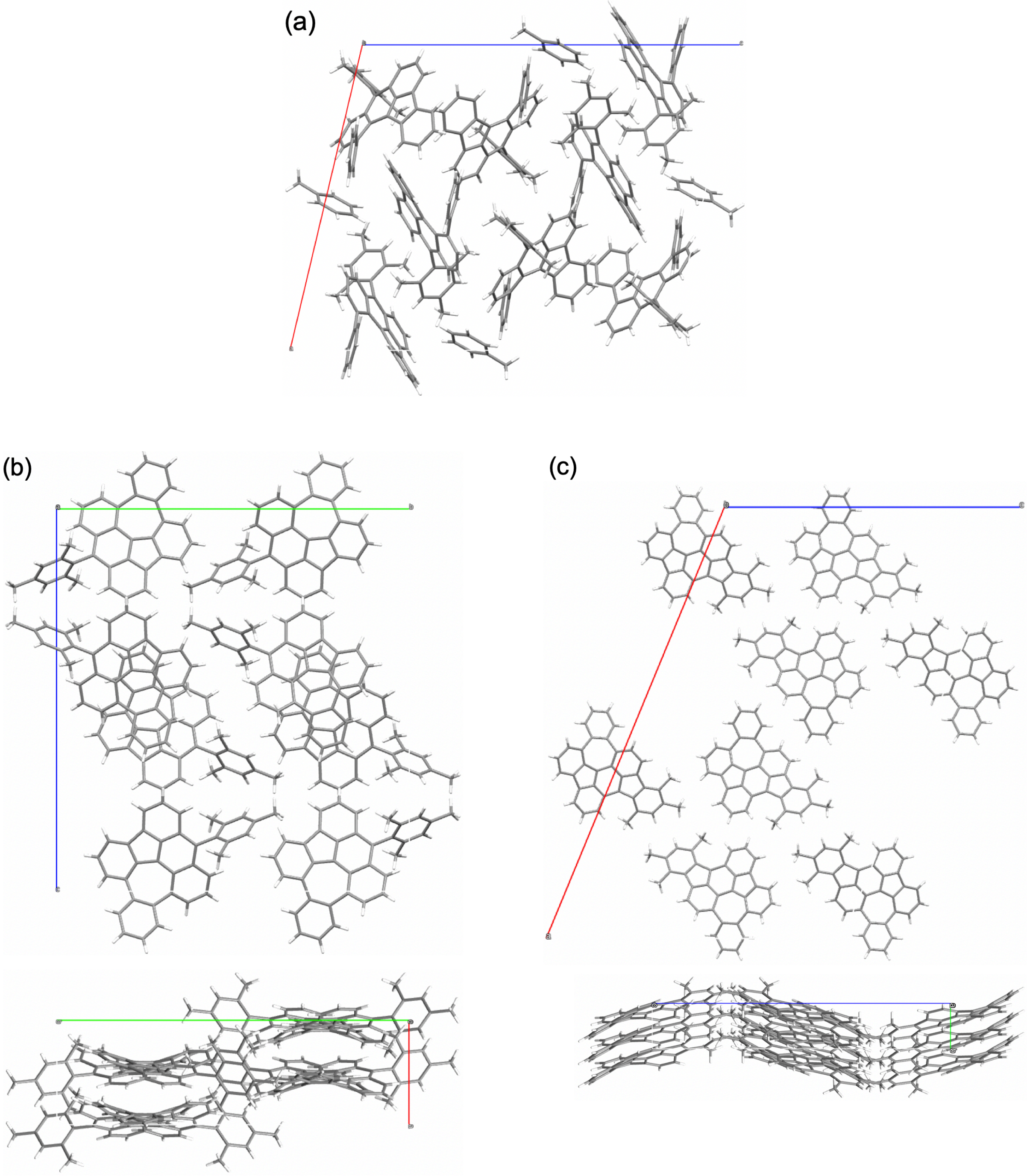

Figure S3. Packing diagrams of X-ray structures of (a) **7**, (b) **8**, and (c) **9**.

3. UV/vis and fluorescence data

Table S1. Spectroscopic data of compounds **6–9** and rubicene derivatives **10–12** in $CHCl_3$ at room temperature.[a]

	UV-vis λ_{max}/nm (ε/L mol^{-1} cm^{-1})	FL λ_{em}/nm	Φ_f [b]	Stokes shift /cm^{-1}
**6**	405 (11400)	422	0.38	990
**7**	493 (sh, 3930)	522	0.13	1130
**8**	513 (7020)	546	0.21	1180
**9**	572 (14000)	602	0.19	870
**10** [c]	533 (sh, 7600)	572	0.11	1280
**11** [d]	ca. 490 (sh)	ca. 530	0.19	ca. 1500
**12** [e]	529	559	0.12	1010

[a] Concentration: UV/vis and FL quantum yield at 1.0×10^{-5} mol L^{-1}, FL at 1.0×10^{-6} mol L^{-1}, unless otherwise noted. Structures of **10–12** are given below. sh: shoulder peak.
[b] Fluorescence quantum yield.
[c] Measured at 1.0×10^{-5} mol L^{-1}.[S12]
[d] Measured in CH_2Cl_2 at 5×10^{-6} mol L^{-1}.[S13]
[e] Measured in CH_2Cl_2 at 1×10^{-5} mol L^{-1}.[S14]

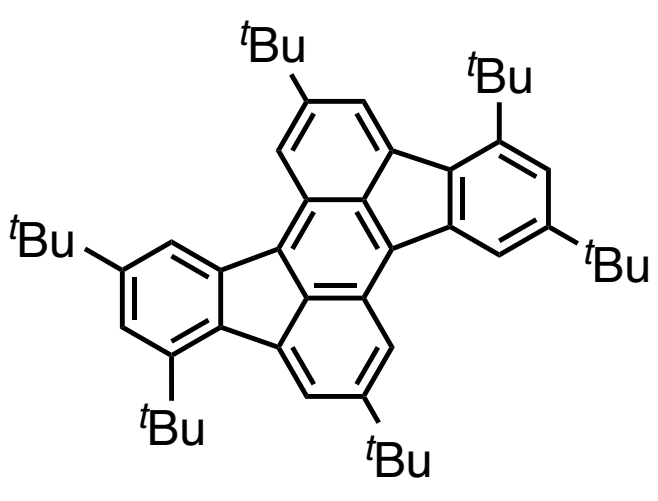

10
2,4,6,9,11,13-hexa-*t*-butylrubicene

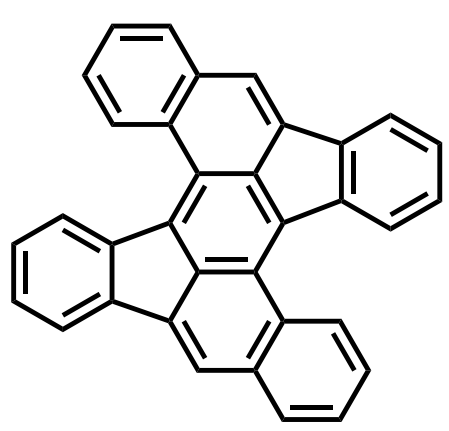

11
dibenzo[*a*,*m*]rubicene

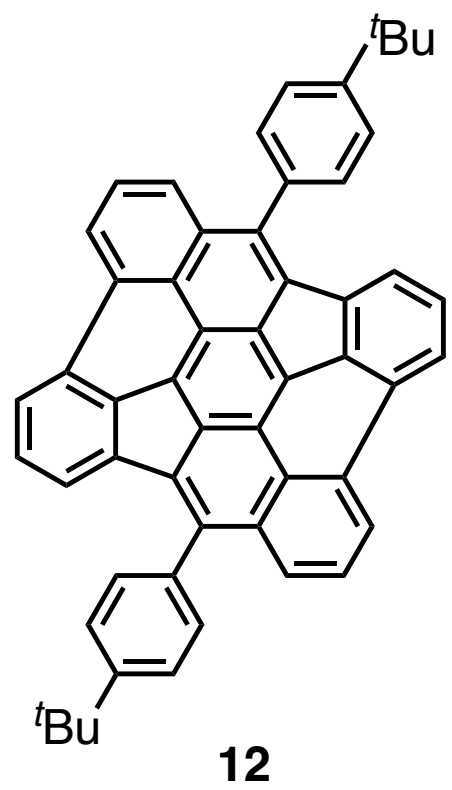

12
fused dibenzo[*a*,*m*]rubicene

4. Computational methods

Calculations were carried out with Gaussian 16 program[S15] on a windows computer. The structures were optimized by the hybrid DFT method at the B3LYP-D3/6-31G(d,p) level unless otherwise noted. The frequency analysis was carried out for each optimized structure, giving no one imaginary wavenumber for energy minimum structures and transition structures, respectively. The optimized structures of **6–9** were shown in Figure S4. The energy levels of frontier orbitals of **7–9** are shown in Figure S5. The calculations of excited states were carried out by TD-DFT method at the B3LYP-D3/6-31G(d,p) level with the global minimum structures. The number of excited states (NSTATE) was 30. The calculated UV/Vis spectra and data are shown in Figure S6 and Table S2, respectively.

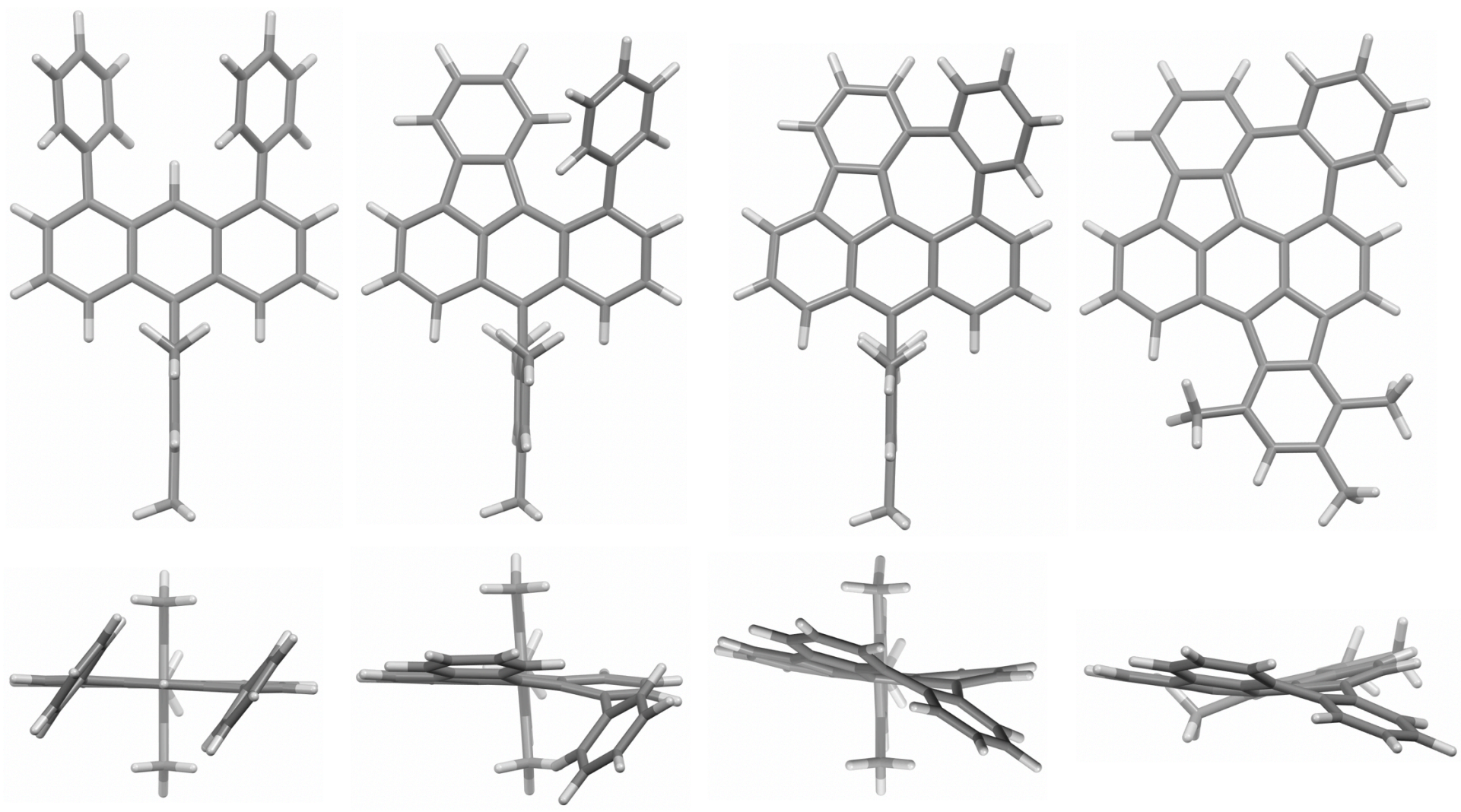

6 **7** **8** **9**

Figure S4. Two views of optimized structures of **6–9** calculated at the B3LYP-D3/6-31G(d,p) level.

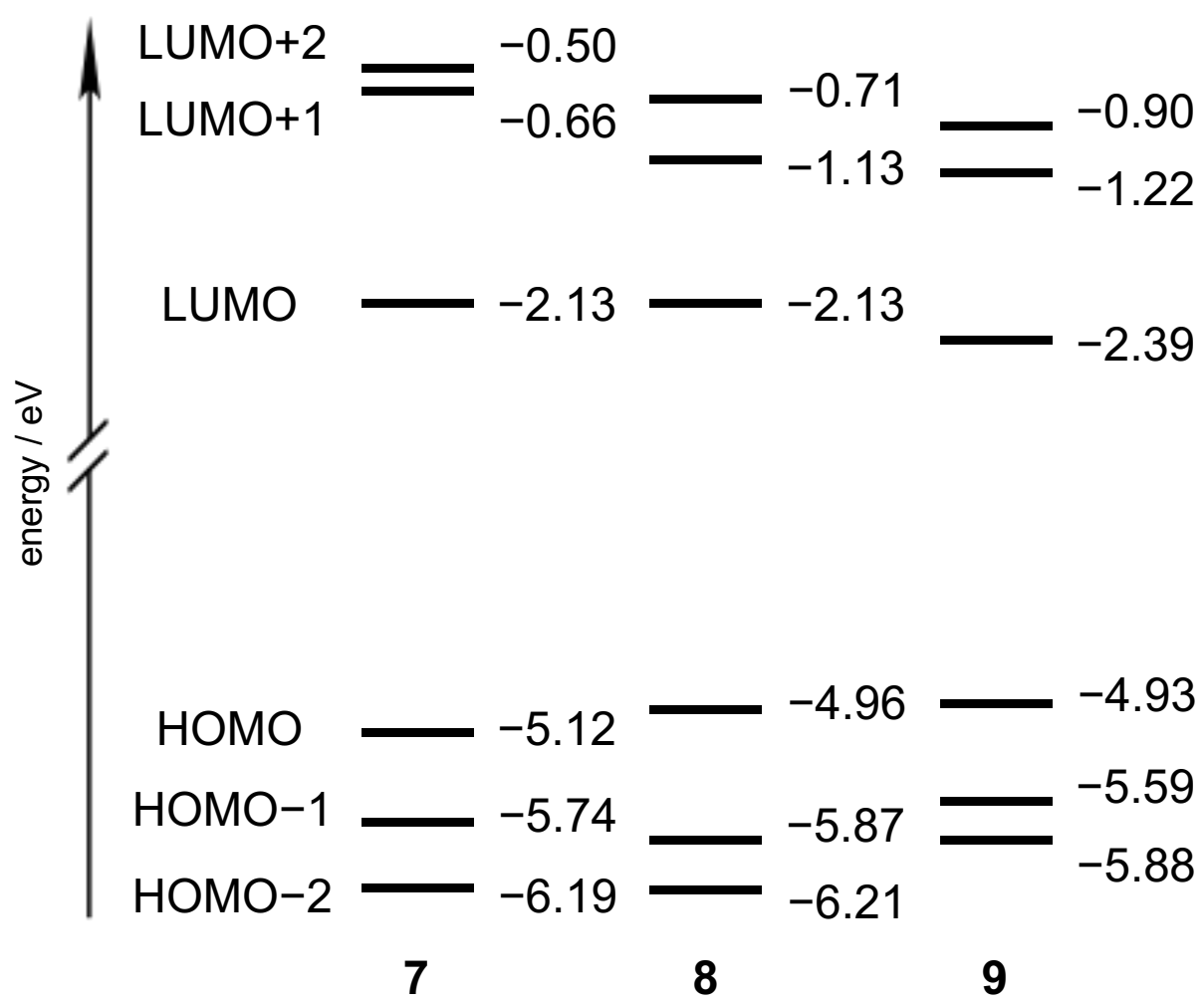

Figure S5. Energy levels of frontier orbitals of **7–9** calculated at the B3LYP-D3/6-31G(d,p) level.

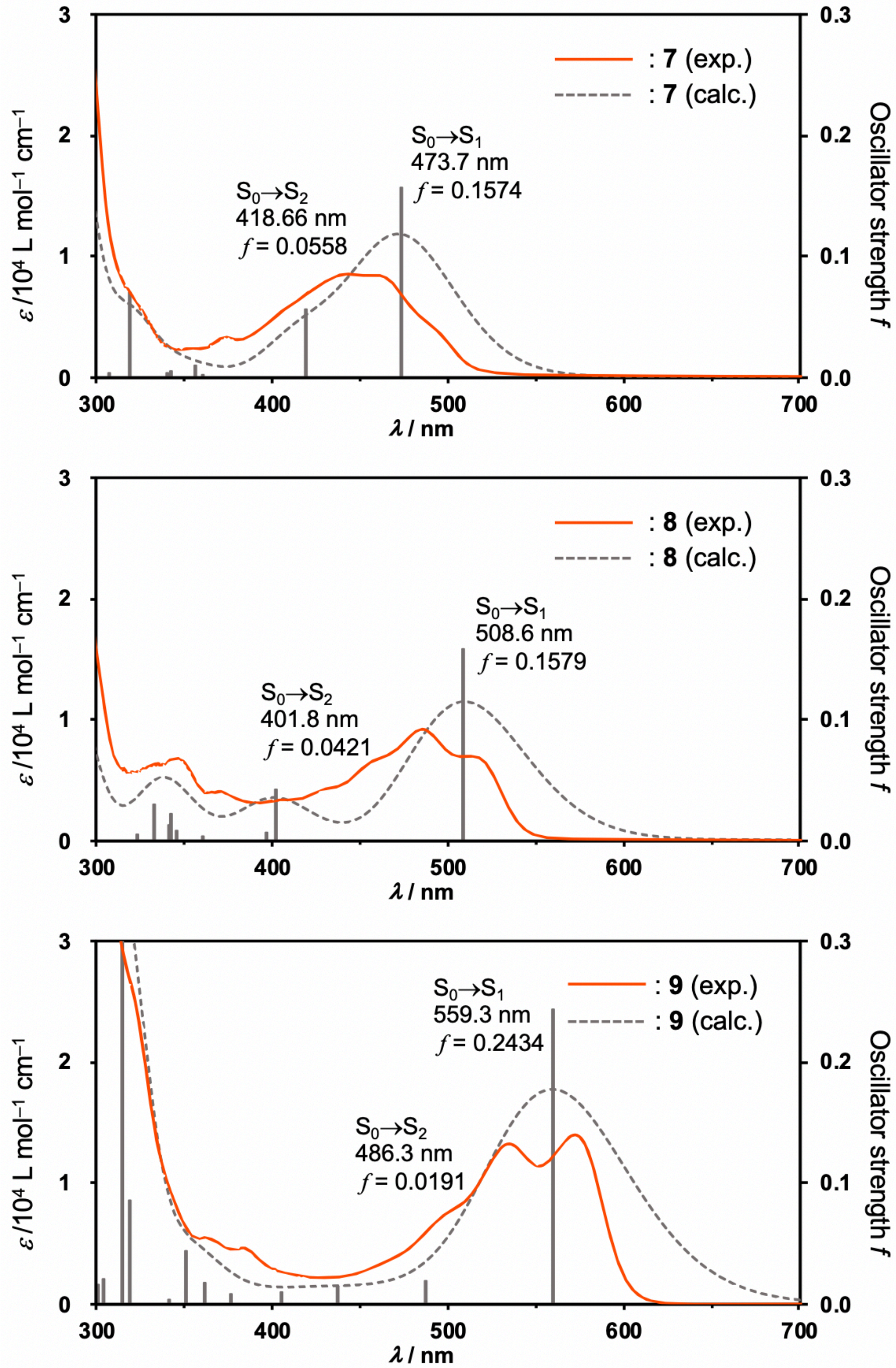

Figure S6. Observed and calculated UV/Vis spectra of **7**–**9**. Observed UV/Vis spectra were measured in $CHCl_3$. TD-DFT calculation were calculated at the TD-B3LYP-D3/6-31G(d,p)//B3LYP-D3/6-31G(d,p) level. In the UV/Vis spectra, gray bars show oscillator strengths. The calculated spectra were simulated at full width at half maximum 1500 cm^{-1}.

Table S2. Selected TD-DFT data for the absorption of **7–9** calculated at the TD-B3LYP-D3/6-31G(d,p)//B3LYP-D3/6-31G(d,p) level.

Compound **7**

excited state	Energy/eV	λ/nm [a]	f [b]	transition contribution [c]
1	2.6190	473.40	0.1574	H→L (90.2%)
2	2.9615	418.66	0.0558	H–1→L (89.3%)
3	3.4380	360.63	0.0014	H–3→L (60.9%) H–2→L (37.8%)
4	3.4916	355.09	0.0093	H–2→L (57.7%) H–3→L (37.7%)
5	3.6257	341.96	0.0051	H–4→L (59.3%) H–5→L (19.1%) H→L+2 (14.3%)
6	3.6516	339.53	0.0030	H–4→L (40.1%) H–5→L (34.9%) H→L+2 (16.2%)
7	3.8911	318.63	0.0706	H→L+1 (82.5%) H→L+2 (10.1%)
8	4.0390	306.97	0.0028	H–6→L (97.3%)
9	4.3013	288.25	0.1810	H–7→L (37.9%) H→L+2 (22.6%) H–5→L (10.7%)
10	4.3558	284.64	0.1168	H–7→L (52.1%) H→L+2 (19.6%) H→L+3 (10.8%)

Compound **8**

excited state	Energy/eV	λ/nm [a]	f [b]	transition contribution [c]
1	2.4378	508.59	0.1579	H→L (94.9%)
2	3.0857	401.80	0.0421	H–1→L (90.6%)
3	3.1238	396.91	0.0060	H→L+1 (95.9%)
4	3.4419	360.22	0.0029	H–2→L (99.0%)
5	3.5918	345.18	0.0079	H–3→L (63.3%) H→L+2 (35.2%)
6	3.6171	342.77	0.0218	H–4→L (30.3%) H–3→L (27.8%) H→L+2 (19.4%) H–6→L (13.3%)
7	3.6384	340.76	0.0128	H–4→L (65.6%) H→L+2 (16.9%) H–6→L (10.3%)
8	3.7304	332.36	0.0292	H→L+2 (36.9%) H–6→L (29.3%) H→L+3 (26.1%)
9	3.8479	322.21	0.0057	H–5→L (92.5%)
10	4.1686	297.42	0.0317	H–1→L+1 (84.1%)

(Table S2. *continued*)

Compound **9**

excited state	Energy/eV	λ/nm [a]	f [b]	transition contribution [c]
1	2.2168	559.28	0.2434	H→L (94.8%)
2	2.5497	486.27	0.0191	H–1→L (97.1%)
3	2.8406	436.47	0.0136	H–2→L (94.1%)
4	3.0653	404.47	0.0102	H→L+1 (97.5%)
5	3.2944	376.35	0.0075	H–3→L (92.9%)
6	3.4334	361.11	0.0168	H→L+2 (82.0%)
7	3.5407	350.17	0.0441	H–4→L (59.8%) H→L+3 (15.1%) H–5→L (10.3%)
8	3.6361	340.98	0.0027	H–5→L (64.2%) H–4→L (25.3%)
9	3.8896	318.75	0.0854	H–6→L (66.1%) H–1→L+1 (25.6%)
10	3.9465	314.16	0.3461	H–1→L+1 (55.4%) H–6→L (23.6%)

[a] Excitation wavelength.
[b] Oscillator strength.
[c] H: HOMO, L: LUMO. Only transitions of >10% contributions are listed.

NICS

The nucleus-independent chemical shift (NICS) values were calculated at the GIAO-B3LYP-D3/6-31G(d,p), where the ghost atoms were located at the distance of +1, 0, and –1 Å from the least-squares plane defined by the rings as shown in Figure S7.[S16–S18] The calculated values are listed in Table S3.

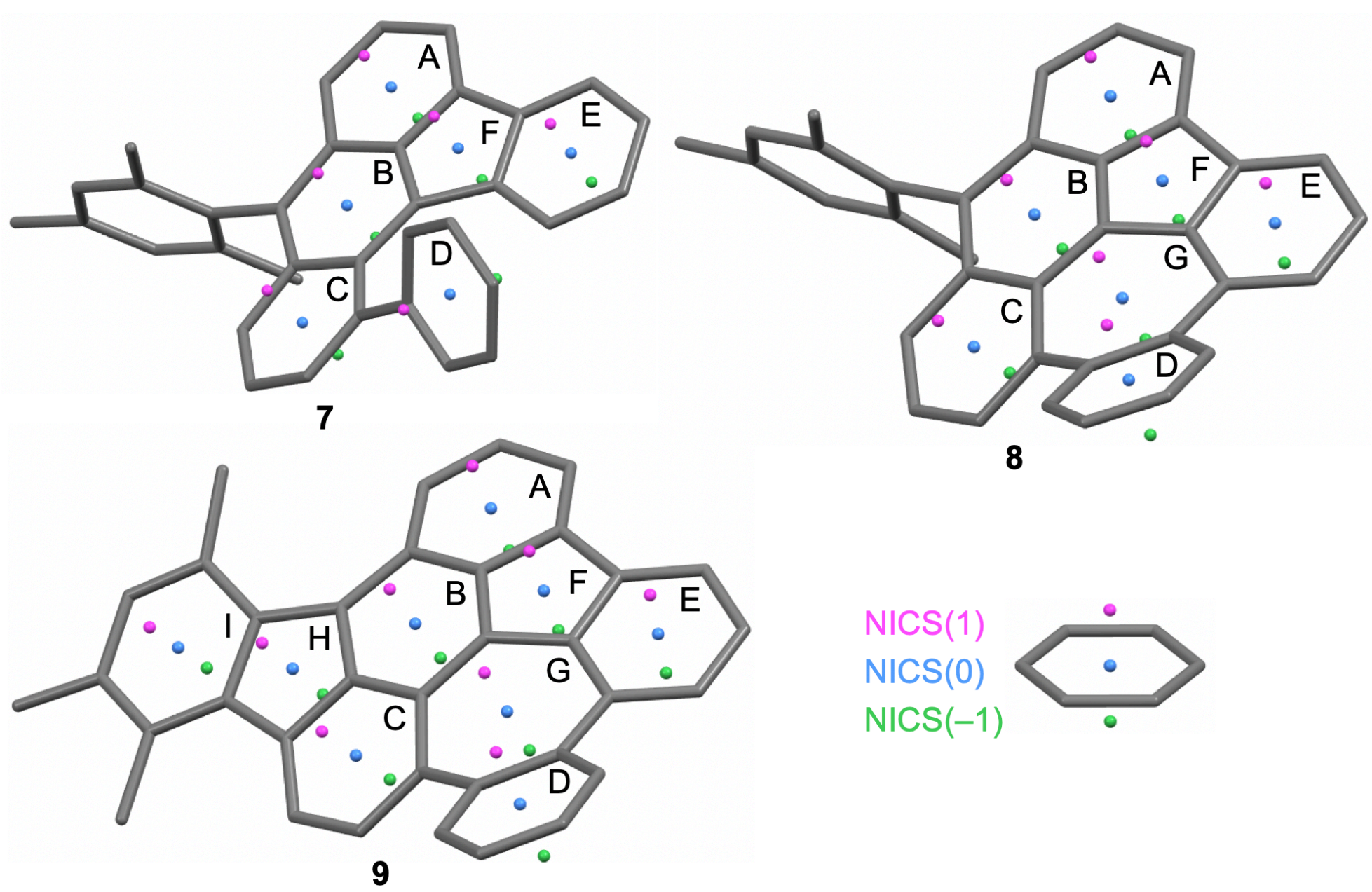

Figure S7. Positions of dummy atoms for calculation of NICS values.

Table S3. NICS values of each benzene ring in **7**–**9** calculated at the GIAO-B3LYP-D3/6-31G(d,p) level.

Compound **7**

Ring [a]	NICS(1)	NICS(–1)	$NICS(1)_{av}$ [b]	NICS(0)
A	–8.92	–9.09	–9.01	–7.02
B	–10.75	–11.29	–11.02	–9.33
C	–9.01	–8.98	–9.00	–7.44
D	–10.30	–9.60	–9.95	–8.63
E	–8.69	–9.32	–9.01	–7.26
F (5)	+1.25	–0.37	+0.88	+5.04

Compound **8**

Ring [a]	NICS(1)	NICS(–1)	$NICS(1)_{av}$ [b]	NICS(0)
A	–8.77	–9.95	–9.36	–7.45
B	–9.67	–12.31	–10.99	–9.34
C	–8.57	–9.08	–8.83	–6.50
D	–9.51	–8.94	–9.23	–7.58
E	–7.19	–7.26	–7.23	–5.28
F (5)	+0.93	–1.19	–0.13	+4.29
G (7)	+3.08	+3.91	+3.50	+7.97

Compound **9**

Ring [a]	NICS(1)	NICS(–1)	$NICS(1)_{av}$ [b]	NICS(0)
A	–8.20	–10.06	–9.13	–7.28
B	–8.90	–12.03	–10.47	–8.89
C	–7.88	–8.98	–8.43	–6.39
D	–10.33	–9.19	–9.76	–8.18
E	–7.50	–8.06	–7.78	–5.92
F (5)	+1.22	–0.88	+0.17	+4.70
G (7)	+1.42	+1.97	+1.70	+5.85
H (5)	–1.77	–2.00	–1.89	+2.00
I	–9.48	–8.67	–9.08	–7.62

[a] Positions of dummy atoms are shown in Figure S6. Numbers in parentheses are the ring size for nonhexagonal rings.
[b] Averages of NICS(1) and NICS(–1).[S17]

ACID

The anisotropy of the induced current density (ACID) plots was generated with the ACID-3.0.4 program.[S19] The data were calculated at the CSGT-B3LYP-D3BJ/6-31G(d,p) using the optimized structures at the B3LYP-D3BJ/6-31G(d,p) level. The magnetic field was applied in the direction as shown in Figure S8. The ACID plots of **7–9** prepared from the π orbitals contributions and the π+σ orbitals contributions are shown in Figures 7b and S8, respectively. Clockwise and counter clockwise arrows indicate diatropic and paratropic ring currents, respectively.

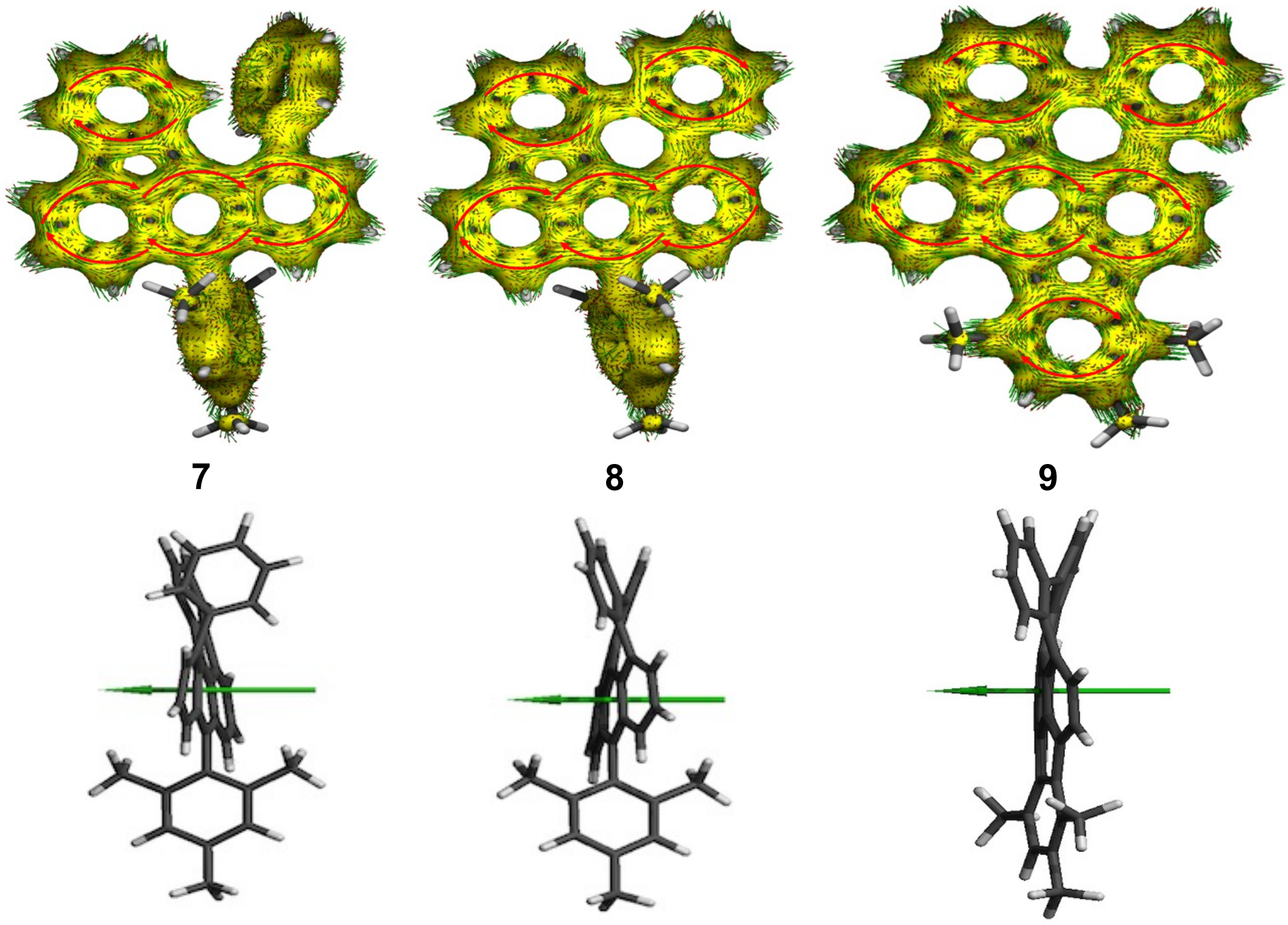

Figure S8. ACID plots of contributions from π+σ orbitals calculated at the CSGT-B3LYP-D3BJ/6-31G(d,p) level (isovalue: 0.050) of compounds **7**–**9**. Global diatropic ring currents are indicated by red bold arrows. Direction of the magnetic field was shown in the bottom structures.

Calculations of radical cation species

The structures of radical cations of **6**, **7**, and **8** were optimized at the UB3LYP-D3/6-31G(d,p) level. The calculated Mulliken spin density and charge values at selected C atoms are listed in Table S4 with the Mulliken spin density maps.

Table S4. Mulliken spin density and Mulliken charge values on representative C atoms in radical cations **6**$^{•+}$, **7**$^{•+}$, and **8**$^{•+}$ calculated at the UB3LYP-D3/6-31G(d,p) level. Mulliken spin densities are visualized by blue and green isosurfeces (isovalue 0.0040 a.u.).[a]

Radical cation **6**$^{•+}$

	Mulliken spin density	Mulliken charge
C1	+0.1018	+0.0304
C4	+0.1539	–0.1153
C5	+0.1538	–0.1151
C8	+0.1017	+0.0304
C9	+0.2192	–0.1816
C10	+0.2891	–0.0524
C2’	+0.0235	–0.1117

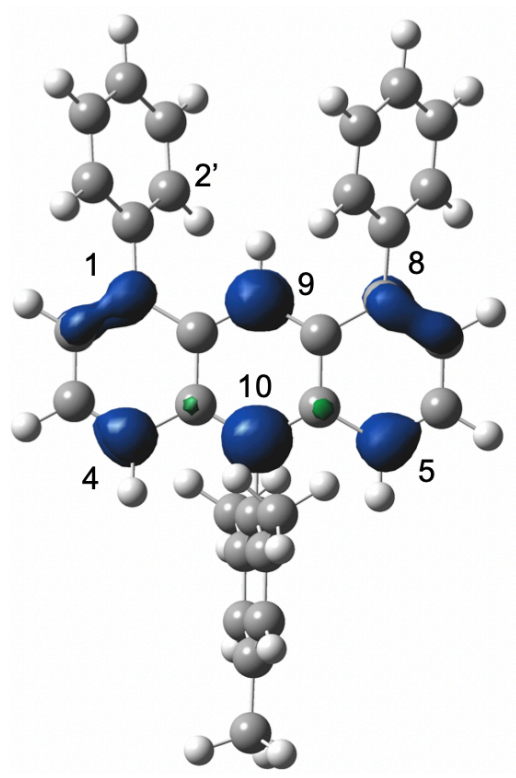

6$^{•+}$

Radical cation **7**$^{•+}$

	Mulliken spin density	Mulliken charge
C1	+0.1090	–0.0823
C4b	+0.0433	+0.0808
C7	+0.0629	–0.1186
C8	+0.2755	–0.0601
C9	+0.1347	–0.1285
C12	+0.1330	+0.0495
C12b	+0.2491	+0.0385
C2’	+0.0249	–0.0833

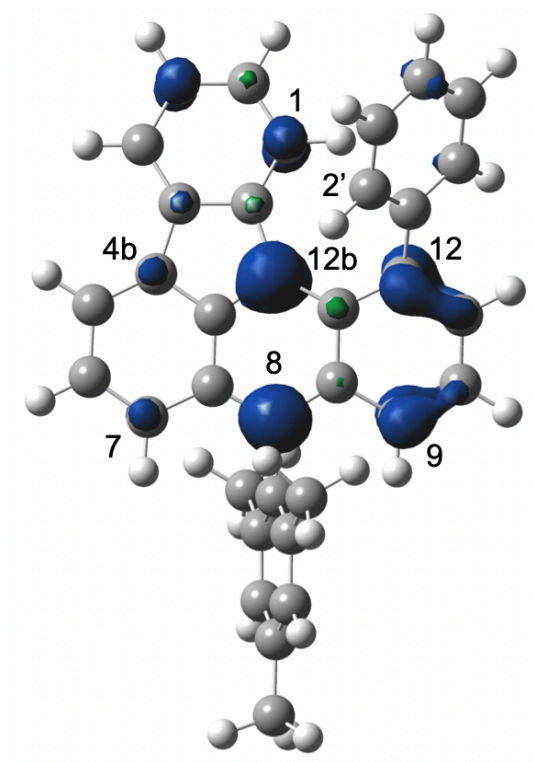

7$^{•+}$

Radical cation **8**$^{•+}$

	Mulliken spin density	Mulliken charge
C4b	+0.1076	+0.0813
C10	+0.0627	–0.1287
C11	+0.2930	–0.0655
C12	+0.1645	–0.1333
C14	+0.0850	–0.1341
C15	+0.0827	+0.0450
C4b^{2}	+0.1542	+0.0030
C2’	+0.0116	+0.1434

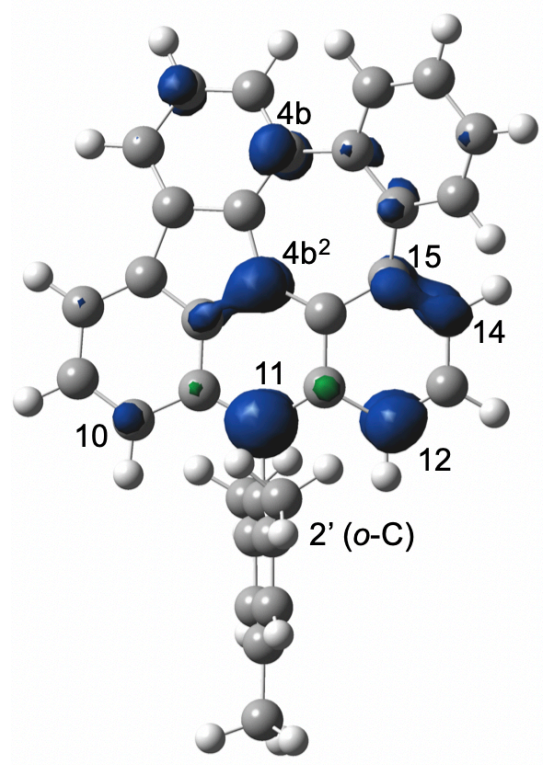

8$^{•+}$

[a] Numberings are shown in the structures.

Calculations of reaction mechanism

The intermediates and transition states during the reaction from **8** to **9** were calculated at the B3LYP-D3/6-31G(d,p) level for the arenium cation mechanism and at the UB3LYP-D3/6-31G(d,p) level for the radical cation mechanism. The solvent effect (dichloromethane) was incorporated using the implicit solvation model based on Integral Equation Formalism Polarizable Continuum model (IEFPCM). Transition states were searched using SCAN method. The calculated structures and their energies are shown in Figure S9 and Table S5, respectively.

Arenium ion mechanism

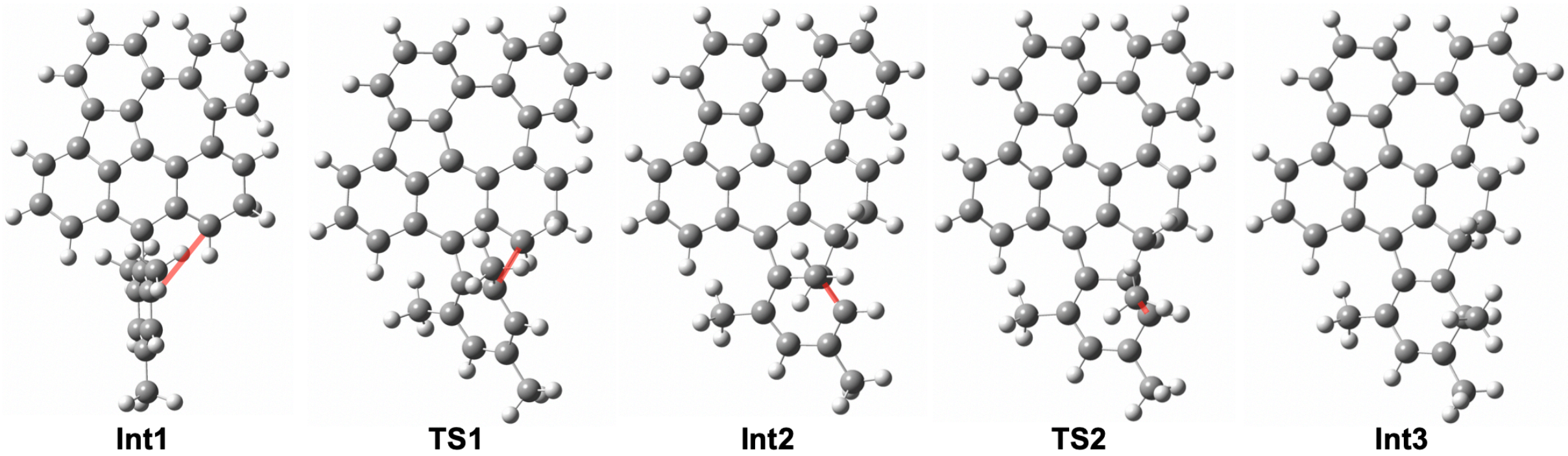

Radical ion mechanism

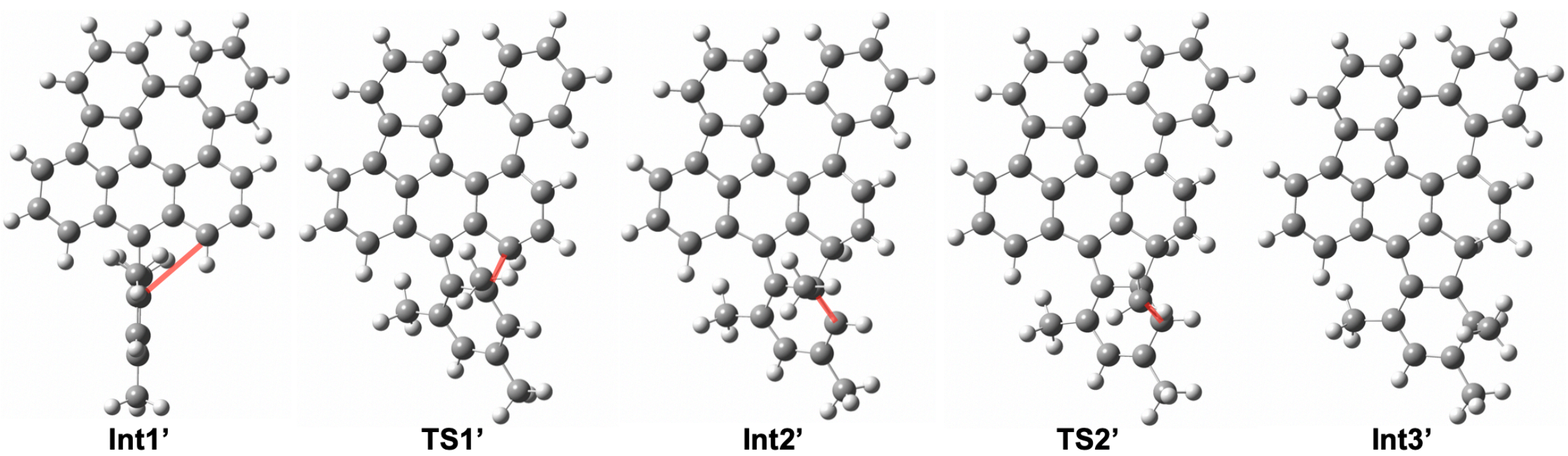

Figure S9. Calculated structures of intermediates and transition states during the reaction from **8** to **9** for (a) arenium ion mechanism at the B3LYP-D3/6-31G(d,p) level and (b) radical cation mechanism at the UB3LYP-D3/6-31G(d,p) level. Forming bonds are highlighted in red.

Table S5. Total energies and Gibbs free energies of intermediates and transition states during the reaction from **8** to **9** calculated at the (U)B3LYP-D3/6-31G(d,p) level.[a]

	E/ hartree	ΔE/ kJ mol^{-1}	G_{298}/ hartree	ΔG_{298}/ kJ mol^{-1}
arenium ion mechanism				
Int1	−1348.254806	0	−1348.309809	0
TS1	−1348.221092	88.5	−1348.274182	93.5
Int2	−1348.242295	32.8	−1348.294800	39.4
TS2	−1348.209750	118.3	−1348.262016	125.5
Int3	−1348.240160	38.5	−1348.292815	44.6
radical cation mechanism				
Int1’	−1347.683207	0	−1347.738842	0
TS1’	−1347.621726	161.4	−1347.674578	168.7
Int2’	−1347.622658	159.0	−1347.675130	167.3
TS2’	−1347.590547	243.3	−1347.642837	252.1
Int3’	−1347.618535	170.0	−1347.671932	175.7

[a] See Figures 8 and S9.

5. References

[S1] A. Shirai, H. Sano, Y. Nakamura, M. Takashika, H. Otani, M. Hasegawa, S. Kato, M. Iyoda, "10-Mesityl-1,8-diphenylanthracene Dimer: Synthesis, Structure, and Properties," *The Journal of Organic Chemistry* **83** (2018): 3857-3863.

[S2] M. Yoshikawa, S. Imigi, K. Wakamatsu, T. Iwanaga, S. Toyota, "Strained and Fluxional Macrocyclic Framework of Anthracene–Diacetylene Cyclic Pentamers," *Chemistry Letters* **42** (2013): 559-561.

[S3] N. Agarwal, M. Patil, M. Patil, "Synthesis of Highly Emissive 1,8-Diaryl Anthracene Derivatives and Fabrication of Their Micro/nanostructures ," *RSC Advances* **5** (2015): 98447-98455.

[S4] C. Hoffend, M. Diefenbach, E. Januszewski, M. Bolte, H.-W. Lerner, M. C. Holthausen, M. Wagner, "Effects of Boron Doping on the Structural and Optoelectronic Properties of 9,10-Diarylanthracenes," *Dalton Transactions* **42** (2013): 13826-13837.

[S5] G. M. Sheldrick, "*SHELXT* – Integrated Space-Group and Crystal-Structure Determination," *Acta Crystallographica Section A, Foundations and Advances* **71** (2015): 3-8.

[S6] G. M. Sheldrick, "Crystal Structure Refinement with SHELXL," *Acta Crystallographica Section C, Structural Chemistry* **71** (2015): 3-8.

[S7] D. Kratzert, J. J. Holstein, I. Krossing, "*DSR*: enhanced modelling and refinement of disordered structures with *SHELXL*," *Journal of Applied Crystallography* **48** (2015): 933-938.

[S8] D. Kratzert, I. Krossing, "Recent improvements in *DSR*," *Journal of Applied Crystallography* **51**, (2018): 928-934.

[S9] O. V. Dolomanov, L. J. Bourhis, R. J. Gildea, J. A. K. Howard, H. Puschmann, "*OLEX2*: A Complete Structure Solution, Refinement and Analysis Program," *Journal of Applied Crystallography* **42** (2009): 339-341.

[S10] T. M. Krygowski, M. K. Cyrański, "Structural Aspects of Aromaticity," *Chemical Reviews* **101** (2001): 1385-1420.

[S11] J. Kruszewski, T. M. Krygowski, "Definition of Aromaticity Basing on the Harmonic Oscillator Model," *Tetrahedron Letters* **13** (1972): 3839-3842.

[S12] S. Toyota, S. Ban, M. Hara, M. Kawamura, H. Ikeda, E. Tsurumaki, "Synthesis and Properties of Rubicene-Based Aromatic π-Conjugated Compounds as Five-Membered Ring Embedded Planar Nanographenes," *Chemistry – A European Journal* **29** (2023): e202301346.

[S13] X. Gu, X. Xu, H. Li. Z. Liu, Q. Miao, "Synthesis, Molecular Packing, and Thin Film Transistors of Dibenzo[*a*,*m*]rubicenes," *Journal of the American Chemical Society* **137** (2015): 16203-16208.

[S14] J. Liu, S. Osella, J. Ma, R. Berger, D. Beljonne, D. Schollmeyer, X. Feng, K. Müllen, "Fused Dibenzo[*a*,*m*]rubicene: A New Bowl-Shaped Subunit of C_{70} Containing Two Pentagons," *Journal of the American Chemical Society* **138** (2016): 8364-8367.

[S15] Gaussian 16, Revision C.01: M. J. Frisch, G. W. Trucks, H. B. Schlegel, G. E. Scuseria, M. A. Robb, J. R. Cheeseman, G. Scalmani, V. Barone, G. A. Petersson, H. Nakatsuji, X. Li, M. Caricato, A. V. Marenich, J. Bloino, B. G. Janesko, R. Gomperts, B. Mennucci, H. P. Hratchian, J. V. Ortiz, A. F. Izmaylov, J. L. Sonnenberg, D. Williams-Young, F. Ding, F. Lipparini, F. Egidi, J. Goings, B. Peng, A. Petrone, T. Henderson, D. Ranasinghe, V. G. Zakrzewski, J. Gao, N. Rega, G. Zheng, W. Liang, M. Hada, M. Ehara, K. Toyota, R. Fukuda, J. Hasegawa, M. Ishida, T. Nakajima, Y. Honda, O. Kitao, H. Nakai, T. Vreven, K. Throssell, J. A. Montgomery, Jr., J. E. Peralta, F. Ogliaro, M. J.

Bearpark, J. J. Heyd, E. N. Brothers, K. N. Kudin, V. N. Staroverov, T. A. Keith, R. Kobayashi, J. Normand, K. Raghavachari, A. P. Rendell, J. C. Burant, S. S. Iyengar, J. Tomasi, M. Cossi, J. M. Millam, M. Klene, C. Adamo, R. Cammi, J. W. Ochterski, R. L. Martin, K. Morokuma, O. Farkas, J. B. Foresman, D. J. Fox, Gaussian, Inc., Wallingford CT, 2016.

[S16] H. Fallah-Bagher-Shaidaei, C. S. Wannere, C. Corminboeuf, R. Puchta, P. v. R. Schleyer, "Which NICS Aromaticity Index for Planar π Rings Is Best?" *Organic Letters* **8** (2006): 863-866.

[S17] J. Cz. Dobrowolski, P. F. J. Lipińskic, On Splitting of the NICS(1) Magnetic Aromaticity Index. *RSC Advances* **6** (2016): 23900-23904.

[S18] J. Borstelmann, J. Bergner, F. Rominger, M. Kivala, "A Negatively Curved π-Expanded Pyracylene Comprising a Tropylium Cation," *Angewandte Chemie International Edition* **62** (2023): e202312740.

[S19] D. Geuenich, K. Hess, F. Köhler, R. Herges, "Anisotropy of the Induced Current Density (ACID), a General Method To Quantify and Visualize Electronic Delocalization," *Chemical Reviews* **105** (2005): 3758-3772.

6. NMR charts

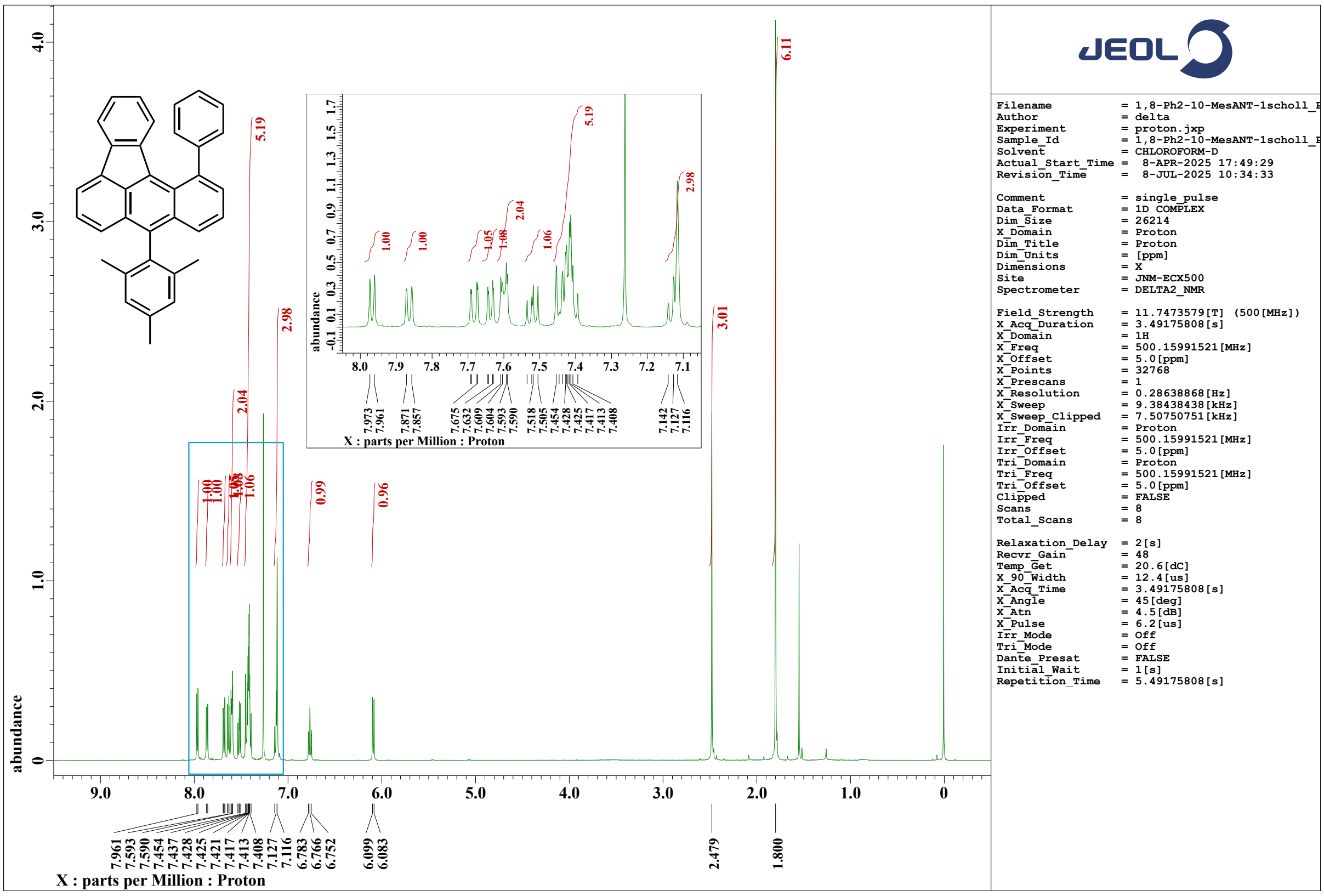

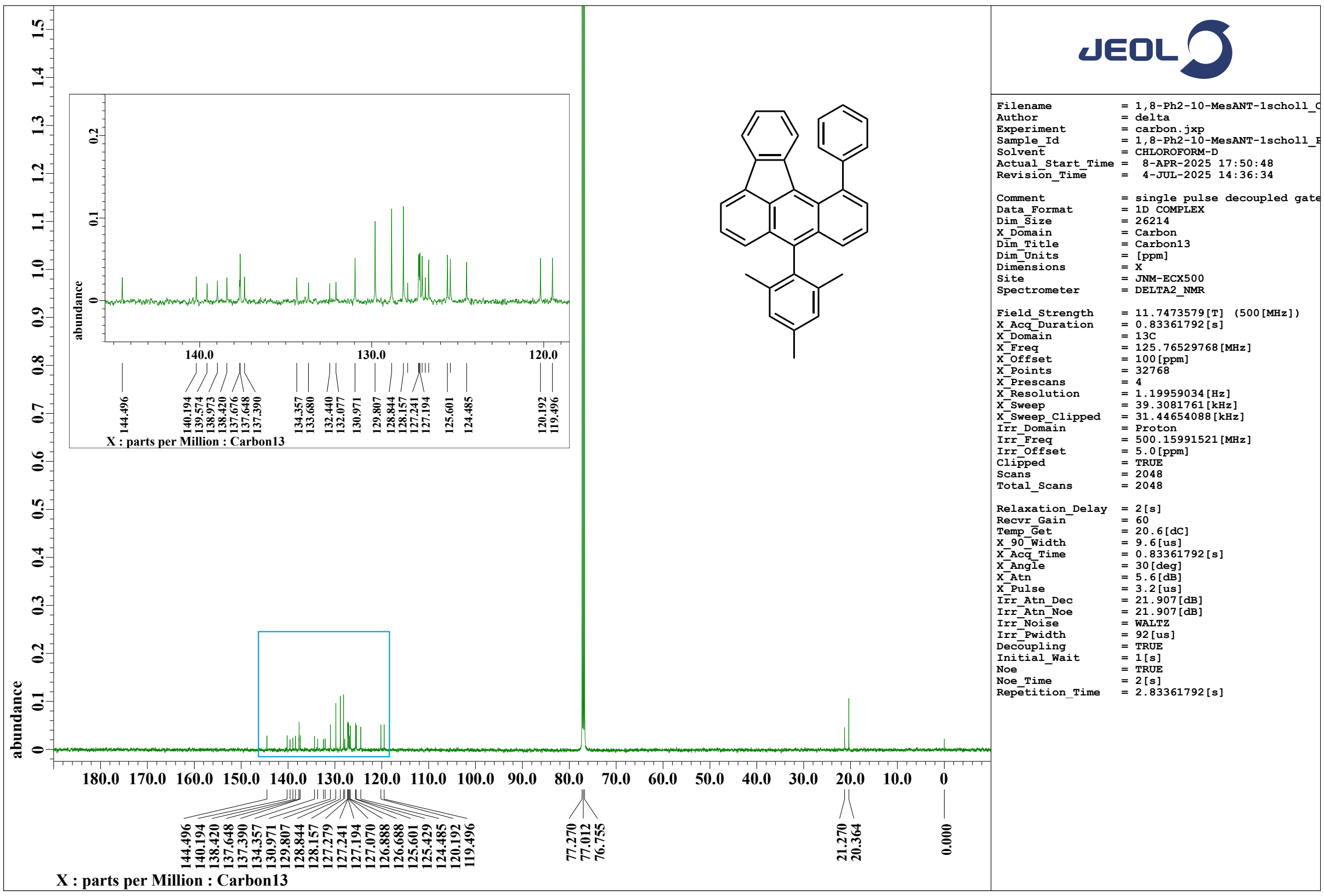

Figure S10. ^{1}H NMR (top, 500 MHz) and ^{13}C NMR (bottom, 125 MHz) spectra of **7** in $CDCl_3$.

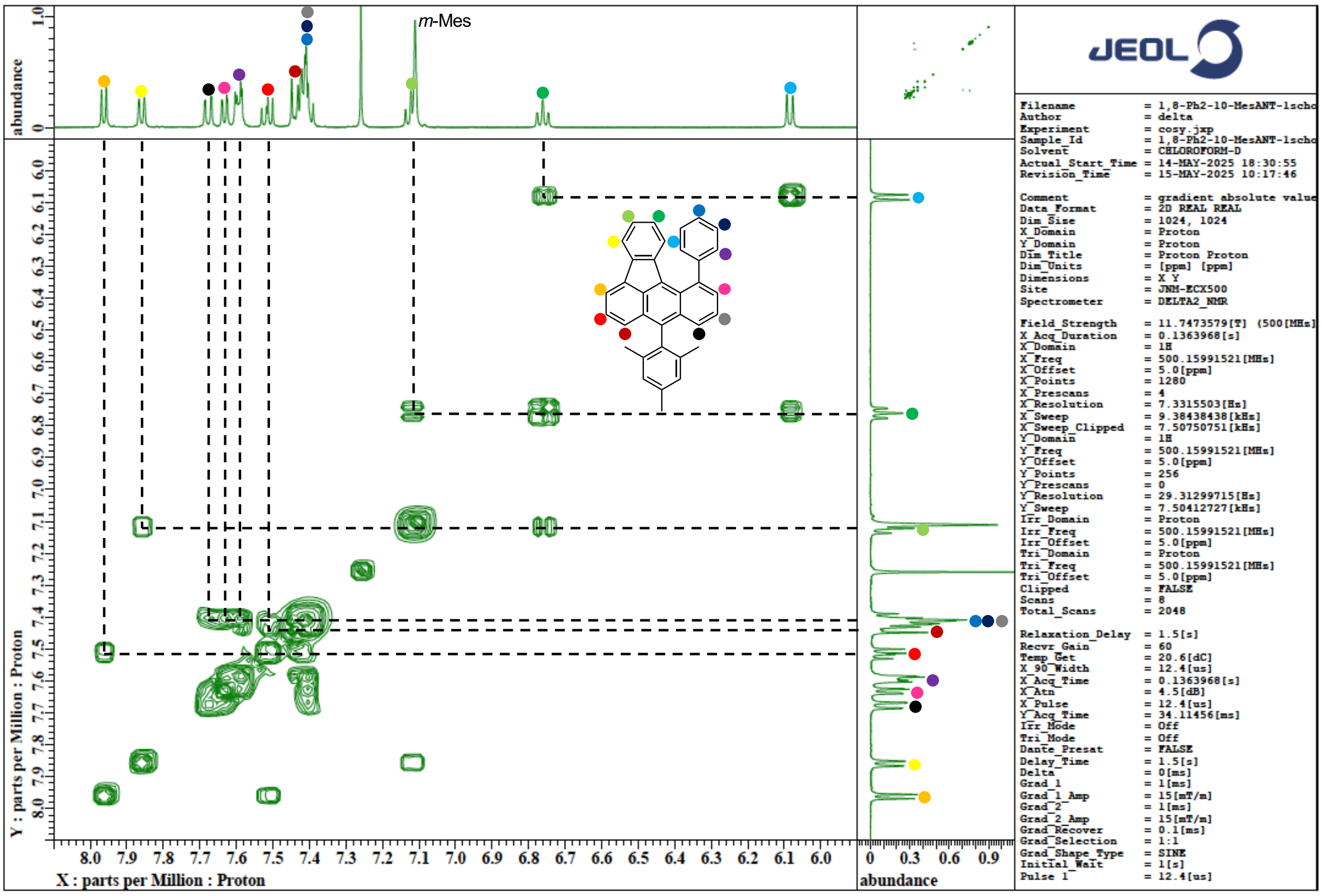

Figure S11. ^{1}H–^{1}H COSY spectrum of **7** at 500 MHz in $CDCl_3$.

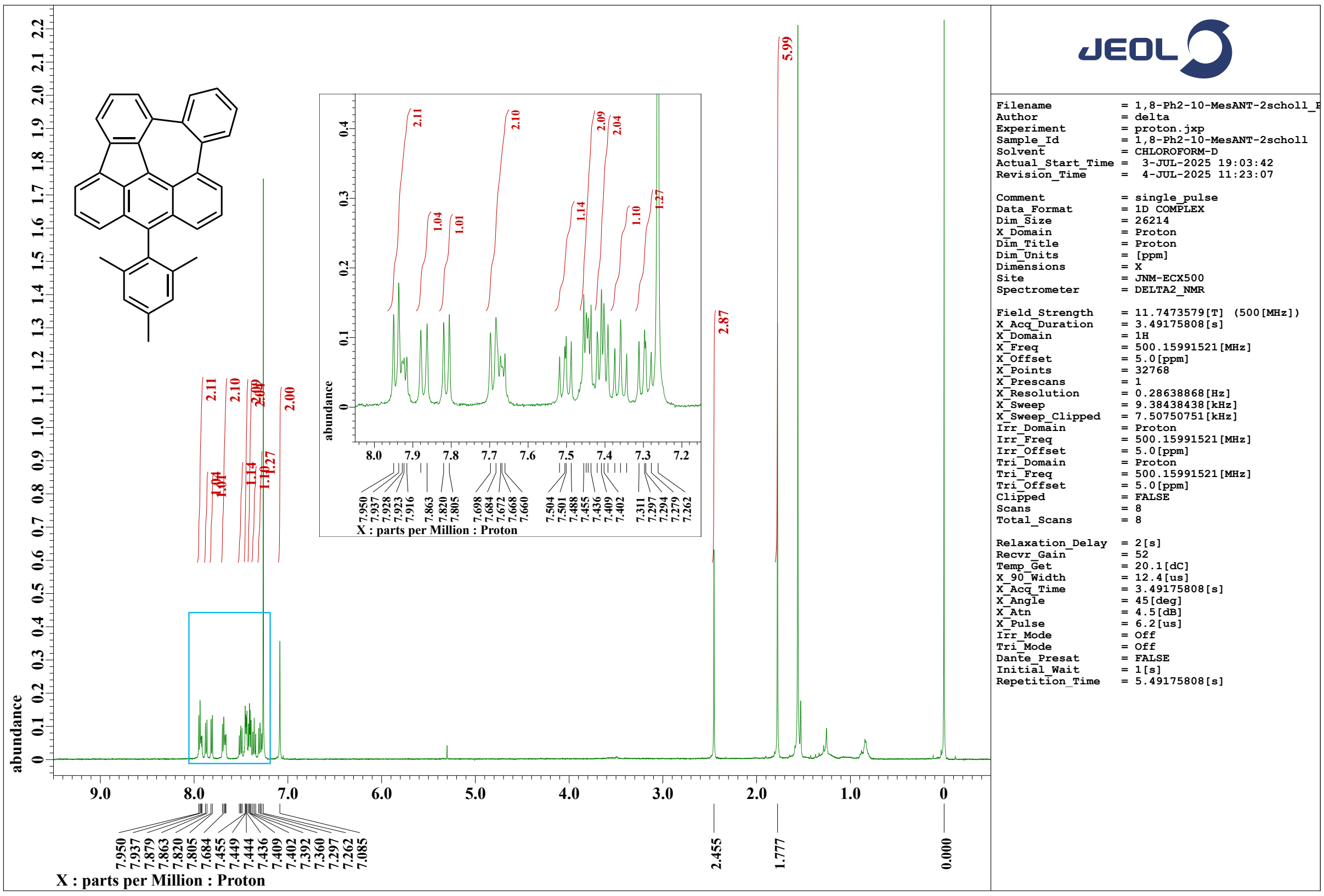

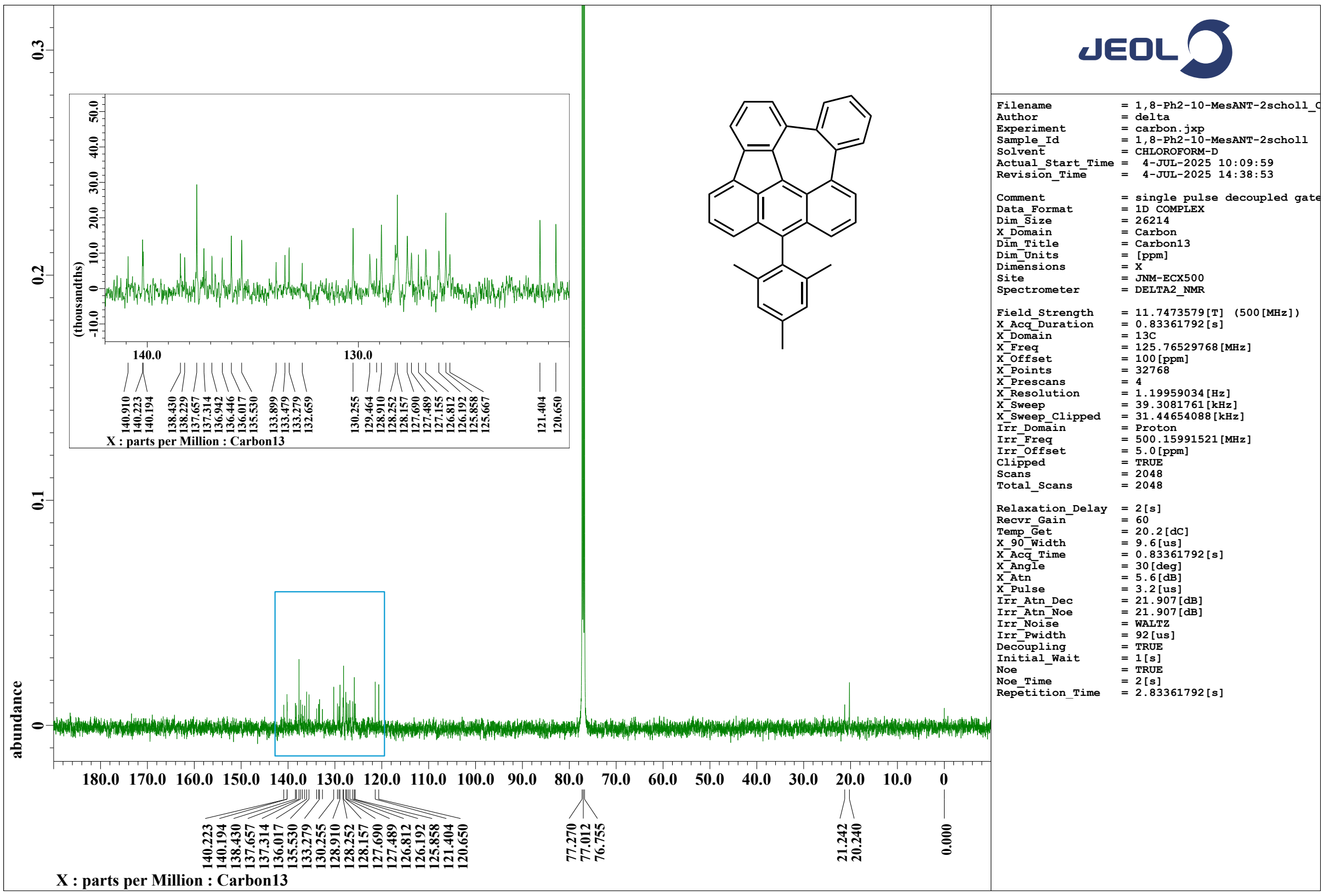

Figure S12. ^{1}H NMR (top, 500 MHz) and ^{13}C NMR (bottom, 125 MHz) spectra of **8** in $CDCl_3$.

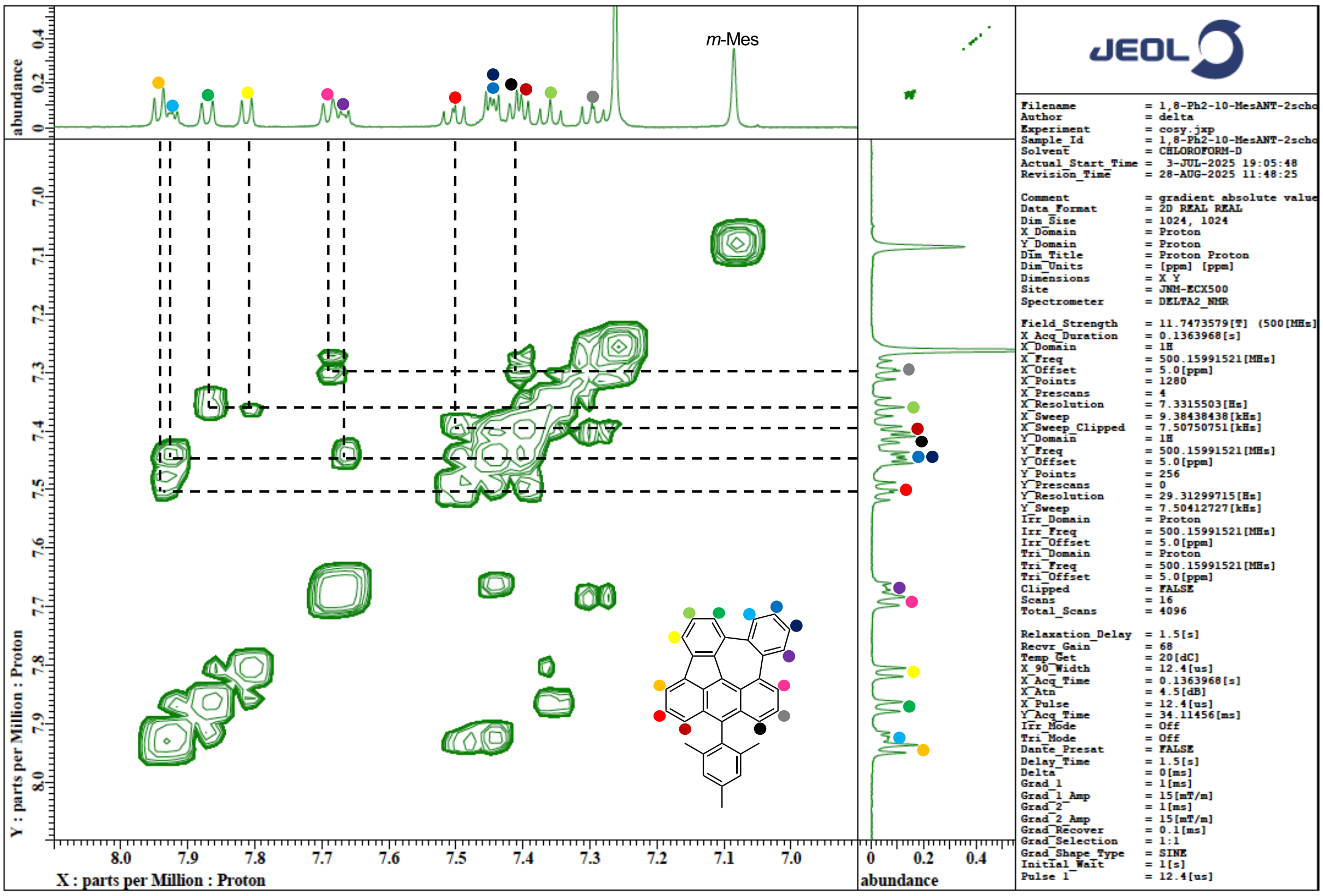

Figure S13. ^{1}H–^{1}H COSY spectrum of **8** at 500 MHz in $CDCl_3$.

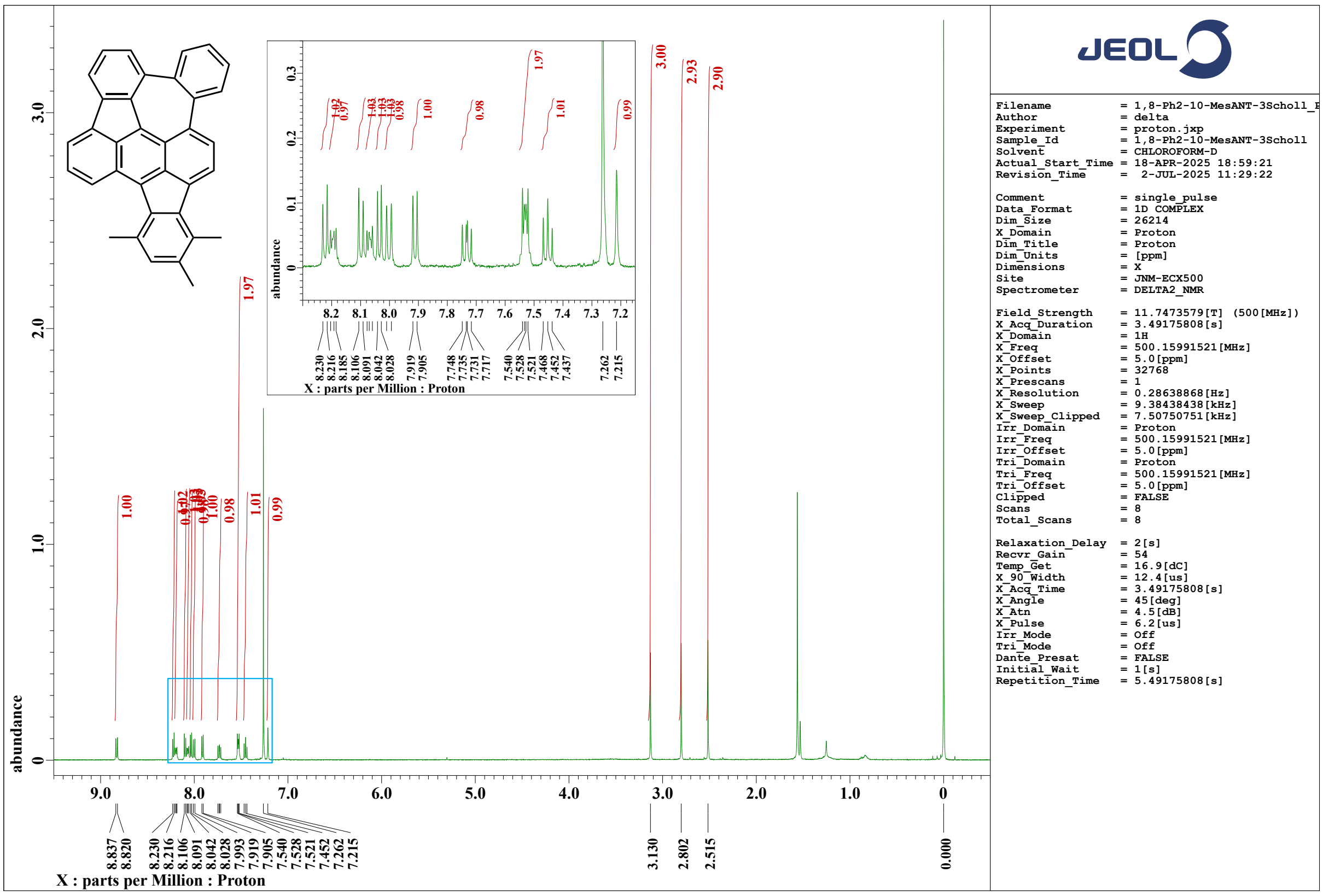

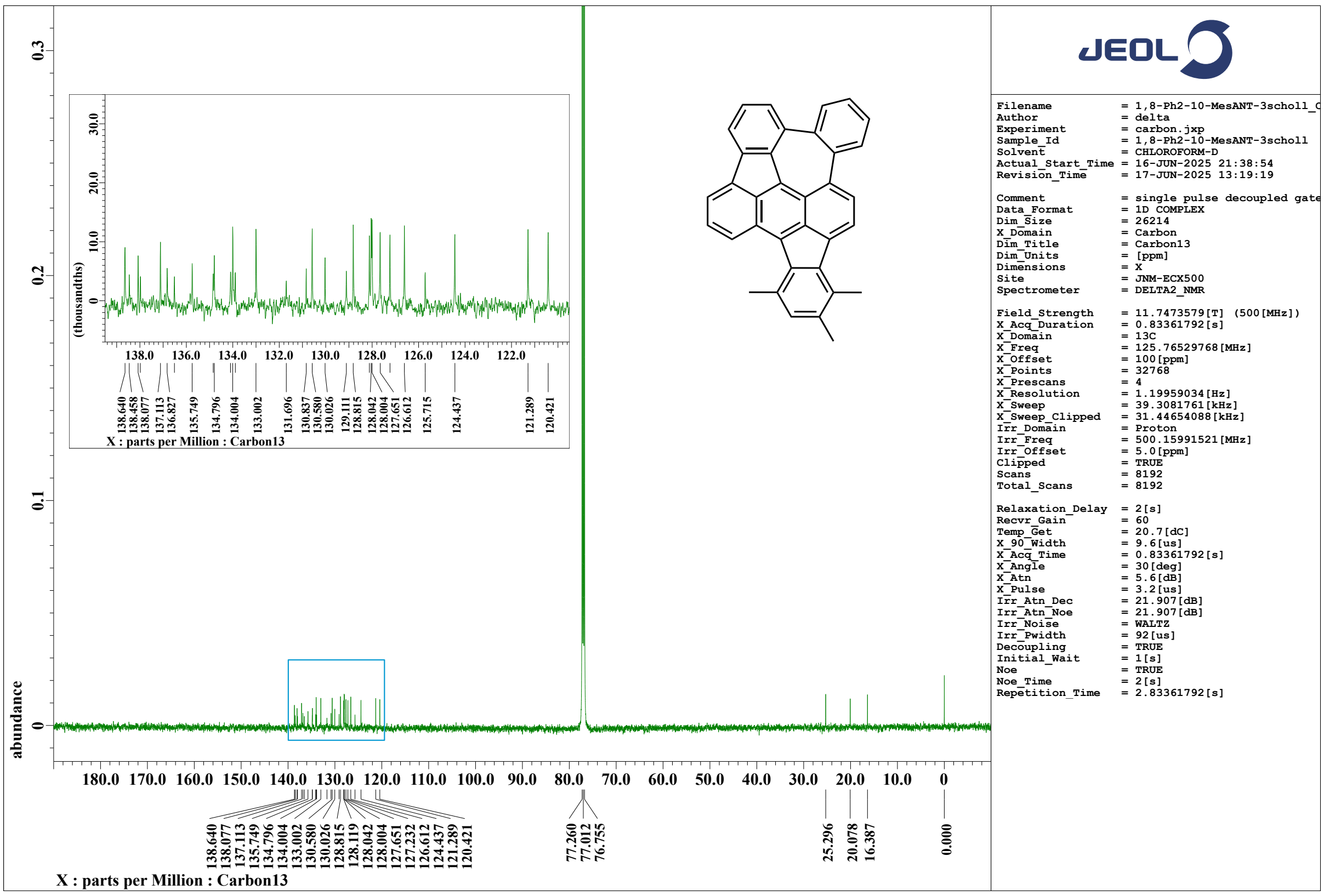

Figure S14. ^{1}H NMR (top, 500 MHz) and ^{13}C NMR (bottom, 125 MHz) spectra of **9** in $CDCl_3$.

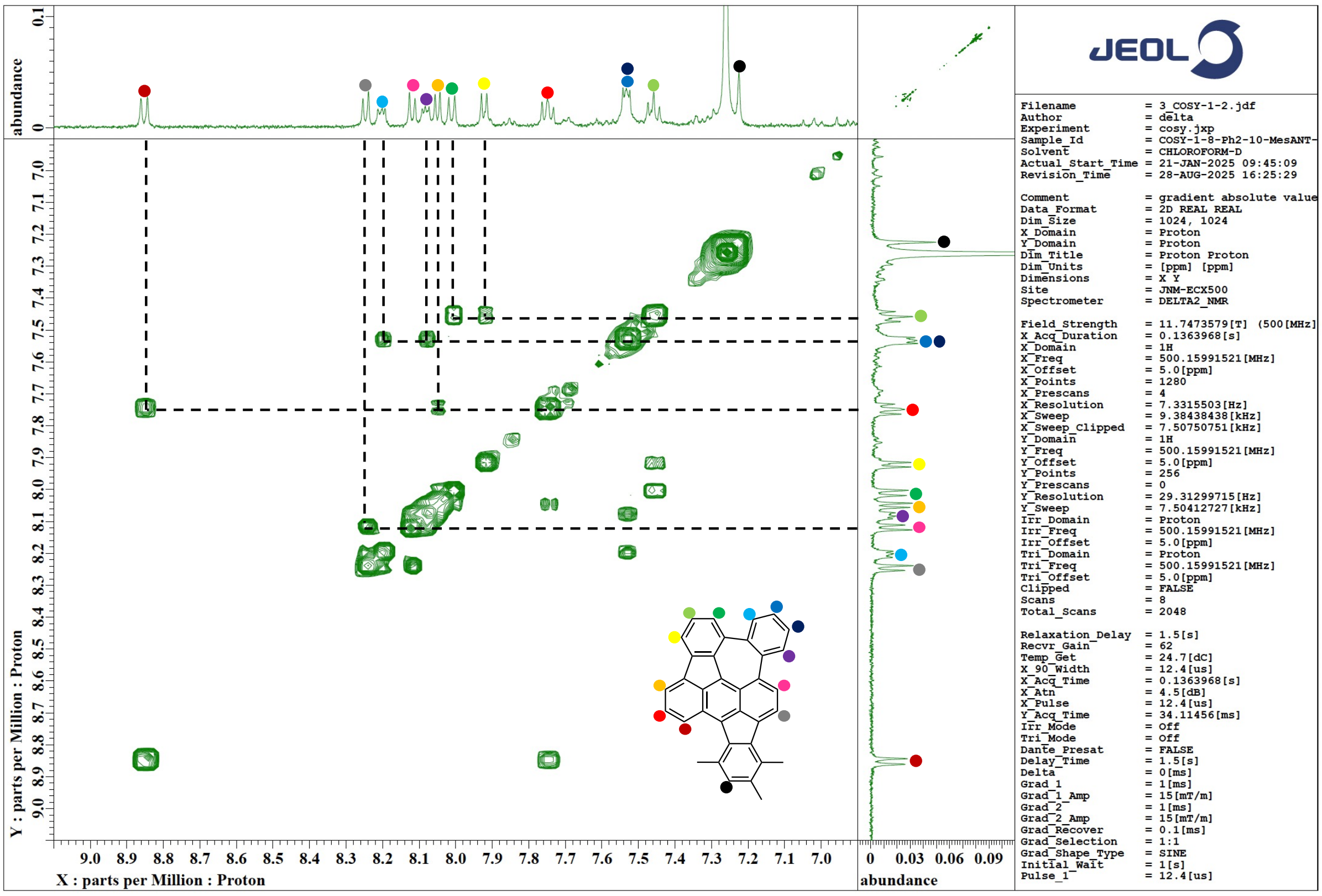

Figure S15. ^{1}H–^{1}H COSY spectrum of **9** at 500 MHz in $CDCl_3$.

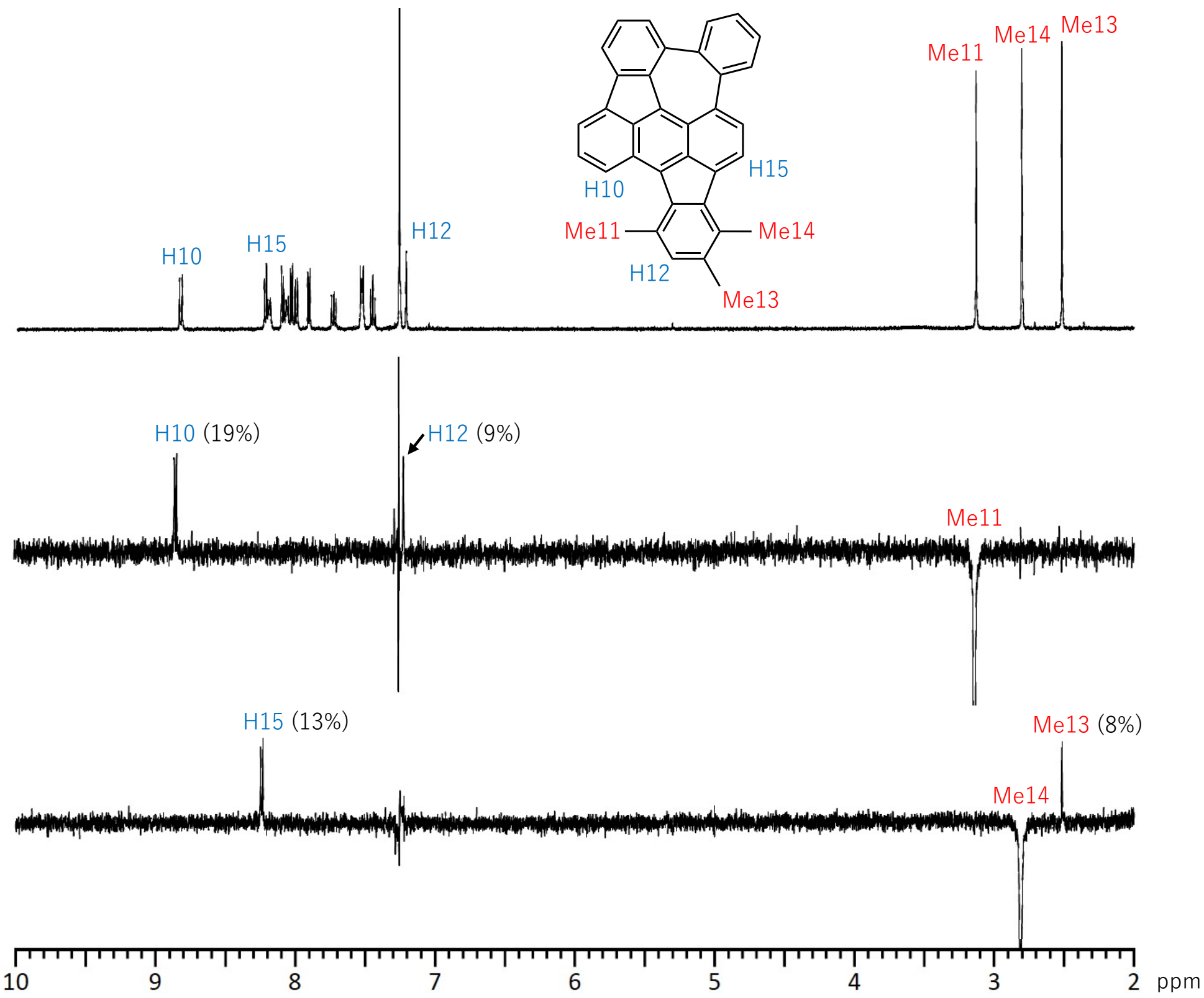

Figure S16. 1D differential NOE spectra of **9** at 500 MHz in $CDCl_3$. Top: nonirradiated, middle: irradiated at Me11, and bottom: irradiated at Me14.

7. Coordinates of calculated structures

Table S6. Atomic coordinates of calculated structure of **6** at the B3LYP-D3/6-31G(d,p) level. Number of imaginary frequencies = 0, Total energy = –1350.295526 h

Atom	*x*	*y*	*z*
C	–0.398887	–3.661269	–0.024615
C	1.020702	–3.659260	0.022841
C	1.716820	–2.480435	0.019960
C	1.028971	–1.226523	–0.007368
C	–0.415802	–1.225350	–0.038456
C	–1.120203	–2.488577	–0.070586
C	1.729235	–0.000756	0.000085
C	1.030020	1.225591	0.007573
C	–0.414780	1.225644	0.038572
C	–1.089912	0.000435	0.000010
C	1.718909	2.478927	–0.019671
C	1.023810	3.658345	–0.022609
C	–0.395780	3.661558	0.024723
C	–1.118108	2.489484	0.070651
C	–2.606072	–2.551136	–0.136043
C	–2.603922	2.553344	0.136004
C	–3.324006	–1.913692	–1.162148
C	–4.713965	–1.998204	–1.219156
C	–5.414090	–2.727236	–0.255580
C	–4.712892	–3.373670	0.763329
C	–3.322020	–3.285492	0.822194
C	–3.319121	3.288675	–0.822054
C	–4.709918	3.378093	–0.763300
C	–5.411810	2.731948	0.255310
C	–4.712437	2.001962	1.218714
C	–3.322551	1.916216	1.161824
C	3.226075	–0.001294	0.000057
C	3.928620	–0.033673	–1.220080
C	5.326500	–0.037372	–1.199237
C	6.044080	–0.008548	0.000169
C	5.326594	0.019583	1.199566
C	3.928647	0.025230	1.220243
C	3.177128	0.054668	2.530138
C	3.177034	–0.068347	–2.529807
C	7.554199	0.022089	–0.000825
H	–0.930222	–4.608002	–0.051669
H	1.552337	–4.605999	0.047587
H	2.800978	–2.478194	0.043184
H	–2.170083	0.000878	–0.000082
H	2.803058	2.475655	–0.042786
H	1.556230	4.604649	–0.047260
H	–0.926305	4.608745	0.051751
H	–2.783458	–1.354328	–1.919294
H	–5.250202	–1.496646	–2.019422
H	–6.497425	–2.790514	–0.299093
H	–5.248882	–3.941477	1.518569
H	–2.777751	–3.774790	1.624614
H	–2.774346	3.777722	–1.624283
H	–5.245310	3.946648	–1.518401
H	–6.495093	2.796173	0.298728
H	–5.249204	1.500619	2.018759
H	–2.782602	1.356138	1.918868
H	5.867842	–0.064890	–2.142475
H	5.867943	0.036120	2.143048
H	3.862596	0.057849	3.381631
H	2.540709	0.943607	2.598681
H	2.515654	–0.813317	2.624674
H	2.541140	–0.957894	–2.595484
H	2.515045	0.798926	–2.627056
H	3.862448	–0.073777	–3.381334
H	7.965507	–0.445385	0.899037
H	7.965347	–0.497645	–0.871662
H	7.927621	1.053389	–0.031405

Table S7. Atomic coordinates of calculated structure of **7** at the B3LYP-D3/6-31G(d,p) level. Number of imaginary frequencies = 0, Total energy = –1349.103989 h

Atom	*x*	*y*	*z*
C	0.076748	3.895384	0.569041
C	–1.331855	3.724618	0.731201
C	–1.941900	2.500593	0.569804
C	–1.163783	1.341723	0.247694
C	0.229221	1.524069	0.119804
C	0.846418	2.802089	0.253620
C	–1.667722	0.052786	–0.009245
C	–0.761720	–1.005101	–0.277359
C	0.692458	–0.820795	–0.255406
C	1.169074	0.514498	–0.171264
C	–1.290838	–2.298987	–0.582065
C	–0.471776	–3.374524	–0.785819
C	0.923342	–3.233977	–0.599699
C	1.507684	–2.017154	–0.306760
C	2.264690	2.604613	–0.047544
C	2.937398	–2.040158	0.095689
C	–3.145172	–0.175430	0.001414
C	2.471216	1.222458	–0.347583
C	3.725390	0.828312	–0.817448
C	4.757715	1.768755	–0.913748
C	4.557756	3.105119	–0.562474
C	3.295986	3.532131	–0.138345
C	3.346001	–1.452046	1.303086
C	4.677292	–1.511268	1.708001
C	5.625960	–2.162249	0.916638
C	5.229834	–2.764629	–0.279029
C	3.895979	–2.709399	–0.682618
C	–3.903056	0.084727	–1.157105
C	–5.285809	–0.117835	–1.122455
C	–5.935200	–0.564650	0.032072
C	–5.163079	–0.817021	1.169733
C	–3.777664	–0.629894	1.175128
C	–2.966683	–0.917390	2.416767
C	–7.434965	–0.741080	0.058396
C	–3.226087	0.567541	–2.418206
H	3.116097	4.576867	0.100038
H	2.612945	–0.936179	1.914354
H	0.516647	4.881584	0.688115
H	–1.936845	4.592144	0.978366
H	–3.016855	2.400286	0.678407
H	–2.367739	–2.408886	–0.641942
H	–0.888724	–4.348509	–1.023121
H	1.550042	–4.120582	–0.616046
H	3.918148	–0.191320	–1.115119
H	5.730937	1.444463	–1.270484
H	5.374893	3.816390	–0.638578
H	4.975870	–1.043751	2.641608
H	6.665337	–2.199581	1.228992
H	5.960650	–3.271890	–0.902348
H	3.593232	–3.159093	–1.623721
H	–5.869519	0.077250	–2.019455
H	–5.650573	–1.169757	2.075988
H	–3.607951	–1.222375	3.247736
H	–2.238833	–1.715738	2.235209
H	–2.395655	–0.036508	2.729069
H	–7.824400	–1.010784	–0.928023
H	–7.734995	–1.519470	0.766543
H	–7.935343	0.186613	0.363164
H	–2.440316	–0.126531	–2.735380
H	–3.941827	0.672962	–3.237561
H	–2.742328	1.537427	–2.259311

Table S8. Atomic coordinates of calculated structure of **8** at the B3LYP-D3/6-31G(d,p) level. Number of imaginary frequencies = 0, Total energy = –1347.919071 h

Atom	*x*	*y*	*z*
C	0.076748	3.895384	0.569041
C	–1.331855	3.724618	0.731201
C	–1.941900	2.500593	0.569804
C	–1.163783	1.341723	0.247694
C	0.229221	1.524069	0.119804
C	0.846418	2.802089	0.253620
C	–1.667722	0.052786	–0.009245
C	–0.761720	–1.005101	–0.277359
C	0.692458	–0.820795	–0.255406
C	1.169074	0.514498	–0.171264
C	–1.290838	–2.298987	–0.582065
C	–0.471776	–3.374524	–0.785819
C	0.923342	–3.233977	–0.599699
C	1.507684	–2.017154	–0.306760
C	2.264690	2.604613	–0.047544
C	2.937398	–2.040158	0.095689
C	–3.145172	–0.175430	0.001414
C	2.471216	1.222458	–0.347583
C	3.725390	0.828312	–0.817448
C	4.757715	1.768755	–0.913748
C	4.557756	3.105119	–0.562474
C	3.295986	3.532131	–0.138345
C	3.346001	–1.452046	1.303086
C	4.677292	–1.511268	1.708001
C	5.625960	–2.162249	0.916638
C	5.229834	–2.764629	–0.279029
C	3.895979	–2.709399	–0.682618
C	–3.903056	0.084727	–1.157105
C	–5.285809	–0.117835	–1.122455
C	–5.935200	–0.564650	0.032072
C	–5.163079	–0.817021	1.169733
C	–3.777664	–0.629894	1.175128
C	–2.966683	–0.917390	2.416767
C	–7.434965	–0.741080	0.058396
C	–3.226087	0.567541	–2.418206
H	3.116097	4.576867	0.100038
H	2.612945	–0.936179	1.914354
H	0.516647	4.881584	0.688115
H	–1.936845	4.592144	0.978366
H	–3.016855	2.400286	0.678407
H	–2.367739	–2.408886	–0.641942
H	–0.888724	–4.348509	–1.023121
H	1.550042	–4.120582	–0.616046
H	3.918148	–0.191320	–1.115119
H	5.730937	1.444463	–1.270484
H	5.374893	3.816390	–0.638578
H	4.975870	–1.043751	2.641608
H	6.665337	–2.199581	1.228992
H	5.960650	–3.271890	–0.902348
H	3.593232	–3.159093	–1.623721
H	–5.869519	0.077250	–2.019455
H	–5.650573	–1.169757	2.075988
H	–3.607951	–1.222375	3.247736
H	–2.238833	–1.715738	2.235209
H	–2.395655	–0.036508	2.729069
H	–7.824400	–1.010784	–0.928023
H	–7.734995	–1.519470	0.766543
H	–7.935343	0.186613	0.363164
H	–2.440316	–0.126531	–2.735380
H	–3.941827	0.672962	–3.237561
H	–2.742328	1.537427	–2.259311

Table S9. Atomic coordinates of calculated structure of **9** at the B3LYP-D3/6-31G(d,p) level. Number of imaginary frequencies = 0, Total energy = –1346.727562 h

Atom	*x*	*y*	*z*
C	0.982511	4.282030	–0.071660
C	–0.426964	4.463890	0.018888
C	–1.309146	3.409406	0.145472
C	–0.845189	2.057914	0.231641
C	0.570096	1.913853	0.178622
C	1.475943	3.001672	–0.008100
C	–1.580407	0.837129	0.210464
C	–0.846739	–0.362301	0.006852
C	0.568487	–0.502769	0.009870
C	1.276451	0.696417	0.126313
C	–1.722985	–1.439386	–0.320354
C	–1.148471	–2.634521	–0.701327
C	0.260099	–2.799622	–0.630914
C	1.140734	–1.806710	–0.209164
C	2.809193	2.411279	–0.214005
C	2.554085	–2.158850	0.129175
C	–3.011466	0.453796	0.186561
C	–4.190828	1.122180	0.550121
C	–5.400669	0.434824	0.372181
C	–5.495364	–0.874752	–0.103521
C	–4.310265	–1.590778	–0.378509
C	–3.091798	–0.924186	–0.209824
C	2.730508	–3.482309	0.597712
C	3.959498	–4.013313	0.959175
C	5.087686	–3.200364	0.901337
C	4.944676	–1.882788	0.493046
C	3.714634	–1.318417	0.086656
C	2.678869	0.995325	–0.096997
C	3.764037	0.128492	–0.267572
C	4.971108	0.739133	–0.688642
C	5.099488	2.118934	–0.827816
C	4.024791	2.979034	–0.561507
C	–6.861256	–1.503371	–0.266460
C	–4.338729	–3.034752	–0.818582
C	–4.248649	2.489242	1.188705
H	1.631262	5.139021	–0.228688
H	–0.826607	5.471216	–0.056134
H	–2.366671	3.618779	0.130438
H	–1.739886	–3.471589	–1.049396
H	0.656720	–3.767710	–0.915644
H	–6.320588	0.949558	0.641046
H	1.853313	–4.105459	0.719259
H	4.024196	–5.037072	1.314992
H	6.061386	–3.570922	1.207127
H	5.819034	–1.245394	0.529649
H	5.827608	0.120271	–0.929907
H	6.050471	2.530643	–1.152235
H	4.135019	4.054595	–0.664236
H	–7.650497	–0.791584	–0.011244
H	–7.036694	–1.834137	–1.296663
H	–6.988371	–2.380815	0.378478
H	–5.352008	–3.428954	–0.895481
H	–3.862962	–3.157914	–1.798448
H	–3.790237	–3.669146	–0.112488
H	–3.369844	2.684950	1.806819
H	–4.326965	3.292465	0.445834
H	–5.131513	2.567043	1.829898
