# Supplementary material for: Cyclization of a 1,8‐Diphenylanthracene by Scholl Reactions to Form Five‐ and Seven‐Membered Ring Embedded Polycyclic Aromatic Hydrocarbons
Source: Chemistry. 2026 Jul 20;32(39):e71449. doi: 10.1002/chem.71449 (PMC13715047; doi:10.1002/chem.71449)

checkCIF/PLATON report

Structure factors have been supplied for datablock(s) TS1-1769

THIS REPORT IS FOR GUIDANCE ONLY. IF USED AS PART OF A REVIEW PROCEDURE FOR PUBLICATION, IT SHOULD NOT REPLACE THE EXPERTISE OF AN EXPERIENCED CRYSTALLOGRAPHIC REFEREE.

No syntax errors found. CIF dictionary Interpreting this report

Datablock: TS1-1769

Bond precision: C-C = 0.0015 A Wavelength=3/4

Cell:	a=20.8780(11)	b=9.9460(19)	c=25.1690(19)
	alpha=90	beta=103.376(5)	gamma=90
Temperature:	95 K		

	Calculated	Reported
Volume	5084.6(11)	5084.6(11)
Space group	P 21/n	P 1 21/n 1
Hall group	-P 2yn	-P 2yn
Moiety formula	4(C35 H26), C7 H8	C35 H26, 0.25(C7 H8)
Sum formula	C147 H112	C36.75 H28
Mr	1878.37	469.59
Dx,g cm-3	1.227	1.227
Z	2	8
Mu (mm-1)	0.076	0.077
F000	1988.0	1988.0
F000’	1988.83	
h,k,lmax	32,15,39	32,15,37
Nref	19857	18675
Tmin,Tmax	0.992,0.992	
Tmin’	0.992	

Correction method= Not given

Data completeness= 0.940 Theta(max)= 35.553

R(reflections)= 0.0545(17301) wR2(reflections)= 0.1541(18675)

S = 1.085 Npar= 701

The following ALERTS were generated. Each ALERT has the format
test-name_ALERT_alert-type_alert-level.
Click on the hyperlinks for more details of the test.

Alert level B

```
PLAT250_ALERT_2_B Large U3/U1 Ratio for <U(i,j)> Tensor(Resd    3)         7.8 Note
```

Alert level C

```
DIFMX02_ALERT_1_C  The maximum difference density is > 0.1*ZMAX*0.75
            The relevant atom site should be identified.
PLAT041_ALERT_1_C Calc. and Reported SumFormula    Strings  Differ     Please Check
              Calc: C147 H112
              Rep.: C36.75 H28
PLAT042_ALERT_1_C Calc. and Reported MoietyFormula Strings  Differ     Please Check
              Calc: 4(C35 H26), C7 H8
              Rep.: C35 H26, 0.25(C7 H8)
PLAT052_ALERT_1_C Info on Absorption Correction Method   Not Given     Please Do !
PLAT097_ALERT_2_C Large Reported Max.  (Positive) Residual Density       0.54 eA-3
PLAT906_ALERT_3_C Large K Value in the Analysis of Variance ......      3.631 Check
PLAT911_ALERT_3_C Missing FCF Refl Between Thmin & STh/L=    0.600        123 Report
                2  0  0,   3 11  0,   7  2  0,  13 10  0, -17  0  1,  -8  9  1,
               -5  2  1,  -2  2  1,   7  9  1,  10  7  1,  12 10  1, -10  0  2,
               -9  9  2,  -6  2  2,   6  3  2,  14  5  2,  -8  2  3,  -5  4  3,
               -4  1  3,  -4  2  3,  -4  4  3,  -3  2  3,  -1  4  3,   6  9  3,
               11 10  3,  12  6  3, -12  2  4, -11  4  4,  -7  1  4,  -6  2  4,
               -3  4  4,  -3  5  4,   4  1  4,  12  1  4,  20  0  4, -13  4  5,
              -12  3  5,  -7  6  5,  -5  2  5,  -4  4  5,   5  9  5,  10 10  5,
              -16  5  6,  -7  2  6,  -3  4  6,   3  3  6,   7  5  6,  14  0  6,
              -11  6  7,  -8  1  7,  -4  3  7,  -1  0  7,  -1  6  7,  -1  9  7,
                0  2  7,   9 10  7, -12  1  8, -10  2  8,  -6  4  8,  -3  2  8,
               -2  0  8,  -1  3  8,   0  3  8,   4  9  8,   9 10  8, -17  5  9,
               -7  4  9,   0  2  9,   4  5  9, -17  1 10,  -7  3 10,  -3  4 10,
                0  6 10,   3  6 10,   5  5 10,   8 10 10,  10  5 10,  12  0 10,
              -13  6 11, -12  5 11,  -9  2 11,  -7  1 11,   2  2 11,   3  7 11,
              -16  3 12,   0  1 12,   3  6 12, -12  4 13,  -7  3 13,  -6  8 13,
               -4  3 13,   6  5 13,   8  8 13, -10  1 14,  -8  1 14,  -2  4 14,
```

Alert level G

```
ABSMU01_ALERT_1_G  Calculation of _exptl_absorpt_correction_mu
              not performed for this radiation type.
PLAT002_ALERT_2_G Number of Distance or Angle Restraints on AtSite          7 Note
PLAT003_ALERT_2_G Number of Uiso or U(i,j)  Restrained non-H-Atoms          7 Report
PLAT045_ALERT_1_G Calculated and Reported Z Differ by a Factor ...      0.250 Check
PLAT172_ALERT_4_G The CIF-Embedded .res File Contains DFIX Records          3 Report
PLAT173_ALERT_4_G The CIF-Embedded .res File Contains DANG Records          5 Report
PLAT174_ALERT_4_G The CIF-Embedded .res File Contains FLAT Records          1 Report
PLAT176_ALERT_4_G The CIF-Embedded .res File Contains SADI Records          3 Report
PLAT178_ALERT_4_G The CIF-Embedded .res File Contains SIMU Records          1 Report
PLAT180_ALERT_4_G Check Cell Rounding: # of Values Ending with 0 =          3 Note
PLAT187_ALERT_4_G The CIF-Embedded .res File Contains RIGU Records          1 Report
PLAT191_ALERT_3_G A Non-default SADI Restraint Value has been used     0.0400 Report
PLAT191_ALERT_3_G A Non-default SADI Restraint Value has been used     0.0400 Report
```

PLAT299_ALERT_4_G Atom Site Occupancy Constrained at .... 0.5 Check
C2 C3 C4 C5 C6 C7 C8 H2A
H2B H2C H4A H5A H6 H7 H8
PLAT302_ALERT_4_G Anion/Solvent/Minor-Residue Disorder (Resd 3) 100% Note
PLAT304_ALERT_4_G Non-Integer Number of Atoms in ..... (Resd 3) 7.50 Check
PLAT720_ALERT_4_G Number of Unusual/Non-Standard Labels .... 116 Note
C001 C002 C003 C004 C005 C006 C007 C008
C009 C00A C00B C00C C00D C00E C00F H00F
C00G C00H C00I H00I C00J C00K C00L H00L
C00M H00M C00N C00O C00P H00P C00Q C00R
H00R C00S C00T H00T C00U C00V C00W C00X
H00X C00Y H00Y C00Z C010 C011 C012 C013
H013 C014 C015 H015 C016 H016 C017 H017
C018 H018 C019 H01A H01B H01C C01A H01D
C01B H01V C01C H01E C01D H01F C01E H01G
C01F H01H C01G H01I C01H H01J C01I H01Y
C01J H01Z C01K H01K C01L H01 C01M H01M
C01N Ha C01O H01O C01P H01P C01Q Hb
Hc C01R H01R C01S H01L H01N H01Q C01T
Hd He C01U C01V C01W H01W C01X H01X
C01Y H01S H01T H01U
PLAT789_ALERT_4_G Atoms with Negative _atom_site_disorder_group # 15 Check
PLAT822_ALERT_4_G CIF-embedded .res Contains Negative PART Numbers 1 Check
PLAT860_ALERT_3_G Number of Least-Squares Restraints .... 136 Note
PLAT912_ALERT_4_G Missing # of FCF Reflections Above STh/L= 0.600 1017 Note
PLAT941_ALERT_3_G Average HKL Measurement Multiplicity .... 2.8 Low
PLAT952_ALERT_5_G Calculated (ThMax) and CIF-Reported Lmax Differ. 2 Units
PLAT958_ALERT_1_G Calculated (ThMax) and Actual (FCF) Lmax Differ. 2 Units
PLAT969_ALERT_5_G The 'Henn et al.' R-Factor-gap value .... 6.224 Note
Predicted wR2: Based on SigI**2 2.48 or SHELX Weight 14.20
PLAT978_ALERT_2_G Number C-C Bonds with Positive Residual Density. 21 Info
PLAT992_ALERT_5_G Repd & Actual _reflns_number_gt Values Differ by 3 Check

---

0 **ALERT level A** = Most likely a serious problem - resolve or explain
1 **ALERT level B** = A potentially serious problem, consider carefully
7 **ALERT level C** = Check. Ensure it is not caused by an omission or oversight
27 **ALERT level G** = General information/check it is not something unexpected

7 ALERT type 1 CIF construction/syntax error, inconsistent or missing data
5 ALERT type 2 Indicator that the structure model may be wrong or deficient
6 ALERT type 3 Indicator that the structure quality may be low
14 ALERT type 4 Improvement, methodology, query or suggestion
3 ALERT type 5 Informative message, check

It is advisable to attempt to resolve as many as possible of the alerts in all categories. Often the minor alerts point to easily fixed oversights, errors and omissions in your CIF or refinement strategy, so attention to these fine details can be worthwhile. In order to resolve some of the more serious problems it may be necessary to carry out additional measurements or structure refinements. However, the purpose of your study may justify the reported deviations and the more serious of these should normally be commented upon in the discussion or experimental section of a paper or in the "special_details" fields of the CIF. checkCIF was carefully designed to identify outliers and unusual parameters, but every test has its limitations and alerts that are not important in a particular case may appear. Conversely, the absence of alerts does not guarantee there are no aspects of the results needing attention. It is up to the individual to critically assess their own results and, if necessary, seek expert advice.

Publication of your CIF in IUCr journals

A basic structural check has been run on your CIF. These basic checks will be run on all CIFs submitted for publication in IUCr journals (*Acta Crystallographica*, *Journal of Applied Crystallography*, *Journal of Synchrotron Radiation*); however, if you intend to submit to *Acta Crystallographica Section C* or *E* or *IUCrData*, you should make sure that full publication checks are run on the final version of your CIF prior to submission.

Publication of your CIF in other journals

Please refer to the *Notes for Authors* of the relevant journal for any special instructions relating to CIF submission.

PLATON version of 02/02/2025; check.def file version of 02/02/2025

Datablock TS1-1769 - ellipsoid plot

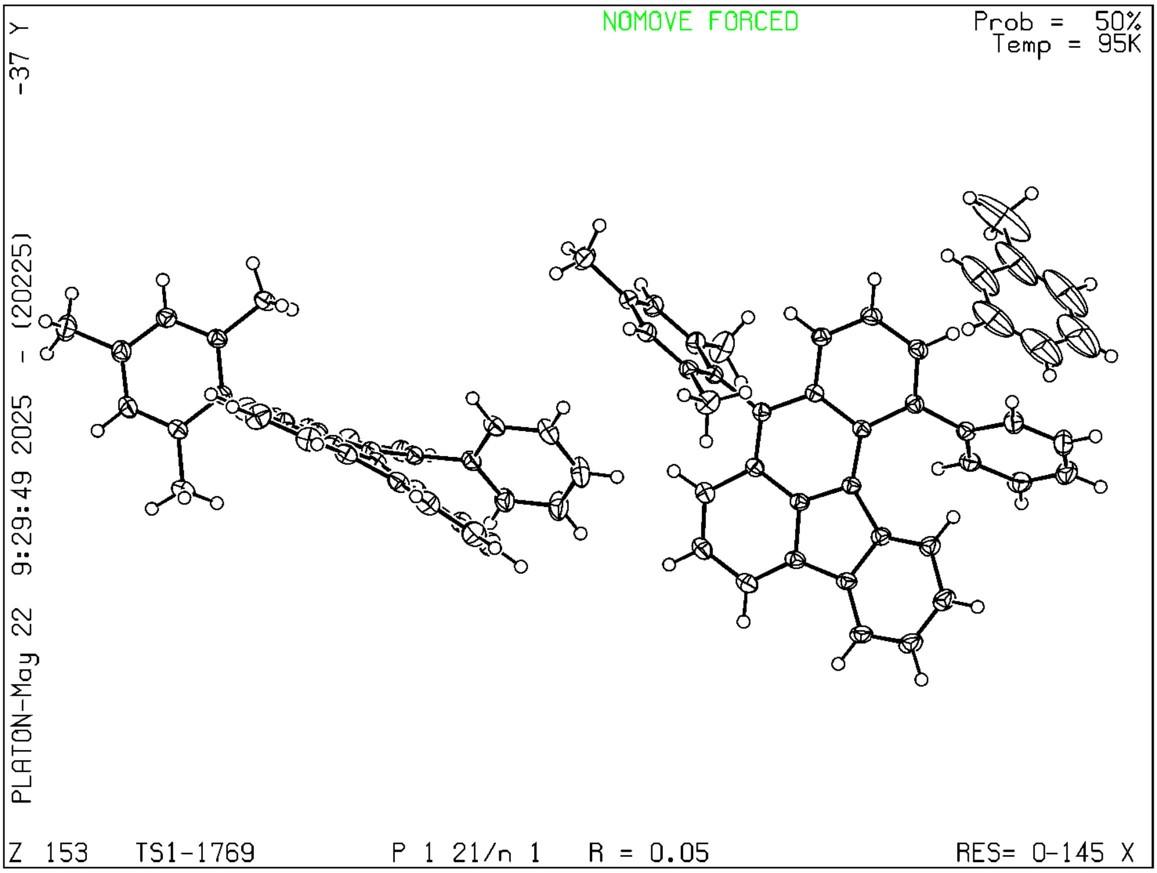

Supplement: Supplementary file 2 [file CHEM-32-e71449-s002.zip › Compound7_5_cifreport.pdf]
