# Supplementary material for: Cyclization of a 1,8‐Diphenylanthracene by Scholl Reactions to Form Five‐ and Seven‐Membered Ring Embedded Polycyclic Aromatic Hydrocarbons
Source: Chemistry. 2026 Jul 20;32(39):e71449. doi: 10.1002/chem.71449 (PMC13715047; doi:10.1002/chem.71449)

checkCIF/PLATON report

Structure factors have been supplied for datablock(s) TS-179-A-1

THIS REPORT IS FOR GUIDANCE ONLY. IF USED AS PART OF A REVIEW PROCEDURE FOR PUBLICATION, IT SHOULD NOT REPLACE THE EXPERTISE OF AN EXPERIENCED CRYSTALLOGRAPHIC REFEREE.

No syntax errors found. CIF dictionary Interpreting this report

Datablock: TS-179-A-1

Bond precision: C-C = 0.0020 A Wavelength=3/4

Cell:	a=7.119(7)	b=24.046(6)	c=26.053(6)
	alpha=90	beta=90	gamma=90
Temperature:	95 K		

	Calculated	Reported
Volume	4460(5)	4460(5)
Space group	P b c a	P b c a
Hall group	-P 2ac 2ab	-P 2ac 2ab
Moiety formula	C35 H24	2(C35 H24)
Sum formula	C35 H24	C70 H48
Mr	444.54	889.08
Dx,g cm-3	1.324	1.324
Z	8	4
Mu (mm-1)	0.082	0.083
F000	1872.0	1872.0
F000'	1872.79	
h,k,lmax	11,37,40	8,35,38
Nref	8688	7179
Tmin,Tmax	0.992,0.992	
Tmin'	0.992	

Correction method= Not given

Data completeness= 0.826 Theta(max)= 35.556

R(reflections)= 0.0560(5662) wR2(reflections)= 0.1555(7179)

S = 1.030 Npar= 319

The following ALERTS were generated. Each ALERT has the format
test-name_ALERT_alert-type_alert-level.
Click on the hyperlinks for more details of the test.

Alert level B

```
PLAT029_ALERT_3_B _diffrn_measured_fraction_theta_full value Low .      0.940 Why?
```

Alert level C

```
PLAT041_ALERT_1_C Calc. and Reported SumFormula    Strings  Differ     Please Check
              Calc: C35 H24
              Rep.: C70 H48
PLAT042_ALERT_1_C Calc. and Reported MoietyFormula Strings  Differ     Please Check
              Calc: C35 H24
              Rep.: 2(C35 H24)
PLAT052_ALERT_1_C Info on Absorption Correction Method   Not Given     Please Do !
PLAT906_ALERT_3_C Large K Value in the Analysis of Variance ......      5.184 Check
PLAT911_ALERT_3_C Missing FCF Refl Between Thmin & STh/L=    0.600        242 Report
                2  0  0,   4  0  0,   6  0  0,   8  0  0,   4  1  0,   6  1  0,
                8  1  0,   4  2  0,   6  2  0,   8  2  0,   4  3  0,   6  3  0,
                8  3  0,   0  4  0,   6  4  0,   8  4  0,   6  5  0,   8  5  0,
                6  6  0,   8  6  0,   8  7  0,   8  8  0,   8  9  0,   8 10  0,
                3  1  1,   4  1  1,   5  1  1,   6  1  1,   7  1  1,   8  1  1,
                4  2  1,   5  2  1,   6  2  1,   7  2  1,   8  2  1,   4  3  1,
                5  3  1,   6  3  1,   7  3  1,   8  3  1,   6  4  1,   7  4  1,
                8  4  1,   6  5  1,   7  5  1,   8  5  1,   6  6  1,   7  6  1,
                8  6  1,   7  7  1,   8  7  1,   7  8  1,   8  8  1,   7  9  1,
                8  9  1,   8 10  1,   3  0  2,   4  0  2,   5  0  2,   6  0  2,
                7  0  2,   8  0  2,   4  1  2,   5  1  2,   6  1  2,   7  1  2,
                8  1  2,   4  2  2,   5  2  2,   6  2  2,   7  2  2,   8  2  2,
                5  3  2,   6  3  2,   7  3  2,   8  3  2,   6  4  2,   7  4  2,
                8  4  2,   6  5  2,   7  5  2,   8  5  2,   7  6  2,   8  6  2,
                7  7  2,   8  7  2,   7  8  2,   8  8  2,   7  9  2,   8  9  2,
                5  1  3,   6  1  3,   7  1  3,   8  1  3,   5  2  3,   6  2  3,
```

Alert level G

```
ABSMU01_ALERT_1_G  Calculation of _exptl_absorpt_correction_mu
                not performed for this radiation type.
PLAT045_ALERT_1_G Calculated and Reported Z Differ by a Factor ...          2 Check
PLAT380_ALERT_4_G Incorrectly? Oriented X(sp2)-Methyl Moiety .....       C00Z Check
PLAT720_ALERT_4_G Number of Unusual/Non-Standard Labels ..........         59 Note
              C001    C002    C003    C004    C005    C006    H006    C007
              C008    C009    C00A    C00B    C00C    H00C    C00D    C00E
              H00E    C00F    C00G    H00G    C00H    C00I    H00I    C00J
              H00J    C00K    H00K    C00L    C00M    C00N    H00N    C00O
              H00O    C00P    H00P    C00Q    C00R    C00S    H00S    C00T
              H00T    C00U    H00U    C00V    H00V    C00W    H00W    C00X
              H00A    H00B    H00D    C00Y    H00F    H00H    H00L    C00Z
              H00M    H00Q    H00R
PLAT912_ALERT_4_G Missing # of FCF Reflections Above STh/L=  0.600        597 Note
PLAT950_ALERT_5_G Calculated (ThMax) and CIF-Reported Hmax Differ           3 Units
PLAT951_ALERT_5_G Calculated (ThMax) and CIF-Reported Kmax Differ           2 Units
PLAT952_ALERT_5_G Calculated (ThMax) and CIF-Reported Lmax Differ.          2 Units
```

```
PLAT956_ALERT_1_G Calculated (ThMax) and Actual (FCF) Hmax Differ          3 Units
PLAT957_ALERT_1_G Calculated (ThMax) and Actual (FCF) Kmax Differ          2 Units
PLAT958_ALERT_1_G Calculated (ThMax) and Actual (FCF) Lmax Differ.         2 Units
PLAT969_ALERT_5_G The 'Henn et al.' R-Factor-gap value ..........      5.116 Note
              Predicted wR2: Based on SigI**2  3.04 or SHELX Weight 15.10
PLAT978_ALERT_2_G Number C-C Bonds with Positive Residual Density.        25 Info
```

```
   0 ALERT level A = Most likely a serious problem - resolve or explain
   1 ALERT level B = A potentially serious problem, consider carefully
   5 ALERT level C = Check. Ensure it is not caused by an omission or oversight
  13 ALERT level G = General information/check it is not something unexpected

   8 ALERT type 1 CIF construction/syntax error, inconsistent or missing data
   1 ALERT type 2 Indicator that the structure model may be wrong or deficient
   3 ALERT type 3 Indicator that the structure quality may be low
   3 ALERT type 4 Improvement, methodology, query or suggestion
   4 ALERT type 5 Informative message, check
```

It is advisable to attempt to resolve as many as possible of the alerts in all categories. Often the minor alerts point to easily fixed oversights, errors and omissions in your CIF or refinement strategy, so attention to these fine details can be worthwhile. In order to resolve some of the more serious problems it may be necessary to carry out additional measurements or structure refinements. However, the purpose of your study may justify the reported deviations and the more serious of these should normally be commented upon in the discussion or experimental section of a paper or in the "special_details" fields of the CIF. checkCIF was carefully designed to identify outliers and unusual parameters, but every test has its limitations and alerts that are not important in a particular case may appear. Conversely, the absence of alerts does not guarantee there are no aspects of the results needing attention. It is up to the individual to critically assess their own results and, if necessary, seek expert advice.

Publication of your CIF in IUCr journals

A basic structural check has been run on your CIF. These basic checks will be run on all CIFs submitted for publication in IUCr journals (*Acta Crystallographica*, *Journal of Applied Crystallography*, *Journal of Synchrotron Radiation*); however, if you intend to submit to *Acta Crystallographica Section C* or *E* or *IUCrData*, you should make sure that full publication checks are run on the final version of your CIF prior to submission.

Publication of your CIF in other journals

Please refer to the *Notes for Authors* of the relevant journal for any special instructions relating to CIF submission.

PLATON version of 02/02/2025; check.def file version of 02/02/2025

Datablock TS-179-A-1 - ellipsoid plot

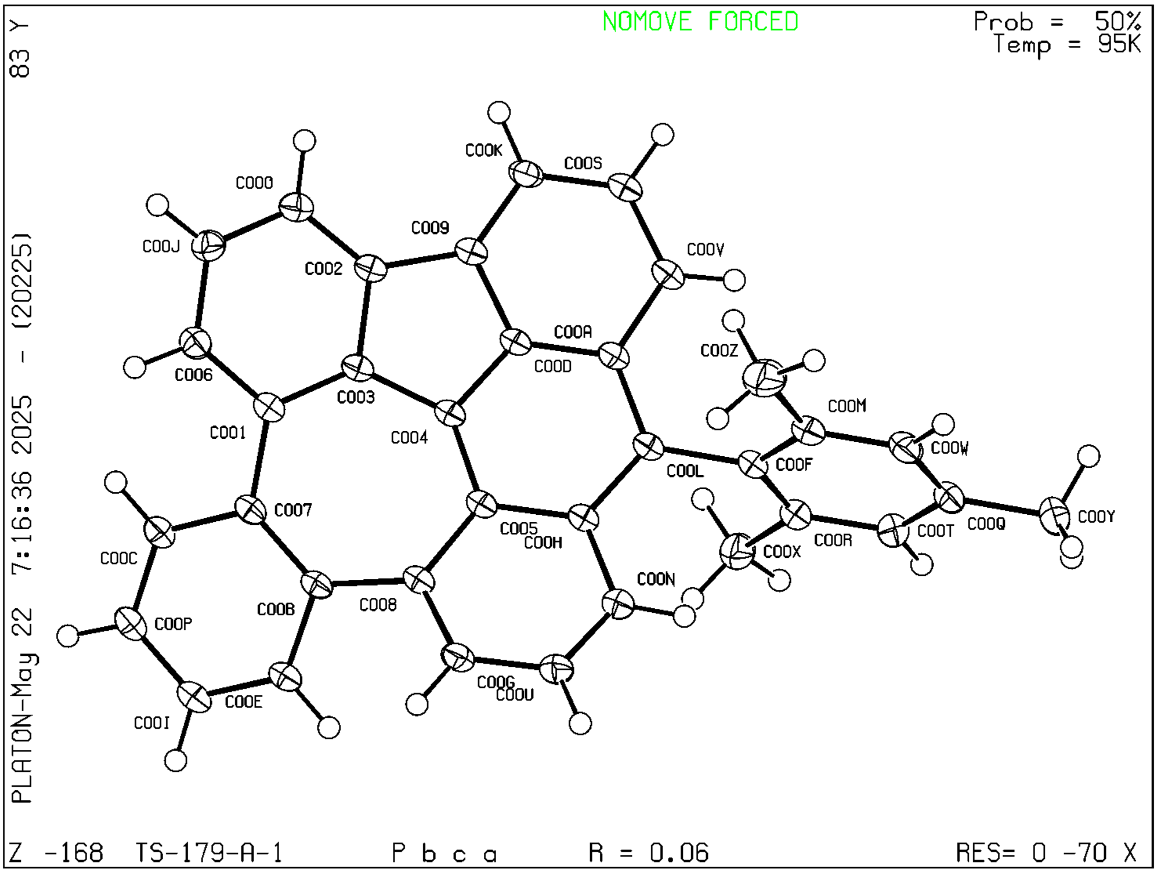

Supplement: Supplementary file 2 [file CHEM-32-e71449-s002.zip › Compound8_57_cifreport.pdf]
