# Supplementary material for: Cyclization of a 1,8‐Diphenylanthracene by Scholl Reactions to Form Five‐ and Seven‐Membered Ring Embedded Polycyclic Aromatic Hydrocarbons
Source: Chemistry. 2026 Jul 20;32(39):e71449. doi: 10.1002/chem.71449 (PMC13715047; doi:10.1002/chem.71449)

checkCIF/PLATON report

You have not supplied any structure factors. As a result the full set of tests cannot be run.

THIS REPORT IS FOR GUIDANCE ONLY. IF USED AS PART OF A REVIEW PROCEDURE FOR PUBLICATION, IT SHOULD NOT REPLACE THE EXPERTISE OF AN EXPERIENCED CRYSTALLOGRAPHIC REFEREE.

No syntax errors found. CIF dictionary Interpreting this report

Datablock: shelx

Bond precision:	C-C = 0.0016 A		Wavelength=3/4
Cell:	a=42.690(8)	b=3.818(2)	c=27.105(4)
	alpha=90	beta=112.484(2)	gamma=90
Temperature:	95 K		

	Calculated	Reported
Volume	4082(2)	4082(3)
Space group	C 2/c	C 1 2/c 1
Hall group	-C 2yc	-C 2yc
Moiety formula	C35 H22	C35 H22
Sum formula	C35 H22	C35 H22
Mr	442.53	442.52
Dx,g cm-3	1.440	1.440
Z	8	8
Mu (mm-1)	0.090	0.091
F000	1856.0	1856.0
F000'	1856.79	
h,k,lmax	66,5,42	63,4,40
Nref	7929	6868
Tmin,Tmax	0.991,0.991	
Tmin'	0.991	

Correction method= Not given

Data completeness= 0.866 Theta(max)= 35.534

R(reflections)= 0.0469(5921) wR2(reflections)= 0.1298(6868)

S = 1.044 Npar= 319

The following ALERTS were generated. Each ALERT has the format
test-name_ALERT_alert-type_alert-level.
Click on the hyperlinks for more details of the test.

Alert level B

```
PLAT029_ALERT_3_B _diffrn_measured_fraction_theta_full value Low .      0.943 Why?
```

Alert level C

```
DIFMX02_ALERT_1_C  The maximum difference density is > 0.1*ZMAX*0.75
            The relevant atom site should be identified.
PLAT052_ALERT_1_C Info on Absorption Correction Method   Not Given     Please Do !
PLAT097_ALERT_2_C Large Reported Max.  (Positive) Residual Density       0.53 eA-3
```

Alert level G

```
ABSMU01_ALERT_1_G  Calculation of _exptl_absorpt_correction_mu
                not performed for this radiation type.
PLAT128_ALERT_4_G Alternate Setting for Input Space Group     C2/c       I2/a Note
PLAT720_ALERT_4_G Number of Unusual/Non-Standard Labels ..........         57 Note
              C001    C002    C003    C004    C005    C006    C007    H007
              C008    C009    C00A    C00B    C00C    C00D    H00D    C00E
              H00E    C00F    C00G    C00H    C00I    C00J    C00K    C00L
              H00L    C00M    H00M    C00N    H00N    C00O    C00P    H00P
              C00Q    H00A    H00B    H00C    C00R    H00F    H00G    H00H
              C00S    H00S    C00T    H00T    C00U    H00U    C00V    H00V
              C00W    H00W    C00X    C00Y    H00K    H00I    H00J    C00Z
              H00Z
PLAT941_ALERT_3_G Average HKL Measurement Multiplicity ..........        3.0 Low
PLAT950_ALERT_5_G Calculated (ThMax) and CIF-Reported Hmax Differ          3 Units
PLAT952_ALERT_5_G Calculated (ThMax) and CIF-Reported Lmax Differ.         2 Units
```

0 **ALERT level A** = Most likely a serious problem - resolve or explain
1 **ALERT level B** = A potentially serious problem, consider carefully
3 **ALERT level C** = Check. Ensure it is not caused by an omission or oversight
6 **ALERT level G** = General information/check it is not something unexpected

3 ALERT type 1 CIF construction/syntax error, inconsistent or missing data
1 ALERT type 2 Indicator that the structure model may be wrong or deficient
2 ALERT type 3 Indicator that the structure quality may be low
2 ALERT type 4 Improvement, methodology, query or suggestion
2 ALERT type 5 Informative message, check

It is advisable to attempt to resolve as many as possible of the alerts in all categories. Often the minor alerts point to easily fixed oversights, errors and omissions in your CIF or refinement strategy, so attention to these fine details can be worthwhile. In order to resolve some of the more serious problems it may be necessary to carry out additional measurements or structure refinements. However, the purpose of your study may justify the reported deviations and the more serious of these should normally be commented upon in the discussion or experimental section of a paper or in the "special_details" fields of the CIF. checkCIF was carefully designed to identify outliers and unusual parameters, but every test has its limitations and alerts that are not important in a particular case may appear. Conversely, the absence of alerts does not guarantee there are no aspects of the results needing attention. It is up to the individual to critically assess their own results and, if necessary, seek expert advice.

Publication of your CIF in IUCr journals

A basic structural check has been run on your CIF. These basic checks will be run on all CIFs submitted for publication in IUCr journals (*Acta Crystallographica*, *Journal of Applied Crystallography*, *Journal of Synchrotron Radiation*); however, if you intend to submit to *Acta Crystallographica Section C* or *E* or *IUCrData*, you should make sure that full publication checks are run on the final version of your CIF prior to submission.

Publication of your CIF in other journals

Please refer to the *Notes for Authors* of the relevant journal for any special instructions relating to CIF submission.

PLATON version of 02/02/2025; check.def file version of 02/02/2025

Datablock shelx - ellipsoid plot

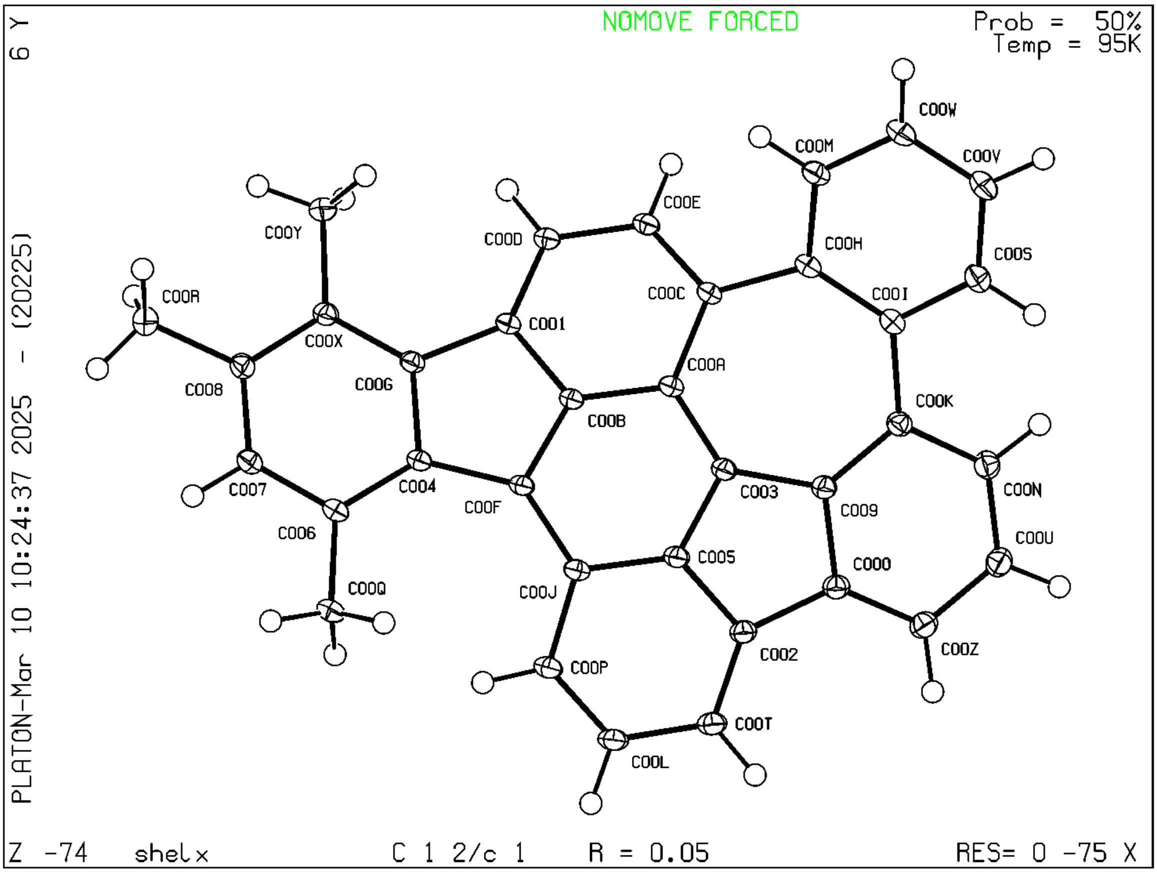

Supplement: Supplementary file 2 [file CHEM-32-e71449-s002.zip › Compound9_575_checkcif.pdf]
